# Genome-wide meta-analysis identifies multiple novel associations and ethnic heterogeneity of psoriasis susceptibility

**DOI:** 10.1038/ncomms7916

**Published:** 2015-04-23

**Authors:** Xianyong Yin, Hui Qi Low, Ling Wang, Yonghong Li, Eva Ellinghaus, Jiali Han, Xavier Estivill, Liangdan Sun, Xianbo Zuo, Changbing Shen, Caihong Zhu, Anping Zhang, Fabio Sanchez, Leonid Padyukov, Joseph J. Catanese, Gerald G. Krueger, Kristina Callis Duffin, Sören Mucha, Michael Weichenthal, Stephan Weidinger, Wolfgang Lieb, Jia Nee Foo, Yi Li, Karseng Sim, Herty Liany, Ishak Irwan, Yikying Teo, Colin T. S. Theng, Rashmi Gupta, Anne Bowcock, Philip L. De Jager, Abrar A. Qureshi, Paul I. W. de Bakker, Mark Seielstad, Wilson Liao, Mona Ståhle, Andre Franke, Xuejun Zhang, Jianjun Liu

**Affiliations:** 1 Institute of Dermatology and Department of Dermatology at No.1 Hospital, Anhui Medical University, Hefei, Anhui 230032, China; 2 State Key Laboratory Incubation Base of Dermatology, Ministry of National Science and Technology, Anhui Medical University, Hefei, Anhui 230032, China; 3 Key Lab of Dermatology (Anhui Medical University), Ministry of Education, Hefei, Anhui 230032, China; 4 Collaborative Innovation Center for Complex and Severe Skin Diseases, Anhui Medical University, Hefei, Anhui 230032, China; 5 Department of Human Genetics, Genome Institute of Singapore, A*STAR, Singapore 138672, Singapore; 6 Celera, Alameda, California 94502, USA; 7 Institute of Clinical Molecular Biology, Christian-Albrechts-University of Kiel, Schittenhelm Street 12, Kiel 24105, Germany; 8 Department of Epidemiology, Richard M. Fairbanks School of Public Health, Indiana University, Indianapolis, Indiana 46202, USA; 9 Melvin and Bren Simon Cancer Center, Indiana University, Indianapolis, Indiana 46202, USA; 10 Department of Dermatology, School of Medicine, Indiana University, Indianapolis, Indiana 46202, USA; 11 Genetic Causes of Disease Group, Centre for Genomic Regulation (CRG), Barcelona, Catalonia, E-08003, Spain; 12 Department of Experimental and Health Sciences, Universitat Pompeu Fabra, Barcelona, Catalonia, E-08003, Spain; 13 Hospital del Mar Medical Research Institute (IMIM), Barcelona, Catalonia, E-08003, Spain; 14 CIBER in Epidemiology and Public Health (CIBERESP), Barcelona, Catalonia, E-08003, Spain; 15 Unit of Dermatology and Venereology, Department of Medicine, Karolinska Institutet, Stockholm 17176, Sweden; 16 Rheumatology Unit, Department of Medicine Solna, Karolinska Institutet, Stockholm 17177, Sweden; 17 Department of Dermatology, University of Utah, Salt Lake, Utah 84132, USA; 18 Department of Dermatology, University Hospital Schleswig-Holstein, Christian-Albrechts-University, Kiel, Germany; 19 Institute of Epidemiology and Biobank PopGen, Christian Albrechts University, Kiel, Germany; 20 Departments of Statistics and Applied Probability, National University of Singapore, Singapore 138672, Singapore; 21 Department of Epidemiology and Public Health, National University of Singapore, Singapore 138672, Singapore; 22 National Skin Centre, Singapore 308205, Singapore; 23 Department of Dermatology, University of California San Francisco, San Francisco, California 94115, USA; 24 National Heart and Lung Institute, Imperial College, London SW3 6LY, UK; 25 Program in Medical and Population Genetics, Broad Institute, Cambridge, Massachusetts 02138, USA; 26 Program in Translational NeuroPsychiatric Genomics, Department of Neurology, Brigham & Women's Hospital, Harvard Medical School, Boston, Massachusetts 02115, USA; 27 Department of Epidemiology, Julius Center for Health Sciences and Primary Care, University Medical Center Utrecht, Utrecht, 3584 CG, The Netherlands; 28 Department of Medical Genetics, Center for Molecular Medicine, University Medical Center Utrecht, Utrecht, 3584 CG, The Netherlands; 29 Institute for Human Genetics, University of California San Francisco, San Francisco, California 94143, USA; 30 Saw Swee Hock School of Public Health, National University of Singapore, National University Health System, Singapore, 138672, Singapore; 31 School of Life Sciences, Anhui Medical University, Hefei, Anhui 230032, China

## Abstract

Psoriasis is a common inflammatory skin disease with complex genetics and different degrees of prevalence across ethnic populations. Here we present the largest trans-ethnic genome-wide meta-analysis (GWMA) of psoriasis in 15,369 cases and 19,517 controls of Caucasian and Chinese ancestries. We identify four novel associations at *LOC144817*, *COG6*, *RUNX1* and *TP63*, as well as three novel secondary associations within *IFIH1* and *IL12B*. Fine-mapping analysis of MHC region demonstrates an important role for all three *HLA* class I genes and a complex and heterogeneous pattern of *HLA* associations between Caucasian and Chinese populations. Further, trans-ethnic comparison suggests population-specific effect or allelic heterogeneity for 11 loci. These population-specific effects contribute significantly to the ethnic diversity of psoriasis prevalence. This study not only provides novel biological insights into the involvement of immune and keratinocyte development mechanism, but also demonstrates a complex and heterogeneous genetic architecture of psoriasis susceptibility across ethnic populations.

## Abstract (web summary)

Psoriasis is a common inflammatory skin disease with complex genetics and different degrees of prevalence across ethnic populations. Here Yin *et al*. conduct a large trans-ethnic genome-wide meta-analysis and identify novel loci that contribute to population-specific susceptibility.

## Graphical abstract

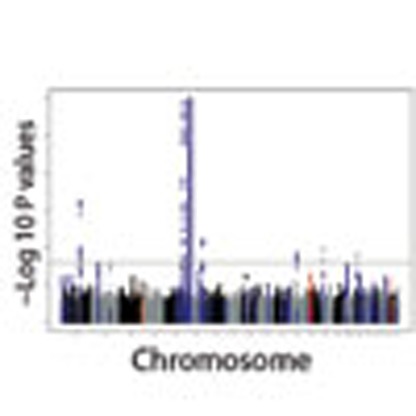

Psoriasis is a common immune-mediated chronic inflammatory disease characterized by hyperplasia, altered proliferation and differentiation of keratinocytes, vascular remodelling and inflammation in the skin, with musculoskeletal inflammation of the joints observed in up to 30% of patients^1^. Psoriasis shows a diverse prevalence across worldwide populations, 2.5% in Europeans, 0.05–3% in Africans and 0.1–0.5% in Asians^2^,^3^,^4^. Phenotypic heterogeneity of psoriasis has also been reported among ethnic populations, such as clinical manifestation, response to treatments and disease progression^3^,^4^,^5^,^6^.

Psoriasis has a high genetic predisposition with estimated heritability up to 80% (refs 1, 7). Forty-one susceptibility loci have been identified at genome-wide significance (*P*<5 × 10^−8^) mainly through genome-wide association studies (GWASs)^8^,^9^,^10^,^11^,^12^,^13^,^14^,^15^,^16^, but these susceptibility loci collectively only explain a limited fraction of the heritability of psoriasis^16^,^17^. In addition, few loci also show differential associations between ethnic populations, suggesting potential genetic heterogeneity of psoriasis, although most of the loci have not been systematically studied beyond the original population in which they are discovered^2^.

Recently, trans-ethnic genome-wide meta-analysis (GWMA) of the GWAS data sets of diverse populations have been performed to identify additional susceptibility loci and dissect validated loci through fine-mapping analysis, which can help to better understand the genetic architecture of complex diseases^18^,^19^,^20^,^21^,^22^,^23^,^24^,^25^,^26^. Trans-ethnic GWMA across diverse populations has not been performed in psoriasis, and the genetic heterogeneity of psoriasis across diverse ancestries has not yet been systematically investigated.

Here, we describe a large-scale trans-ethnic GWMA of psoriasis using multiple GWAS data sets as well as independent validation samples from Caucasian and Chinese populations. We identify four novel associations at *LOC144817*, *COG6*, *RUNX1* and *TP63*, as well as three novel secondary associations within *IFIH1* and *IL12B*. In addition, our study suggests population-specific effect or allelic heterogeneity for 11 loci, and indicates that these population-specific effects contribute significantly to the ethnic diversity of psoriasis prevalence. Together, these results provide novel biological insights into the involvement of immune and keratinocyte development mechanism, but also demonstrate a complex and heterogeneous genetic architecture of psoriasis susceptibility across ethnic populations.

## Results

### Genome-wide Meta-Analysis

**Figure 1:**
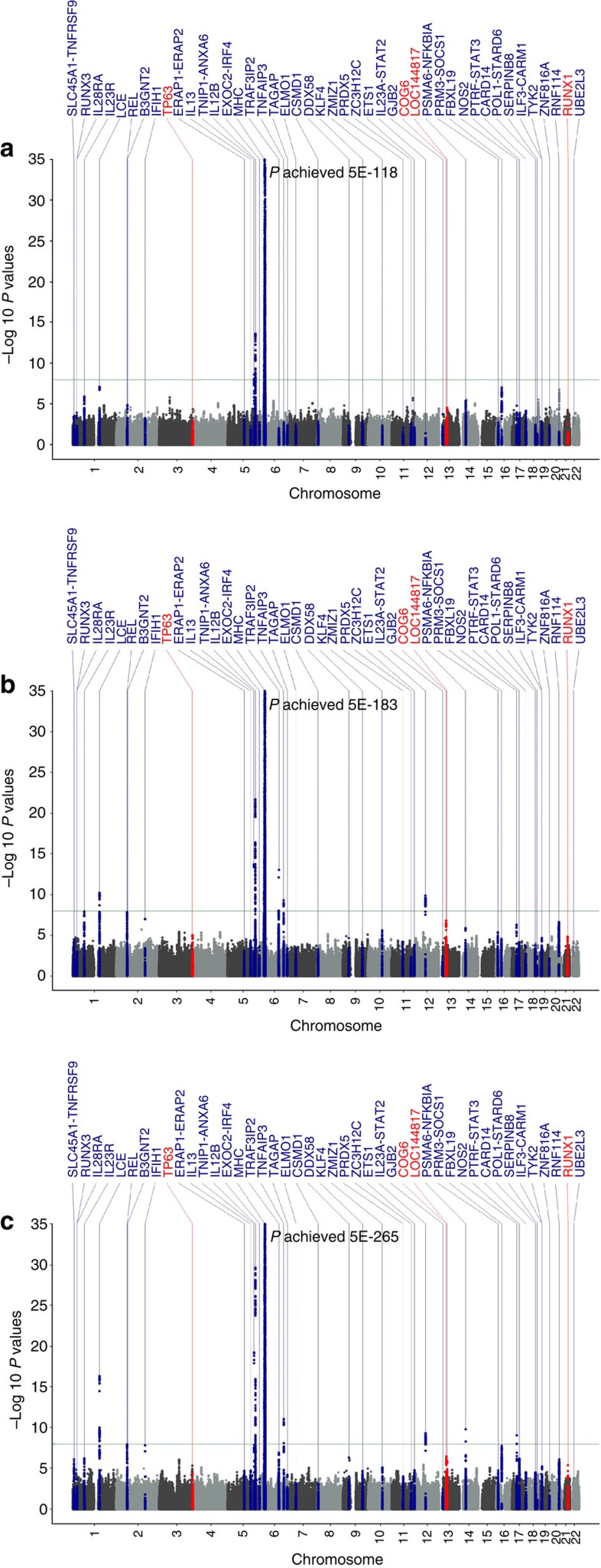
Manhattan plot of single SNP association test results. (**a**) Chinese GWAS meta-analysis with 1,588 cases and 3,546 controls; (**b**) Caucasian GWAS meta-analysis with 3,496 cases and 5,186 controls; (**c**) trans-ethnic GWAS meta-analysis with 5,084 cases and 8,732 controls. The *x*-axis indicates the chromosomal position. The *y*-axis indicates the –log10 *P* values of genome-wide SNP associations from each GWAS meta-analysis using logistic regression. The horizontal green line represents the genome-wide significance threshold of *P*=5.0 × 10^−8^. Blue dots indicate the association results of SNPs within the 41 known psoriasis risk loci; red dots indicate the association results of SNPs within the four new psoriasis risk loci; grey dots indicate the association results of SNPs outside the 45 psoriasis risk loci.

We carried out GWMA of psoriasis using seven independent GWAS data sets, including five published ones (four Caucasians and one Chinese)^11^,^15^,^16^ and two unpublished ones (one Caucasian and one Chinese; Table 1). The combined data set consists of a total number of 5,084 psoriatic cases and 8,732 controls, with 3,496 cases and 5,186 controls from Caucasian population and 1,588 cases and 3,546 controls from Chinese population. To combine these seven independent data sets that were generated by using different genotyping platforms and further enhance the coverage of genetic variants for association analysis, whole-genome imputation was performed in each of the seven data sets separately using the 1000 Genomes Project data set (Phase I, Dec. 2010 version) as the reference panel. After applying stringent quality controls (QCs), we tested 4,778,154, 4,562,294 and 3,621,551 single-nucleotide polymorphisms (SNPs) for association in the Caucasian, Chinese and combined cohorts, respectively (Table 1). Both the quantile–quantile plots and genomic inflation factors (*λ*_GC_) of the genome-wide test statistic (1.017 for Caucasian cohort, 1.01 for Chinese cohort and 1.011 for the combined cohort) demonstrated that the three genome-wide association analyses had negligible inflation because of population stratification (Table 1 and Supplementary Fig. 1). Meanwhile, the quantile–quantile and Manhattan plots of the three genome-wide analyses showed an excess of SNPs with small *P* values (<1 × 10^−4^ from the logistic regression analysis in individual ethnic and combined data sets) after removing the SNPs within 41 known loci, suggesting additional associations (of more modest effect) may be identified with further validation (Fig. 1 and Supplementary Fig. 1). All the known loci showed supporting evidences of association with nominal significance (*P*<0.05) in at least one genome-wide analysis, except *CSMD1* and *SERPINB8* that did not show any association, likely due to the insufficient statistical power (<30%) of the current study for detecting their associations (Supplementary Data 1).

### Validation Analysis of Novel Associations

We carried out the validation study of novel associations suggested by GWMA. Forty-three novel SNPs with suggestive association (*P*<5.00 × 10^−5^ from the logistic regression analysis) in at least one of the three discovery analyses (Caucasian, Chinese and Combined) were genotyped in additional two independent validation cohorts totaling 10,285 psoriatic cases and 10,785 controls, with 5,134 cases and 5,633 controls from Caucasian and 5,151 cases and 5,152 controls from Chinese populations (Table 1 and Supplementary Data 2). In the Caucasian samples, four novel associations were confirmed at genome-wide significance with consistent effects between the discovery and validation samples: rs9533962 (odds ratio (OR)=1.14, *P*=1.93 × 10^−8^, *LOC144817*), rs34394770 (OR=1.16, *P*=2.65 × 10^−8^, *COG6*), rs8128234 (OR=1.17, *P*=3.74 × 10^−8^, *RUNX1*) and rs28512356 (OR=1.17, *P*=4.31 × 10^−8^, *TP63*; Table 2 and Supplementary Figs 2 and 3). In the Chinese samples, all the four SNPs showed the consistent effect of association, but only rs34394770 and rs9533962 achieved nominal significance (*P*=4.63 × 10^−2^, 2.25 × 10^−4^, respectively). Both SNPs achieved genome-wide significance in the combined cohort (*P*=4.87 × 10^−8^, 7.53 × 10^−11^; Table 2 and Supplementary Figs 2 and 3). However, the other two novel SNPs did not achieve nominal significant association in Chinese samples, although the Chinese samples have sufficient power (statistic power>90%) to detect their effects observed in Caucasian population and was adequately powered to detect effect size (for example, ORs) as small as 1.15–1.25 (Supplementary Data 1 and Supplementary Table 1). No other SNPs selected from the discovery analyses in the Chinese and combined samples were validated.

Giving that all the newly discovered SNPs are noncoding variants, we have investigated the regulatory functions of these SNPs by analysing the information from the HaploReg (v2) and eQTL databases. The four novel SNPs were enriched in the enhancers of epidermal keratinocytes, and resided in known DNAse I hypersensitivity in multiple cell lines including normal human epidermal keratinocytes (Supplementary Table 2). And it was shown that three linked SNPs with rs34394770 conferred eQTL effect for *COG6* in monocytes (Supplementary Table 3).

**Figure 2:**
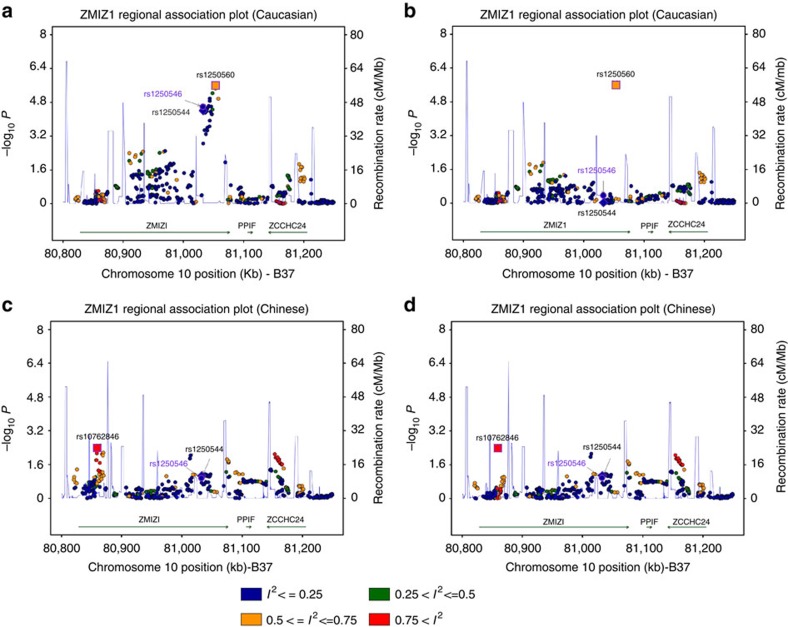
The regional association plots of *ZMIZ1* showing locus heterogeneity. The relative location of annotated genes and the direction of transcription are shown in the lower portion of the figure, and the chromosomal position is shown on the *x* axis. The blue line shows the recombination rate (estimated from HapMap data of CEU, CHB and combined population) across the region (right *y* axis), and the left *y* axis shows the significance of the SNP associations (−log10 P). The square indicates the SNPs for conditional analysis (these are the top or secondary SNPs); the circle labelled with rs IDs are reported psoriasis susceptibility SNPs. All circles and squares are colour filled based on the heterogeneity results (*I*^2^) in our trans-ethnic meta analysis. (**a**,**b**) Unconditional and conditional logistic association results of Caucasian, (**c**, **d**) unconditional and conditional logistic association results of Chinese.

We also searched for independent secondary associations within 45 previously and newly confirmed susceptibility loci in the discovery samples through conditional logistic regression analysis using the leading SNP within each locus as covariate (Fig. 2 and Supplementary Figs 3–5). First, we discovered three independent associations within *IL12B* locus at two novel SNPs, rs4921493 (*P*_condition_=6.80 × 10^−13^) and rs2853694 (*P*_condition_=1.22 × 10^−9^) and the previously reported rs7709212 (OR=1.38, *P*=2.44 × 10^−30^) in the combined discovery samples, whose independent effects were also confirmed by multivariate analysis (Supplementary Table 4a). The three SNPs are in low or moderate linkage disequilibrium (LD) (Supplementary Fig. 3e and Supplementary Table 4b) and showed consistent associations between the Caucasian and Chinese samples (Supplementary Table 4a). In addition, haplotype analysis also supported independent effects of the three SNPs (Supplementary Table 4c). Second, we discovered a novel independent SNP within the *IFIH1* locus. In Caucasian samples, the conditional analysis on the top SNP rs1990760 (OR=1.20, *P*=3.21 × 10^−13^) revealed a novel independent association at rs3747517 (OR_condition_=0.75, *P*_condition_=2.87 × 10^−11^), although rs3747517 did not show association by itself (OR=1.04, *P*=0.15; Supplementary Fig. 3f and Supplementary Table 5a). Consistently, rs3747517 only showed significant association (OR_condition_=0.80, *P*_condition_=5.16 × 10^−9^) in the Chinese cohort after conditioning on rs1990760 (Supplementary Fig. 3f and Supplementary Table 5a). The interactive effect between the two SNPs was confirmed by haplotype association analysis showing that only the haplotype 2 carrying the C alleles of the two SNPs showed strong protective effect (Supplementary Table 5b). In addition, our analysis showed that conditioning on rs1990760 can abolish the two previously reported independent associations at rs2111485 and rs17716942 (Supplementary Table 5a). These results suggest that both rs1990760 and rs3747517 tag the association effect of this haplotype in Caucasian and Chinese populations, and true causal variant carried by this haplotype remains to be discovered. In addition, we have also confirmed the previously reported independent associations at rs2910686 and rs30376 within *ERAP1/ERAP2* locus^16^ in the Caucasian GWAS samples (Supplementary Table 6 and Supplementary Fig. 3g). However, we did not detect the independent association effect of rs2910688 (OR=0.99, *P*=0.67) in the Chinese GWAS samples, although there was sufficient power to detect the effect of the same size (power=0.98 at *P*=0.05). Another six suggestive secondary associations failed to be validated or reach genome-wide significance (Supplementary Data 3).

### Fine Mapping Analysis of HLA Associations

To better understand the strong and extensive association within the major histocompatibility complex (MHC) region (chr6: 20–40 Mb, build 36; Supplementary Fig. 5), we performed a fine-mapping analysis of the region by imputing classical alleles and coding variants of *HLA* molecules and untyped SNPs in the Caucasian and Chinese discovery cohorts separately. As expected, the HLA-C*06:02 allele showed the strongest association within the region in both Caucasian (*P*=3.05 × 10^−167^) and Chinese (*P*=2.09 × 10^−101^). Stepwise conditional analysis revealed additional independent associations at the penta-allelic amino-acid polymorphism at the position 67 of *HLA-B* (*HLA-B_AA_67*) (*P*_condition_=4.31 × 10^−25^ after conditioning on HLA-C*06:02), HLA-A*02:01 (*P*_condition_=3.62 × 10^−10^ after conditioning on both HLA-C*0602 and *HLA-B_AA_67*) and rs9265656 (*P*_condition_=5.27 × 10^−9^ after conditioning on the three *HLA* variants) in the Caucasian cohort (Supplementary Table 7). Rs9265656 tags (*r*^2^=0.78) the classical allele HLA-B*07 (*P*_condition_=1.13 × 10^−5^ after conditioning on the three *HLA* variants above) and did not show any eQTL effect. In the Chinese population, the stepwise conditional analysis revealed additional independent associations at HLA-A*02:07 (*P*_condition_=5.30 × 10^−40^ after conditioning on HLA-C*06:02), amino-acid position 67 of *HLA-B* (*P*_condition_=8.96 × 10^−25^ after conditioning on HLA-C*06:02 and HLA-A*02:07), the quad-allelic amino-acid position 114 of *HLA-A* (*P*_condition_=6.04 × 10^−13^ after conditioning on three *HLA* variants) and rs3131857 (*P*_condition_= 9.76 × 10^−8^ after conditioning on all four *HLA* variants; Supplementary Table 7). Rs3131857 tags (*r*^2^=0.72) the biallelic amino-acid polymorphism at the position 144 of *HLA-A* (*HLA-A_AA_144*) (*P*_condition_=1.55 × 10^−6^ after conditioning on the prior four *HLA* variants) and did not show any eQTL effect. Beyond these *HLA* variants, no other *HLA* variants or SNPs showed independent association with *P*<10^−7^ (Supplementary Table 7 and Supplementary Fig. 5).

Although the HLA-C*06:02 and the *AA* position 67 of *HLA-B* appear to be shared between Caucasian and Chinese populations, the other independent *HLA* risk variants differ between the two populations. HLA-A*02:07 shows a strong association in Chinese but is very rare or absent in Europeans, whereas HLA-B*07 shows a strong association in Caucasians but is in turn very rare in Chinese. The other *HLA* variants are common (>10%) in both Caucasian and Chinese, but show population-specific associations, HLA-A*02:01 for Caucasian and the *AA* positions 114 and 144 of *HLA-A* for Chinese. Our findings indicate that all three major *HLA* class I genes may play an important role in psoriasis, and more importantly, illustrate a complex and heterogeneous pattern of *HLA* associations between Caucasian and Chinese populations.

### Analysis of Ethnic Heterogeneity

To further investigate the ethnic heterogeneity of psoriasis susceptibility, we compared the association signals of 44 confirmed (40 previously reported and 4 newly discovered) non-MHC loci between the Caucasian and Chinese cohorts, searching for the loci where association effects are mapped to different independent SNPs in two populations (allelic heterogeneity) or only detected in one population (locus heterogeneity). Of the 44 loci, we did not observe evidence of genetic heterogeneity among the independent samples of each ethnic population, but found the evidence of population-specific effect at 10 loci (*ELMO1, ERAP2, PRDX5, PRM3.SOCS1, RNF114, RUNX1, TP63, TRAF3IP2, TYK2* and *ZMIZ1*), where the association effect was only detected in the Caucasian samples (*P*_corrected_<0.05 after correction for 44 independent SNPs tested), but not in the Chinese GWAS samples (*P*≥0.1; Supplementary Data 1). For each of the nine loci, the Chinese samples provide sufficient statistical power (>95%) for detecting the association effect observed in the Caucasian samples at nominal significance (*P*<0.05), and the OR estimate was significantly different between the two populations (*P*_Q_<0.05). Regional plots of association results clearly demonstrate that besides the top Caucasian SNPs, there are no additional associations within the effective LD of these nine loci in the Chinese samples (Fig. 2 and Supplementary Figs 3 and 4). We also constructed a genetic risk score of these ten loci (count of risk alleles carried by an individual) and evaluated its association. Although this genetic risk score shows a highly significant association in Caucasian (*P*=3.94 × 10^−36^), there is no evidence of association in the Chinese population (*P*=0.21; Fig. 5). Taken together, these results provide strong evidence for the population-specific effects of these loci in the Caucasian population.

We have also estimated the contribution of these Caucasian-specific loci to the prevalence difference of psoriasis between the Caucasian and the Chinese populations. By assuming the independent and multiplicative effects of all the ten loci in Caucasians and no genetic effects in Chinese population (OR=1), our analysis indicated that the cumulative effects of these Caucasian-specific loci could explain up to 82.83% of the prevalence difference of psoriasis between the Caucasian and the Chinese populations. Because our Chinese samples only have sufficient power (statistic power>90%) to detect genetic effect of OR=1.15 or higher, our analysis may have overestimated the contribution of these loci to the prevalence difference by assuming that the ORs of these loci in Chinese population equal to 1. Our analysis, however, has clearly demonstrated that the cumulative genetic effects of those Caucasian-specific loci make a significant contribution to the prevalence difference of psoriasis between the Caucasian and the Chinese populations.

**Figure 3:**
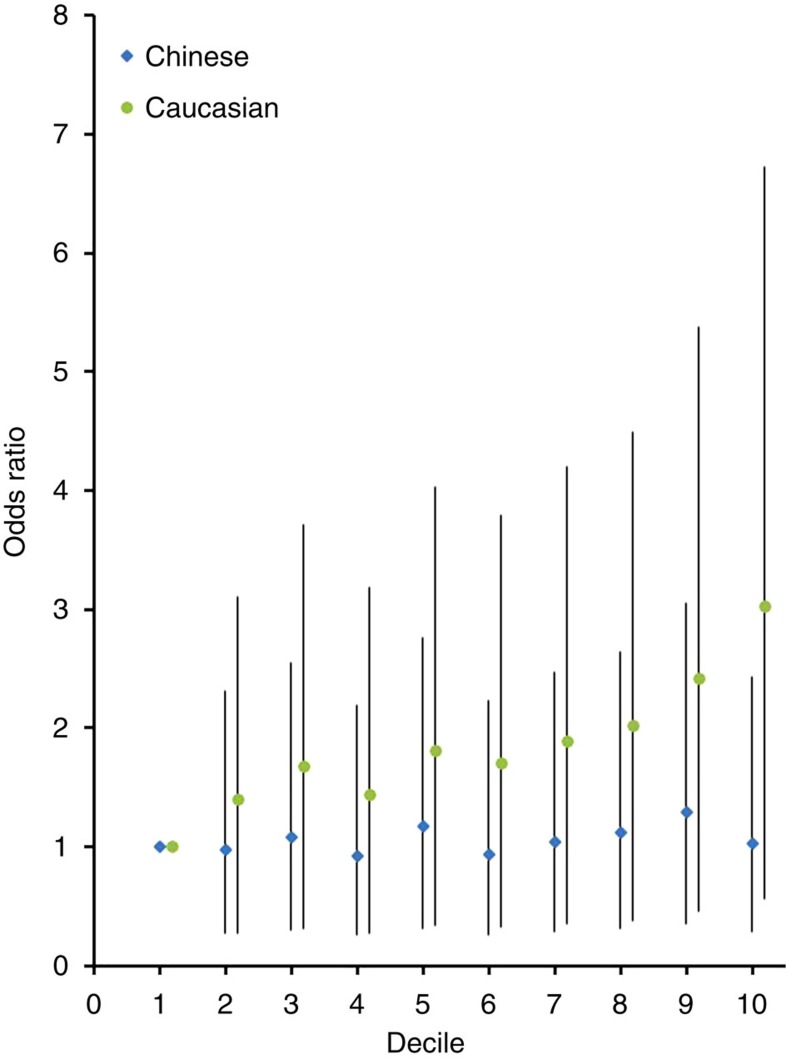
Odds ratios by the decile of polygenic risk score estimated based the top SNPs within the ten Caucasian-specific loci. The polygenic risk score (PRS) was calculated based on the ten Caucasian-specific SNPs as described (online Methods). The PRS were converted to deciles (1=lowest, 10=highest RPS), and nine dummy variables created to contrast deciles 2–10 to decile 1 as the reference. Odds ratios and 95% confidence intervals (error bars) were estimated using logistic regression with principal components to control for population stratification.

In addition, the association evidences within the *IL23R* locus were shown to be consistent with allelic heterogeneity between Caucasian and Chinese. There were two independent associations at rs2295359 and rs12564022 in the Caucasian samples (OR_condition_=1.15, *P*_condition_=8.7 × 10^−8^ and OR_condition_=1.23, *P*_condition_=9.0 × 10^−14^ respectively), but only one association at rs2295359 (OR=1.15, *P*=3.62 × 10^−8^) in the Chinese (Fig. 3 and Supplementary Data 4). The risk T allele of rs12564022 is more frequent in Chinese than Caucasians, but did not show independent association in Chinese. The haplotypes of the two SNPs showed different associations between the two populations: although the haplotype GC showed similar protective effect in both populations, the other two common haplotypes showed population-specific risk effect with the GT showing risk effect in Caucasian and the AC showing risk effect in Chinese (Supplementary Table 8). The diverse patterns of associations are consistent with different risk variants and thus allelic heterogeneity between the two populations.

**Figure 4:**
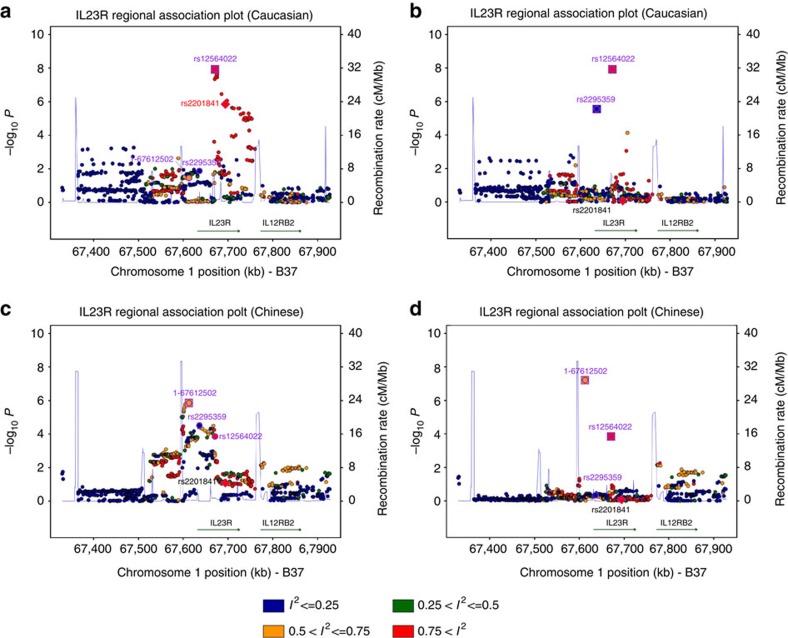
The regional association plots of *IL23R* showing allelic heterogeneity. The relative location of annotated genes and the direction of transcription are shown in the lower portion of the figure, and the chromosomal position is shown on the *x* axis. The blue line shows the recombination rate (estimated from HapMap data of CEU, CHB and Combined population) across the region (right *y* axis), and the left *y* axis shows the significance of the SNP associations (−log10 P). The square indicates the SNPs for conditional analysis (these are the top or secondary SNPs); the circle labelled with rs IDs are reported psoriasis susceptibility SNPs. All circles and squares are colour filled based on the heterogeneity results (*I*^2^) in our trans-ethnic meta analysis. (**a**,**b**) Unconditional and conditional logistic association results of Caucasian, (**c**, **d**) unconditional and conditional logistic association results of Chinese.

**Figure 5:**
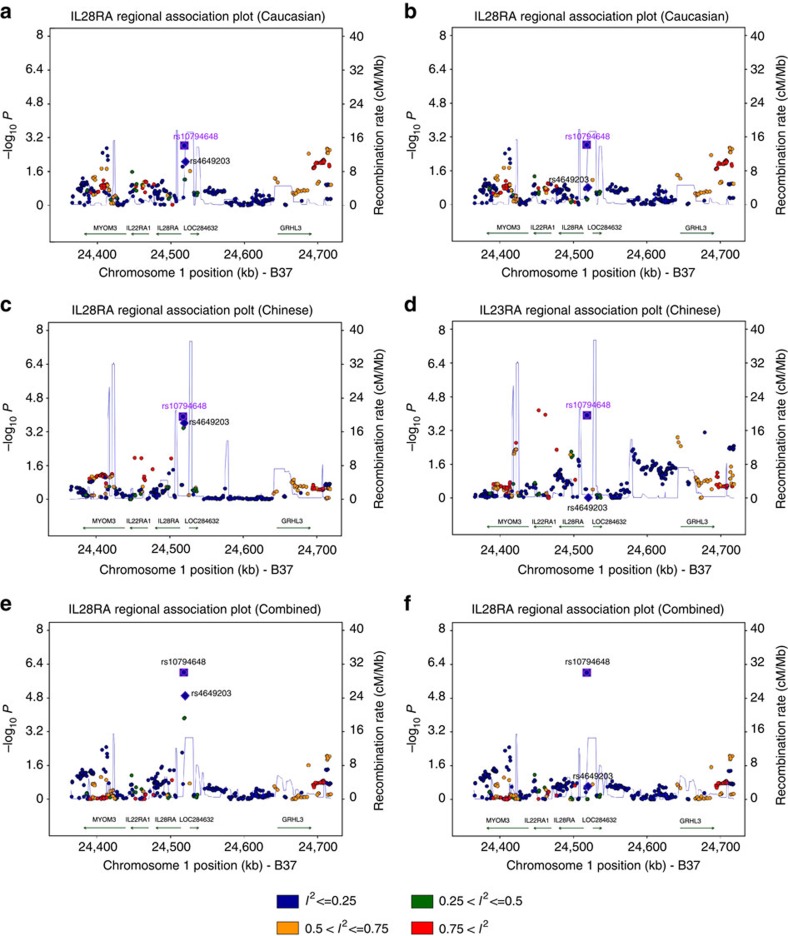
The regional association plots of *IL28RA* shared locus without heterogeneity. The relative location of annotated genes and the direction of transcription are shown in the lower portion of the figure, and the chromosomal position is shown on the *x* axis. The blue line shows the recombination rate (estimated from HapMap data of CEU, CHB and combined population) across the region (right *y* axis), and the left *y* axis shows the significance of the SNP associations (−log10 *P*). The square indicates the SNPs for conditional analysis (these are the top or secondary SNPs); the circle labelled with rs IDs are reported psoriasis susceptibility SNPs. All circles and squares are colour filled based on the heterogeneity results (*I*^2^) in our trans-ethnic meta analysis. (**a**,**b**) Unconditional and conditional logistic association results of Caucasian, (**c**,**d**) unconditional and conditional logistic association results of Chinese, (**e**,**f**) unconditional and conditional logistic association results of trans-ancestry combined sample.

For the remaining 27 loci, consistent association (OR in the same direction and without evidence of heterogeneity) was observed at either the same SNP or SNPs in high LD between the Caucasian and Chinese populations, and the meta-analysis of the combined Caucasian and Chinese samples revealed stronger evidence for association than the two individual samples (under a fixed effects model; Fig. 4, Supplementary Data 1 and Supplementary Figs 3, 4 and 6). Although not evidence of heterogeneity for the OR estimate, we observed significant frequency difference (*P*_Q_<0.05) for three SNPs within *COG6*, *FBXL29* and *IL23A/STAT2* between Caucasian and Chinese populations (Supplementary Fig. 7). In addition, we constructed a genetic risk score of the 30 SNPs in these 27 loci and found that the genetic risk score showed a significant difference among 52 Human Genome Diversity Panel (HGDP) populations and a significant correlation with the longitude of population, suggesting that these loci may be under selection and polygenetic adaptation to local environment (Supplementary Fig. 8). In addition, it is also interesting to note that the populations of European origin show higher risk score than the ones of Asian origin (Supplementary Fig. 8), which is consistent with the known higher disease prevalence in Europeans than Asians.

## Discussion

*TP63* is a strong candidate within the locus 3q11.2. *TP63* is a key regulator of mammalian epidermal stratification and keratinocyte proliferation and differentiation process^27^,^28^ and is expressed significantly in human skin and keratinocyte (Supplementary Tables 9 and 10). The discovery of *TP63* as a novel susceptibility locus provides additional biological insight into the role of keratinocyte proliferation and differentiation in the development of psoriasis. It is also interesting to note that *TP63* has also been discovered as susceptibility gene for lung and bladder cancers as well as immune and inflammatory response^29^,^30^,^31^,^32^,^33^. Rs8128234 on 21q22 is located within *RUNX1*. *RUNX1* encodes the alpha subunit of core-binding factor, which is a heterodimeric transcription factor that binds to the core element of many enhancers^34^. *RUNX1* gene has been shown to regulate alpha-beta T-cell differentiation and epidermis development (Supplementary Table 9)^35^,^36^, which are both critical for psoriasis development, and has been implicated in multiple autoimmune-related disease phenotypes including psoriasis^37^,^38^. Rs9533962 is located in long intergenic non-protein coding RNA *LOC144817* (ref. 39). And rs34394770 is an intronic variant within *COG6* that encodes a subunit of conserved oligomeric Golgi complex that is required for maintaining normal structure and activity of the Golgi apparatus^40^. Rs34394770 is in high LD with previously reported rs7993214 in Caucasian population (*r*^2^>0.98, *D*′>0.99)^41^, and our study has therefore confirmed the previously suggested association of *COG6* at genome-wide significance. The enrichment analysis of the previously and newly discovered loci has implicated a number of biological processes (gene ontology (GO) terms), which suggest the important roles of immune system and transcriptional regulation in psoriasis development (Supplementary Table 11).

We confirmed that there were multiple susceptible signals for psoriasis in MHC region. Besides HLA-C*0602, it was shown that common risk amino acid of *HLA-B* at position 67 in both Caucasian and Chinese, which was also consistent with the finding in a recent study^42^. We observed the significant allele frequency difference of some *HLA* risk alleles, such as HLA-A*02:07 and HLA-B*07, between Caucasian and Chinese populations, which could be a result of strong selection against diverse pathogens between the two ethnic populations^43^.

We also observed the population-specific effects of the AA positions 114 and 144 of *HLA-A* as well as another 10 non-MHC loci. These population-specific effects suggest a substantial genetic heterogeneity of psoriasis susceptibility between ethnic populations, which could be a result of different evolutionary process and environmental exposures that were experienced by two populations. Such population-specific effects could also be a reflection of potential clinical differences (such as clinical subtypes and family history) between Caucasian and Chinese patients used in the current study, but such a impact should be limited due to the fact that all the Caucasian and Chinese patients used in the current study were recruited by using the same criteria. Further stratified studies, such as by clinical subtypes and family history, can help to gain better understanding on the genetic heterogeneity of psoriasis susceptibility between Caucasian and Chinese populations.

In summary, we have conducted the first large-scale trans-ethnic GWMA in psoriasis and discovered seven novel associations, including four novel susceptibility loci and three novel independent associations within previous known loci. The new loci implicate additional players of the immune and keratinocyte development mechanism of psoriasis. In addition, we have investigated the genetic heterogeneity of psoriasis susceptibility by directly comparing the evidences of association of all the 45 confirmed loci between Caucasian and Chinese populations. Although many loci show consistent associations between the two ethnic populations, Caucasian-specific effect was observed for ten loci whose effects contribute significantly to the higher prevalence of psoriasis in Caucasian than in Chinese population. And the comparison of the shared loci across world populations has provided further evidence for the contribution of genetic susceptibility loci to disease prevalence difference between populations. In conclusion, our trans-ethnic genome-wide study has advanced the understanding of the genetic architecture of psoriasis susceptibility by discovering novel associations as well as revealing genetic heterogeneity across different ethnic populations.

## Methods

### Study subjects

The samples included in the five GWAS (University of California (UC), The Genetic Association Information Network (GAIN), Kiel, Genizon and Anhui) were previously published^15^,^16^. The UC data set included 202 psoriatic cases and 492 controls of European ancestry. The data set used for the analyses in GAIN of GWAS stage were obtained from the database of Genotype and Phenotype at http://www.ncbi.nlm.nih.gov/gap through database of Genotype and Phenotype accession number (phs000019.v1.p1). The Kiel sample included 471 cases and 1,129 controls, and the Genizon data from Canada included 1,755 samples. Anhui data set was comprised of 1,139 cases and 1,112 healthy controls in Han Chinese (Table 1). KI and Singapore data sets included samples of European descent from Sweden and Han Chinese from Singapore, respectively. The Singapore study included additional controls from Singapore Prospective Study Program (SP2). Replication analysis were performed in seven independent samples, six from populations of European descent (UC, KI, Harvard, Spain, Celera, Kiel) and one from Chinese population (Anhui). Recruitment of participants for all studies was approved by the local institutional ethics review boards, Institute of Dermatology, Anhui Medical University, National Skin Centre and National University of Singapore, Christian-Albrechts-University of Kiel, University of California San Francisco, Department of Medicine, Karolinska Institutet, Celera, Brigham & Women's Hospital, Harvard Medical School and Centre for Genomic Regulation (CRG), in adherence with the Declaration of Helsinki Principles. DNA was isolated from blood using standard methods.

### Genome-wide genotyping analysis

The genotyping and the QC procedures of the five published GWAS data sets can be found in the previous publications^15^,^16^. The cases of the Singapore GWAS data set were genotyped by using the Illumina Human550 BeadChip, whereas the controls were genotyped by using Illumina Human550, Illumina Human610 Quad and 1Mduo3 BeadChip. The cases and controls of the KI data set were genotyped by using Illumina Human Hap550 BeadChip. Standard QC procedures were applied as previously in Singapore and KI cohorts^15^. Briefly, samples with call rate of <0.95 and SNPs with mean BeadStudio GenCall score of <0.7, call rate of<0.95, Hardy–Weinberg equilibrium (HWE) *P* value of <1 × 10^−6^ or minor allele frequency (MAF) of <0.01 were excluded. Cryptic relatedness between individuals was identified through a full identity-by-state matrix. Population substructure of the Singapore and KI data sets was ascertained using principal components analyses (PCAs) with the EIGENSTRAT programme^44^ with respect to four population panels in the HapMap samples (CEU, CHB, JPT, YRI). The final Singapore and KI data sets consisted of 461,696 SNPs in 2,883 samples and 522,758 SNPs in 1,925 samples, respectively (Table 1).

### Whole-genome imputation

Whole-genome imputation was performed by using the IMPUTE version 2 (ref. 45), and the East Asia (ASN) and Europe (EUR) haplotypes of the 1000 Genomes Project reference panel (Dec 2010 version) were used as reference for imputation of Chinese (ASN) and European (EUR) samples, respectively 46. Imputation was performed in each of the seven GWAS data sets individually and by using those SNPs that passed QC thresholds (mean call rate >0.95, MAF>0.01, HWE *P*>1 × 10^−6^ in controls). SNPs with impute information less than 80%, MAF less than 1% and HWE *P* in controls less than 1 × 10^−6^ were excluded from further analysis.

To determine whether specific coding variants within *HLA* genes contribute to the diverse association signals, we imputed classical *HLA* alleles and coding variants across the *HLA* region (chr6:20–40 Mb build 36) in each of the seven data sets. The five European data sets were imputed using a reference panel constructed using 2,767 individuals from the MHC Working Group of the Type 1 Diabetes Genetics Consortium (T1DGC)^47^. The two Chinese data sets were imputed using a reference panel constructed using genotypes from 89 Chinese (CHB) and Japanese (JPT) samples from the HapMap project^48^. Each reference panel was constructed using dense SNP genotype data and classical *HLA* alleles determined to four-digit resolution. Based on the EMBL-EBI Immunogenetics *HLA* Database, amino-acid variants were coded in as binary markers (present/absent) in the reference panel. Imputation of classical *HLA* alleles and their corresponding amino acids were performed using BEAGLE as previously described^47^,^49^. A total of 253 classical *HLA* alleles (two- and four-digit resolution) and 357 amino-acid positions were imputed in the European samples, and 167 classical alleles and 305 amino-acid positions were imputed in the Chinese samples.

### Genomic controls

We used PCA-based method to analyse and detect population stratification in individual GWAS data set. Principal component adjustments were performed in four data sets, respectively (one, one, two, three principal components (PCs) for Anhui, KI, UC and Kiel cohorts, respectively), to adjust for population stratification. This reduced the extent of genome-wide inflation to <1.05 in all individual GWAS study (Table 1), a level considered acceptable by conventional GWAS standard. The cases and controls of the Singapore data set were well matched, and no PCA-based correction was performed.

The final inflation factors of the test statistics were modest (*λ*_1,000_=1.017, 1.01 and 1.011 for the Caucasian, Chinese and combined discovery cohorts, respectively; Table 1), which suggested that the final association results from the three genome-wide analysis were largely free from major inflation because of population stratification.

### Association analysis

Genome-wide meta analyses (GWMA) were performed in this study: (i) Caucasian data (*N*=8,682, 4,778,154 SNPs); (ii) Han Chinese data (*N*=5,134, 4,562,294 SNPs); (iii) combined data (*N*=13,816, 3,621,551 SNPs).

The association analyses were done in the imputed dosage data using SNPtest v2 using score test, with the study and principal components (for four data sets) included as covariates to adjust for study effect and population stratification (Table 1). We also applied DerSimonian-Laid random effect model to detect ethnic heterogeneity by using odds ratio estimates and standard errors from GWMA of Caucasian and GWMA of Chinese data as the inputs. Association evidence from replication study was tested using an inverse variance meta-analysis assuming fixed-effects, with a Cochran's *Q* test and *I*^2^ to assess between-study heterogeneity. A *P*<5 × 10^−8^ was used as threshold for genome-wide significance.

In *HLA* region, association tests were performed on genotype dosages as determined from the imputed genotype probabilities, which would account for any uncertainties in the imputed genotypes. We conducted Wald tests on all the imputed bi-allelic variants, adjusting for study variables and principal components as covariates. For multiallelic amino-acid sites, we performed the global ‘omnibus' test at each site taking into account all alleles at each multiallelic position. The omnibus test was performed as previously described^50^, in summary: convert **k**-alleles to **k**-1 bi-alleles, invoke the glm function in R to estimate the multivariate model, and use the likelihood ratio test to compute the global multiallelic test *P*-values. All conditional logistic regression analyses were done using R.

### SNP selection and replication

We selected 43 SNPs with suggestive evidence of association (*P*<5 × 10^−5^ from the single variant logistic regression analysis) in at least one meta-analysis (Caucasian, Chinese and Combined) for the independent validation analysis. The Sequenom MassArray system was used for most of the replication studies, except for Celera samples, where 30 SNPs were genotyped using TaqMan assays (Life Technologies), and Han Chinese samples, where 12 SNPs were genotyped using TaqMan assays.

### Conditional analysis

To detect novel secondary association signals, we performed conditional analysis in 45 regions (41 known GWAS loci and 4 novel loci). Secondary SNPs with conditional *P*<5 × 10^−8^ was then assumed to be independent from the lead SNP in this region. The *P* values in multivariate analysis were reported after adjusting for the leading SNPs.

### Functional annotation and eQTL analysis

We annotated the four novel SNPs and their linked SNPs in 1000 Genomes Project Phase 1 CEU panel (*r*^2^>0.8) using HaploReg 2 (http://www.broadinstitute.org/mammals/haploreg/haploreg.php). We further performed their eQTL analyses using the following recently published eQTL data sets: (i) a meta-analysis of the transcriptional profiles from the peripheral blood cells of 5,311 Europeans (http://genenetwork.nl/bloodeqtlbrowser)^51^; (ii) the transcriptional profile from primary B cells and monocytes in 288 healthy Europeans^52^; (iii) the eqtl.uchicago browser with compiled data across diverse tissues.

### GO enrichment analysis

We submitted the 54 implicated genes (Supplementary Data 1) to the Database for Annotation, Visualization and Integrated Discovery (DAVID)^53^ for GO term enrichment analysis based on GO level. Fisher's exact test was implemented in DAVID to compute the enrichment *P* value for each GO term. We excluded the *HLA* region in this analysis. And we only reported the significant results at *P*<10^−4^.

### Ethnic heterogeneity

To compare the ethnic heterogeneity of susceptibility in psoriasis, we selected from the 44 non-MHC confirmed SNPs or proxy SNPs with considerate LD for validation mostly in our GWAS samples. Based on the comparisons between reference allele frequency and the association results, ethnic heterogeneity was categorized into three types: (i) loci with common variants, in which the same susceptible signals existed in Caucasian and Chinese Han; (ii) loci ethnic specific, in which there was significant association evidence only in one specific race (locus heterogeneity); (iii) Loci with allelic heterogeneity, in which the association in diverse populations showed obvious different causal variants.

### Geospatial risk analysis for 27 shared loci (30 SNPs) on HGDP

To assess the geospatial pattern of genetic risk, we used the publicly available genotype data from the HGDP with 1,043 individuals of 52 worldwide populations. The genotypes were generated on Illumina 650Y arrays for 660,918 markers (Stanford University). This high-quality genotype data (overall sample call rate >98.5%) was available for 10 out of 30 shared SNPs. The QC had been done before imputing the missing genotyped SNPs. Eighty-eight individuals were removed because of low heterozygosity rate and first-degree relatives and markers excluded with low call rate (<95%) and MAF (<0.01). Using the remaining data set with 955 individuals (52 populations) and 630,559 markers, the whole-genome imputation was performed using 1000 Genome Projects data set (Phase I, Dec. 2013 version) as reference panel. All 30 SNPs were imputed with high confidence (impute info 0.90 or above), and MAF bigger than 0.05.

For each individual (*j*), the genetic risk score of multiple locus (*m*) was estimated by


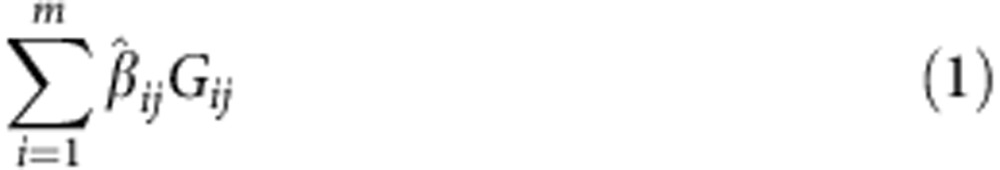


where 
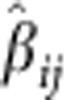
 was the log of adjusted meta-OR for each locus, *G*_*ij*_ was the number of risk alleles at each locus. The standardized risk score was calculated using a z-score method across populations by


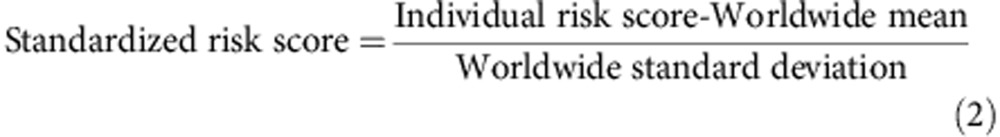


The median of standardized risk score was compared among 52 worldwide populations by Kruskal–Wallis test. The standardized risk score was correlated with longitude, latitude of the population.

### Odds ratio by risk score profile of ten population-specific loci

For this purpose, the polygenic risk score (PRS) for each individual was calculated as counts of risk alleles of ten population-specific loci^54^. The OR was estimated using logistic regression on PRS with adjustment of PCs to control for population stratification. There are seven cohorts (two for Chinese and five for Caucasian) in the discovery stage of this study. The adjusted ORs were estimated individually for seven cohorts. Meta-analysis was performed separately in Chinese and Caucasian using PLINK v1.07.

To analyse the ORs by risk score profile, the PRS was converted to deciles (1=lowest, 10=highest PRS). Nine dummy variables were created to compare 2–10 to decile 1 as the reference. ORs by deciles were estimated by logistic regression with PCs adjusted. The ORs were combined separately in Chinese and Caucasian using META v1.5 with fixed effects model. The 95% confidence intervals were calculated by *exp*(*BETA*±1.96*SE).

### Difference in prevalence between Chinese and Caucasian accounted for by ten population-specific loci

The method was modified based on Supplementary Methods published by William *et al*.^55^. We modelled the psoriasis prevalence accounted for by the ten Caucasian-specific loci in both Caucasian and Han Chinese and determined the amount of psoriasis prevalence would be reduced if the ten SNPs were absent from each population. The psoriasis prevalence was about ten times higher in Caucasian (2.5%) than in Chinese (0.2%). So we calculated estimates of reduction relative to the impact sum of the ten SNPs on Chinese prevalence. We applied a standard log-additive effect model and used population-based ORs from either Chinese (OR=1) or Caucasian (ORs from the current study) and assumed the OR was a good estimate of relative risk, *R*. We also assumed these ten loci contributed to the risk of psoriasis independently, and they exerted additive risk effect in total. Then, the overall psoriasis prevalence in population P was modelled as: 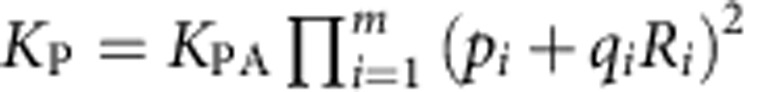, where *K*_PA_ was the disease prevalence if all loci were absent from the population, *m* was the number of loci, *p*_*i*_ was the frequency of non-risk allele for *i*th loci, *q*_*i*_ the frequency of risk allele for *i*th loci. We then calculated the proportion of prevalence in difference in Caucasian (CA) and Chinese (CH) accounted for by ten loci by


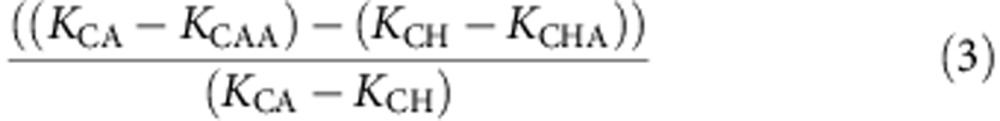


Where *K*_CA_ was the overall prevalence in Caucasian and *K*_CH_ the overall prevalence in Chinese.

### Co-expression-driven gene functional prediction

We used a described method to shed insight into the putative functions of the biological implicated genes in our study (http://genenetwork.nl:8080/GeneNetwork/)^56^. Gene function prediction is based on the idea that genes with shared expression profiles are likely to have related biological functions. The method uses data on co-expression profiles to predict the likely functions of as-of-yet uncharacterized genes and refine our understanding of the function of other genes. To apply the method, we queried the co-expression database with our four novel genes. The query for each gene returned the probable function of the gene or the reconstituted pathway in which it operates. The database was generated by linking information about gene expression obtained from published data on approximately 80,000 gene expression profiles (from the database Gene Expression Omnibus).

## Author contributions

J.J.L., X.J.Z., M. Seielstad, W.L., M. Ståhle and A.F. organized and designed the study. J.J.L., X.J.Z., M. Seielstad, W.L., M. Ståhle and A.F. conducted and supervised the genotyping of samples. X.Y.Y., H.Q.L., L.W., J.N.F, Y.L., P.D.B. and J.J.L. contributed to the design and execution of statistical analyses. X.Y.Y., H.Q.L., L.W. and J.J.L. wrote the first draft of the manuscript. Y.H.L., E.E., J.L.H., X.E., L.D.S., X.B.Z., C.B.S., C.H.Z., A.P.Z., F.S., L.P., J.J.C., G.G.K., K.P.C., S.M., M.W., S.W., W.L., K.S., Y.Y.T., C.T.S.T., R.G., A.B., P.L.D.J. and A.A.Q. contributed samples to the GWAS and/or follow-up genotyping. All authors contributed to the writing of the manuscript.

## Additional information

**How to cite this article:** Yin, X. *et al*. Genome-wide meta-analysis identifies multiple novel associations and ethnic heterogeneity of psoriasis susceptibility. *Nat. Commun*. 6:6916 doi: 10.1038/ncomms7916 (2015).

## Supplementary Material

Supplementary Information Supplementary Figures 1-8 and Supplementary Tables 1-11

Supplementary Data 1 Comparison of association results of 45 SNPs from 41 psoriasis susceptibility loci between European and Chinese population

Supplementary Data 2 Summary of the GWAS and replication results for the 43 top SNPs in GWAS meta-analysis

Supplementary Data 3 Conditional association results of secondary association in 6 loci that did not reach genome-wide significance

Supplementary Data 4 Conditional association results of IL23R gene region

## Figures and Tables

**Table 1:** Description of study samples.

Study	Ethnicity	Population sample	Cases (*N*)	Controls (N)	Total	*λ*_GC_	*λ*_GC___1000_
*GWAS data sets*							
UC	European	US	202	492	694	1.041*	1.143
KI	European	Sweden	727	1,198	1,925	1.032*	1.035
GAIN	European	US	1,336	1,372	2,708	1.032	1.024
Kiel	European	Germany	471	1,129	1,600	1.046*	1.069
Genizon	European	Canada	760	995	1,755	1.039	1.045
European			3,496	5,186	8,682	1.073†	1.017
Anhui	Chinese	China	1,139	1,112	2,251	1.016*	1.014
Singapore	Chinese	Singapore	449	2,434	2,883	1.031	1.041
Chinese			1,588	3,546	5,134	1.022†	1.01
TOTAL			5,084	8,732	13,816	1.071†	1.011
							
*Replication cohorts*							
UC	European	US	896	636	1,532	—	—
KI	European	Sweden	1,025	510	1,535	—	—
Harvard	European	US	559	572	1,131	—	—
Spain	European	Spain	408	327	735	—	—
Celera	European	US	1,436	1,380	2,816	—	—
Kiel	European	Germany	810	2,208	3,018	—	—
Anhui	Chinese	China	5,151	5,152	10,303	—	—
TOTAL			10,285	10,785	21,070	—	

^*^Adjust for principal components (PCs).

^†^Adjust for studies and PCs.

**Table 2:** Meta-analysis results of the 4 novel psoriasis susceptibility loci.

Gene	SNP	Chr	BP.B37	RA	RAF		Stage	European		Chinese		Combined All		
					EUR	CHN		*P*	OR(95%CI)	*P*	OR(95%CI)	*P*	OR(95%CI)	*P*_Q_
TP63	rs28512356	3	189615475	C	0.799	0.826	GWAS	9.99E−06	1.20 (1.11–1.30)	9.52E−01	1.00 (0.88–1.13)	7.70E−06	1.10 (1.06–1.15)	9.62E−04
							Validation	9.30E−04	1.15 (1.06–1.25)	4.93E−01	1.03 (0.96–1.10)			
							Combined	4.31E−08	1.17 (1.11–1.24)	5.68E−01	1.02 (0.96–1.08)			
COG6	rs34394770	13	40333369	T	0.643	0.763	GWAS	5.55E−07	1.18 (1.11–1.26)	1.22E−01	1.10 (0.98–1.23)	4.87E−08	1.11 (1.07–1.15)	2.04E−02
							Validation	8.21E−03	1.12 (1.03–1.21)	1.43E−01	1.05 (0.98–1.11)			
							Combined	2.65E−08	1.16 (1.10–1.22)	4.63E−02	1.06 (1.00–1.12)			
LOC144817	rs9533962	13	45334194	C	0.406	0.449	GWAS	1.76E−03	1.11 (1.04–1.18)	3.93E−05	1.23 (1.12–1.36)	7.53E−11	1.12 (1.08–1.15)	2.91E−01
							Validation	1.60E−06	1.16 (1.09–1.24)	5.30E−02	1.06 (1.00–1.12)			
							Combined	1.93E−08	1.14 (1.09–1.19)	2.25E−04	1.09 (1.04–1.15)			
RUNX1	rs8128234	21	36470865	T	0.197	0.111	GWAS	1.82E−05	1.19 (1.10–1.28)	8.98E−02	1.15 (0.98–1.34)	5.99E−08	1.13 (1.08–1.18)	4.23E−02
							Validation	4.77E−04	1.15 (1.06–1.24)	3.35E−01	1.04 (0.96–1.13)			
							Combined	3.74E−08	1.17 (1.11–1.23)	9.88E−02	1.06 (0.99–1.14)			

BP.B37, position based on NCBI build 37; CHN, Han Chinese samples; Chr, chromosome; GWAS, genome-wide association study; EUR, European samples; RA, risk alleles; RAF, risk allele's frequencies; OR, odds ratio; *P*: association *P* values in logistic regression test (alleles dosages in GWAS studies); 95% CI, 95% confidence interval; *P*_Q_: *P* values of heterogeneity test; SNP, single-nucleotide polymorphism.

## References

[b1] Nestle F. O., Kaplan D. H. & Barker J. Psoriasis. N. Engl. J. Med. 361, 496–509 (2009). 19641206 10.1056/NEJMra0804595

[b2] Chandran V. & Raychaudhuri S. P. Geoepidemiology and environmental factors of psoriasis and psoriatic arthritis. J. Autoimmun. 34, J314–J321 (2010). 20034760 10.1016/j.jaut.2009.12.001

[b3] Ding X. . Prevalence of psoriasis in China: a population-based study in six cities. Eur. J. Dermatol. 22, 663–667 (2012). 22910173 10.1684/ejd.2012.1802

[b4] Gelfand J. M. . The prevalence of psoriasis in African Americans: results from a population-based study. J. Am. Acad. Dermatol. 52, 23–26 (2005). 15627076 10.1016/j.jaad.2004.07.045

[b5] Christophers E. Explaining phenotype heterogeneity in patients with psoriasis. Br. J. Dermatol. 158, 437–441 (2008). 18047519 10.1111/j.1365-2133.2007.08307.x

[b6] Heredia E. E. . Heterogeneity of response to biologic treatment: perspective for psoriasis. J. Invest. Dermatol. (2013). 10.1038/jid.2013.326 23921949

[b7] Zhang X., Wang H., Te-Shao H., Yang S. & Chen S. The genetic epidemiology of psoriasis vulgaris in Chinese Han. Int. J. Dermatol. 41, 663–669 (2002). 12390189 10.1046/j.1365-4362.2002.01596.x

[b8] de Cid R. . Deletion of the late cornified envelope LCE3B and LCE3C genes as a susceptibility factor for psoriasis. Nature Genet. 41, 211–215 (2009). 19169253 10.1038/ng.313 PMC3128734

[b9] Ellinghaus E. . Genome-wide association study identifies a psoriasis susceptibility locus at TRAF3IP2. Nature Genet. 42, 991–995 (2010). 20953188 10.1038/ng.689 PMC3136364

[b10] Huffmeier U. . Common variants at TRAF3IP2 are associated with susceptibility to psoriatic arthritis and psoriasis. Nature Genet. 42, 996–999 (2010). 20953186 10.1038/ng.688 PMC2981079

[b11] Nair R. P. . Genome-wide scan reveals association of psoriasis with IL-23 and NF-kappaB pathways. Nature Genet. 41, 199–204 (2009). 19169254 10.1038/ng.311 PMC2745122

[b12] Strange A. . A genome-wide association study identifies new psoriasis susceptibility loci and an interaction between HLA-C and ERAP1. Nature Genet. 42, 985–990 (2010). 20953190 10.1038/ng.694 PMC3749730

[b13] Stuart P. E. . Genome-wide association analysis identifies three psoriasis susceptibility loci. Nature Genet. 42, 1000–1004 (2010). 20953189 10.1038/ng.693 PMC2965799

[b14] Sun L. D. . Association analyses identify six new psoriasis susceptibility loci in the Chinese population. Nature Genet. 42, 1005–1009 (2010). 20953187 10.1038/ng.690 PMC3140436

[b15] Zhang X. J. . Psoriasis genome-wide association study identifies susceptibility variants within LCE gene cluster at 1q21. Nature Genet. 41, 205–210 (2009). 19169255 10.1038/ng.310

[b16] Tsoi L. C. . Identification of 15 new psoriasis susceptibility loci highlights the role of innate immunity. Nature Genet. 44, 1341–1348 (2012). 23143594 10.1038/ng.2467 PMC3510312

[b17] Yin X. . Common variants explain a large fraction of the variability in the liability to psoriasis in a Han Chinese population. BMC Genomics 15, 87 (2014). 24479639 10.1186/1471-2164-15-87 PMC3909441

[b18] Ripke S. . Genome-wide association analysis identifies 13 new risk loci for schizophrenia. Nature Genet. 45, 1150–1159 (2013). 23974872 10.1038/ng.2742 PMC3827979

[b19] Bonnelykke K. . Meta-analysis of genome-wide association studies identifies ten loci influencing allergic sensitization. Nature Genet. 45, 902–906 (2013). 23817571 10.1038/ng.2694 PMC4922420

[b20] Berndt S. I. . Genome-wide association study identifies multiple risk loci for chronic lymphocytic leukemia. Nature Genet. 45, 868–876 (2013). 23770605 10.1038/ng.2652 PMC3729927

[b21] Anttila V. . Genome-wide meta-analysis identifies new susceptibility loci for migraine. Nature Genet. 45, 912–917 (2013). 23793025 10.1038/ng.2676 PMC4041123

[b22] Wu Y. . Trans-ethnic fine-mapping of lipid loci identifies population-specific signals and allelic heterogeneity that increases the trait variance explained. PLoS Genet. 9, e1003379 (2013). 23555291 10.1371/journal.pgen.1003379 PMC3605054

[b23] Marigorta U. M. & Navarro A. High trans-ethnic replicability of GWAS results implies common causal variants. PLoS Genet. 9, e1003566 (2013). 23785302 10.1371/journal.pgen.1003566 PMC3681663

[b24] Jostins L. . Host-microbe interactions have shaped the genetic architecture of inflammatory bowel disease. Nature 491, 119–124 (2012). 23128233 10.1038/nature11582 PMC3491803

[b25] Pharoah P. D. . GWAS meta-analysis and replication identifies three new susceptibility loci for ovarian cancer. Nature Genet. 45, 362–370 370e1-2 (2013). 23535730 10.1038/ng.2564 PMC3693183

[b26] Nyholt D. R. . Genome-wide association meta-analysis identifies new endometriosis risk loci. Nature Genet. 44, 1355–1359 (2012). 23104006 10.1038/ng.2445 PMC3527416

[b27] Romano R. A. . DeltaNp63 knockout mice reveal its indispensable role as a master regulator of epithelial development and differentiation. Development 139, 772–782 (2012). 22274697 10.1242/dev.071191 PMC3265062

[b28] Boris Fischer . p53 and TAp63 promote keratinocyte proliferation and differentiation in breeding tubercles of the Zebrafish. PLoS Genet. 10, e1004048 (2014). 24415949 10.1371/journal.pgen.1004048 PMC3886889

[b29] Ellinghaus E. . Identification of germline susceptibility loci in ETV6-RUNX1-rearranged childhood acute lymphoblastic leukemia. Leukemia 26, 902–909 (2012). 22076464 10.1038/leu.2011.302 PMC3356560

[b30] Miki D. . Variation in TP63 is associated with lung adenocarcinoma susceptibility in Japanese and Korean populations. Nature Genet. 42, 893–896 (2010). 20871597 10.1038/ng.667

[b31] Rothman N. . A multi-stage genome-wide association study of bladder cancer identifies multiple susceptibility loci. Nature Genet. 42, 978–984 (2010). 20972438 10.1038/ng.687 PMC3049891

[b32] Shiraishi K. . A genome-wide association study identifies two new susceptibility loci for lung adenocarcinoma in the Japanese population. Nature Genet. 44, 900–903 (2012). 22797724 10.1038/ng.2353

[b33] Du J. . Epidermal overexpression of transgenic DeltaNp63 promotes type 2 immune and myeloid inflammatory responses and hyperplasia via NF-kappaB activation. J. Pathol. 232, 356–368 (2014). 24258200 10.1002/path.4302

[b34] Scheitz C. J. & Tumbar T. New insights into the role of Runx1 in epithelial stem cell biology and pathology. J. Cell Biochem. 114, 985–993 (2013). 23150456 10.1002/jcb.24453 PMC5788165

[b35] Masse I. . Functional interplay between p63 and p53 controls RUNX1 function in the transition from proliferation to differentiation in human keratinocytes. Cell Death Dis. 3, e318 (2012). 22673192 10.1038/cddis.2012.62 PMC3388234

[b36] Wong W. F., Kohu K., Chiba T., Sato T. & Satake M. Interplay of transcription factors in T-cell differentiation and function: the role of Runx. Immunology 132, 157–164 (2011). 21091910 10.1111/j.1365-2567.2010.03381.x PMC3050439

[b37] Helms C. . A putative RUNX1 binding site variant between SLC9A3R1 and NAT9 is associated with susceptibility to psoriasis. Nature Genet. 35, 349–356 (2003). 14608357 10.1038/ng1268

[b38] Okada Y. . Genetics of rheumatoid arthritis contributes to biology and drug discovery. Nature 506, 376–381 (2014). 24390342 10.1038/nature12873 PMC3944098

[b39] Ota T. . Complete sequencing and characterization of 21,243 full-length human cDNAs. Nature Genet. 36, 40–45 (2004). 14702039 10.1038/ng1285

[b40] Laufman O., Hong W. & Lev S. The COG complex interacts directly with Syntaxin 6 and positively regulates endosome-to-TGN retrograde transport. J. Cell Biol. 194, 459–472 (2011). 21807881 10.1083/jcb.201102045 PMC3153647

[b41] Liu Y. . A genome-wide association study of psoriasis and psoriatic arthritis identifies new disease loci. PLoS Genet. 4, e1000041 (2008). 18369459 10.1371/journal.pgen.1000041 PMC2274885

[b42] Okada Y. . Fine mapping major histocompatibility complex associations in psoriasis and its clinical subtypes. Am. J. Hum. Genet. 95, 162–172 (2014). 25087609 10.1016/j.ajhg.2014.07.002 PMC4129407

[b43] Hughes A. L. & Yeager M. Natural selection at major histocompatibility complex loci of vertebrates. Annu. Rev. Genet. 32, 415–435 (1998). 9928486 10.1146/annurev.genet.32.1.415

[b44] Price A. L. . Principal components analysis corrects for stratification in genome-wide association studies. Nature Genet. 38, 904–909 (2006). 16862161 10.1038/ng1847

[b45] Howie B., Fuchsberger C., Stephens M., Marchini J. & Abecasis G. R. Fast and accurate genotype imputation in genome-wide association studies through pre-phasing. Nature Genet. 44, 955–959 (2012). 22820512 10.1038/ng.2354 PMC3696580

[b46] Frazer K. A. . A second generation human haplotype map of over 3.1 million SNPs. Nature 449, 851–861 (2007). 17943122 10.1038/nature06258 PMC2689609

[b47] Pereyra F. . The major genetic determinants of HIV-1 control affect HLA class I peptide presentation. Science 330, 1551–1557 (2010). 21051598 10.1126/science.1195271 PMC3235490

[b48] de Bakker P. I. . A high-resolution HLA and SNP haplotype map for disease association studies in the extended human MHC. Nature Genet. 38, 1166–1172 (2006). 16998491 10.1038/ng1885 PMC2670196

[b49] Raychaudhuri S. . Five amino acids in three HLA proteins explain most of the association between MHC and seropositive rheumatoid arthritis. Nature Genet. 44, 291–296 (2012). 22286218 10.1038/ng.1076 PMC3288335

[b50] Foo J. N. . Coding variants at hexa-allelic amino acid 13 of HLA-DRB1 explain independent SNP associations with follicular lymphoma risk. Am. J. Hum. Genet. 93, 167–172 (2013). 23791106 10.1016/j.ajhg.2013.05.020 PMC3710749

[b51] Westra H. J. . Systematic identification of trans eQTLs as putative drivers of known disease associations. Nature Genet. 45, 1238–1243 (2013). 24013639 10.1038/ng.2756 PMC3991562

[b52] Fairfax B. P. . Genetics of gene expression in primary immune cells identifies cell type-specific master regulators and roles of HLA alleles. Nature Genet. 44, 502–510 (2012). 22446964 10.1038/ng.2205 PMC3437404

[b53] Huang da W., Sherman B. T. & Lempicki R. A. Systematic and integrative analysis of large gene lists using DAVID bioinformatics resources. Nat. Protoc. 4, 44–57 (2009). 19131956 10.1038/nprot.2008.211

[b54] Schizophrenia Working Group of the Psychiatric Genomics Consortium. Biological insights from 108 schizophrenia-associated genetic loci. Nature 511, 421–427 (2014). 25056061 10.1038/nature13595 PMC4112379

[b55] Williams A. L. . Sequence variants in SLC16A11 are a common risk factor for type 2 diabetes in Mexico. Nature 506, 97–101 (2014). 24390345 10.1038/nature12828 PMC4127086

[b56] Cvejic A. . SMIM1 underlies the Vel blood group and influences red blood cell traits. Nature Genet. 45, 542–545 (2013). 23563608 10.1038/ng.2603 PMC4179282

