# Supplementary material for: Genome-wide meta-analysis identifies multiple novel associations and ethnic heterogeneity of psoriasis susceptibility
Source: Nat Commun. 2015 Apr 23;6:6916. doi: 10.1038/ncomms7916 (PMC4423213; doi:10.1038/ncomms7916)
Supplement: Supplementary Information — Supplementary Figures 1-8 and Supplementary Tables 1-11 [file ncomms7916-s1.pdf]

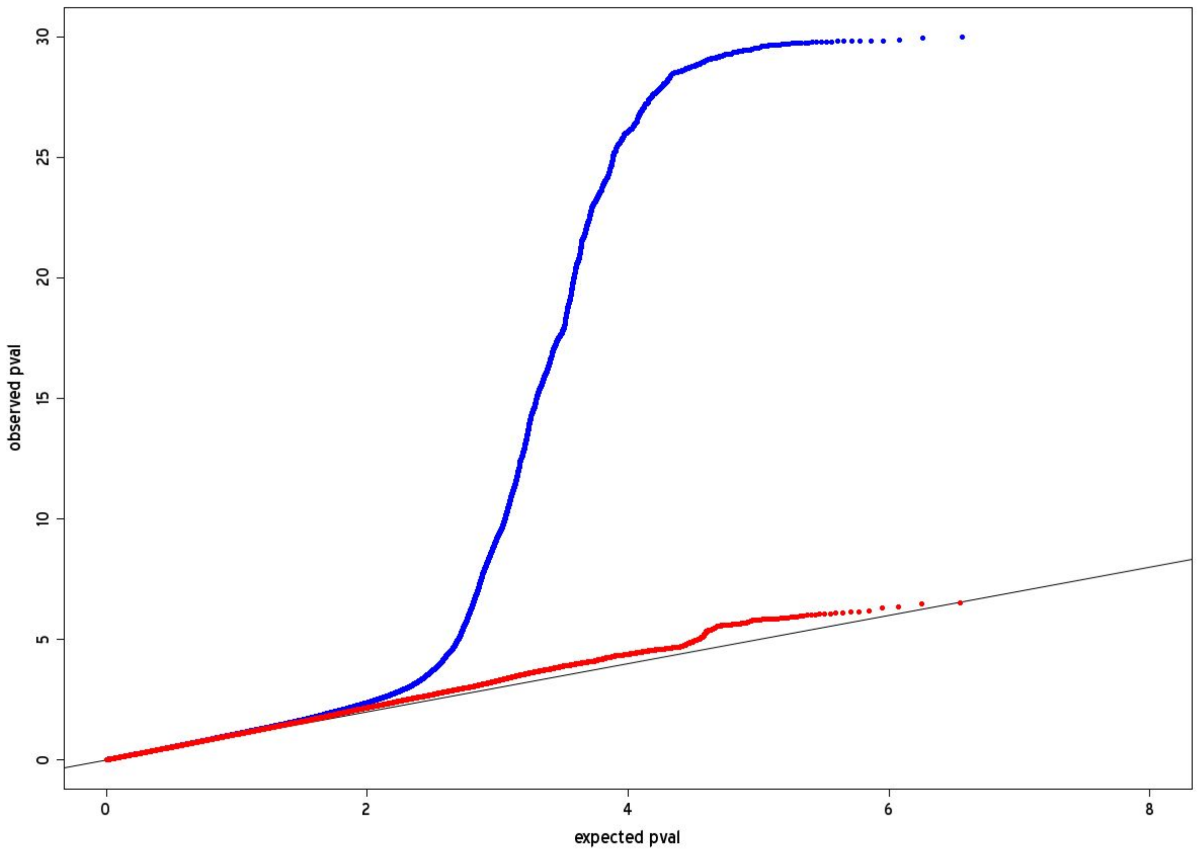

a. trans-ethnic GWAS meta-analysis

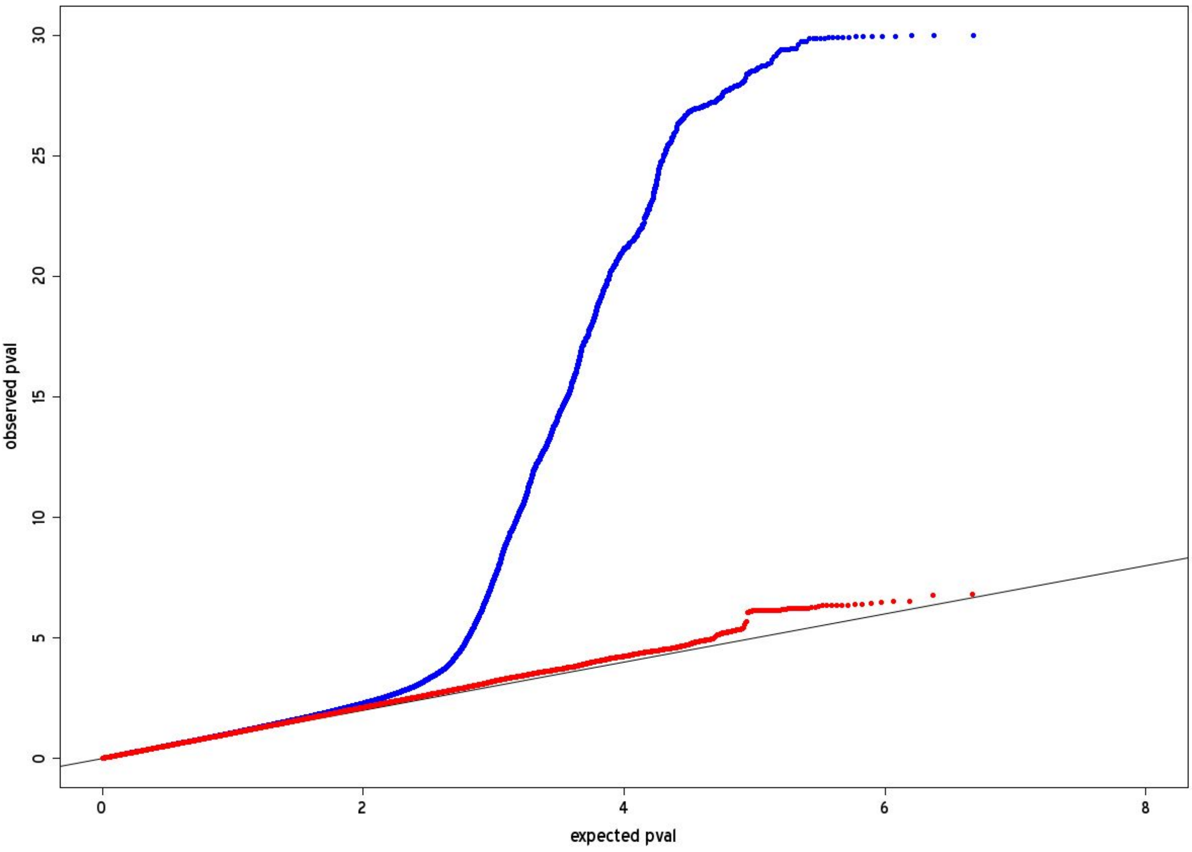

b. Caucasian GWAS meta-analysis

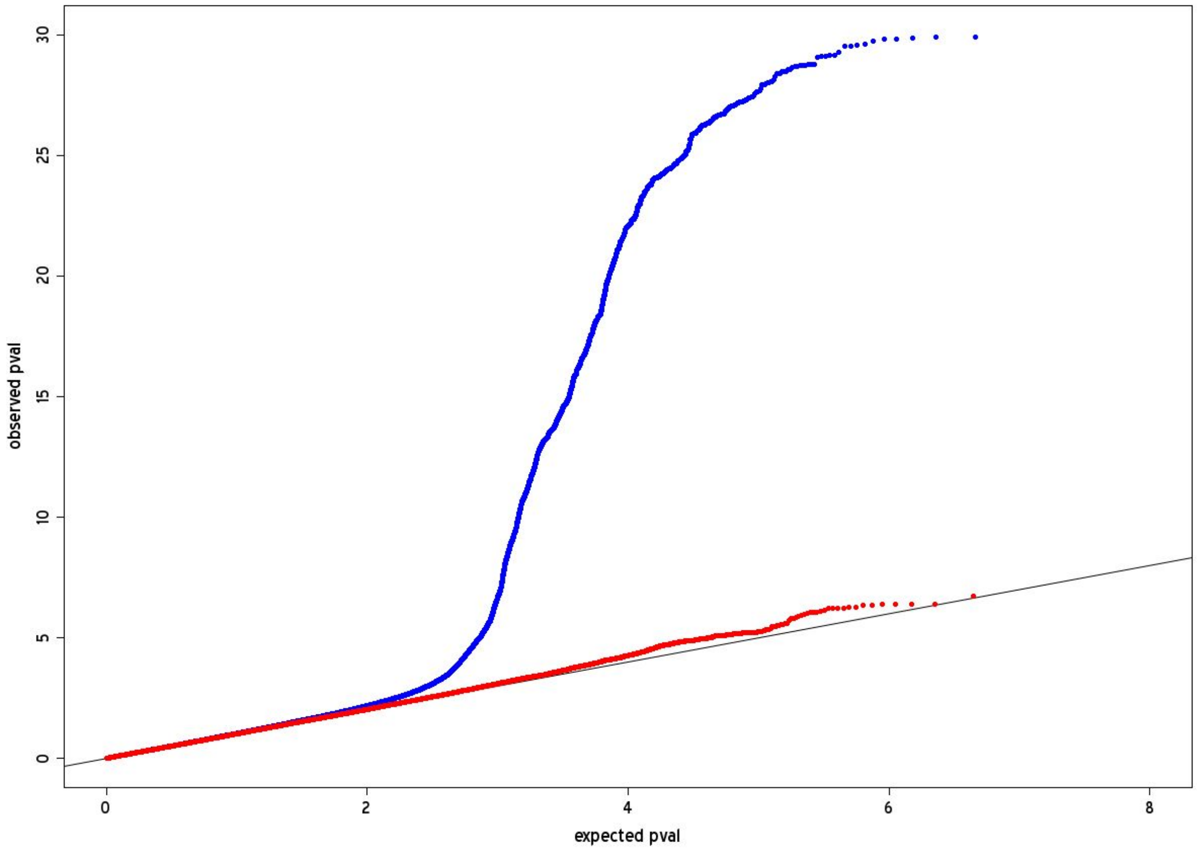

c. Chinese GWAS meta-analysis

Supplementary Figure 1: Quantile-quantile plot of single SNP test. Quantile-quantile plots of P values in the (a) trans-ethnic GWAS meta-analysis; (b) Caucasian GWAS meta-analysis; (c) Chinese GWAS meta-analysis. Blue dots indicate whole genome wide statistic; red dots indicate SNPs outside the 41 known psoriasis risk loci; gray line represents expected under null hypothesis.

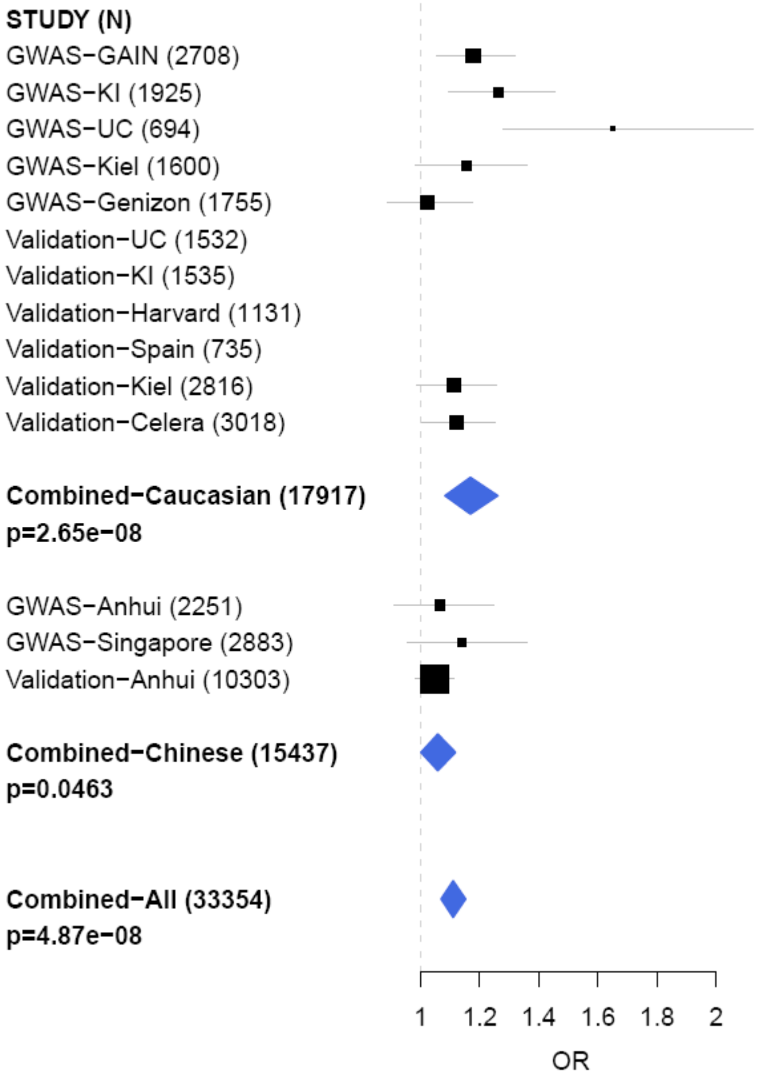
STUDY (N)
GWAS−GAIN (2708)
GWAS−KI (1925)
GWAS−UC (694)
GWAS−Kiel (1600)
GWAS−Genizon (1755)
Validation−UC (1532)
Validation−KI (1535)
Validation−Harvard (1131)
Validation−Spain (735)
Validation−Kiel (2816)
Validation−Celera (3018)
Combined−Caucasian (17917)
p=2.65e−08
GWAS−Anhui (2251)
GWAS−Singapore (2883)
Validation−Anhui (10303)
Combined−Chinese (15437)
p=0.0463
Combined−All (33354)
p=4.87e−08
1
1.2
1.4
1.6
1.8
2
OR

a. COG6

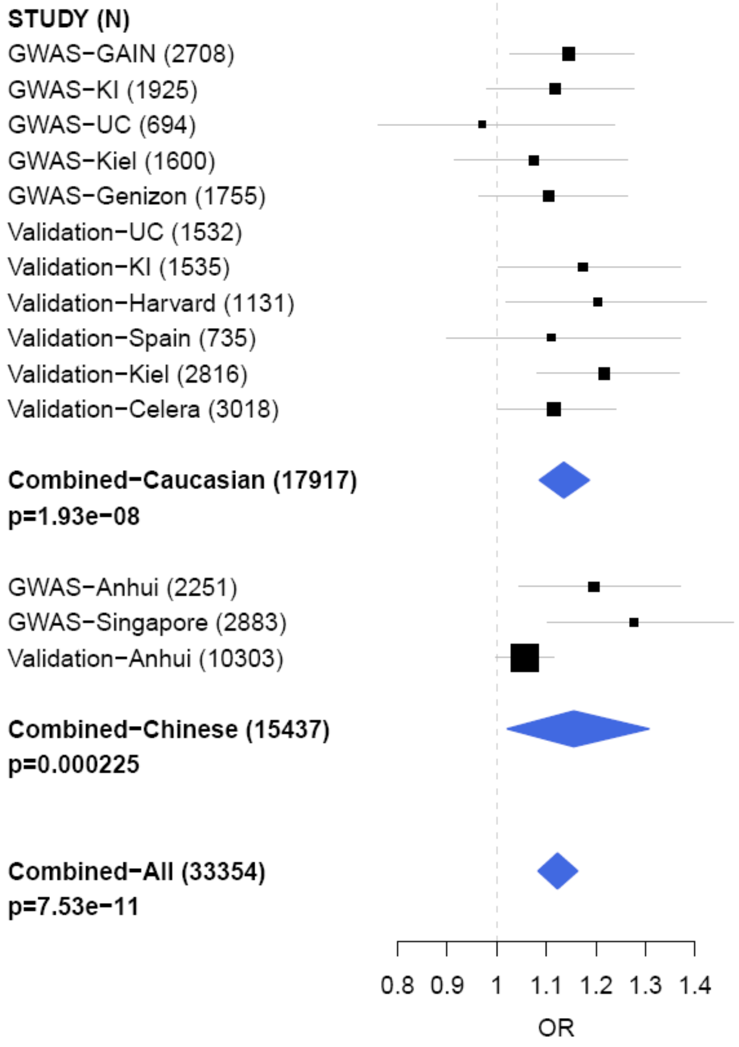
STUDY (N)
GWAS−GAIN (2708)
GWAS−KI (1925)
GWAS−UC (694)
GWAS−Kiel (1600)
GWAS−Genizon (1755)
Validation−UC (1532)
Validation−KI (1535)
Validation−Harvard (1131)
Validation−Spain (735)
Validation−Kiel (2816)
Validation−Celera (3018)
Combined−Caucasian (17917)
p=1.93e−08
GWAS−Anhui (2251)
GWAS−Singapore (2883)
Validation−Anhui (10303)
Combined−Chinese (15437)
p=0.000225
Combined−All (33354)
p=7.53e−11
0.8
0.9
1
1.1
1.2
1.3
1.4
OR

b. LOC144817

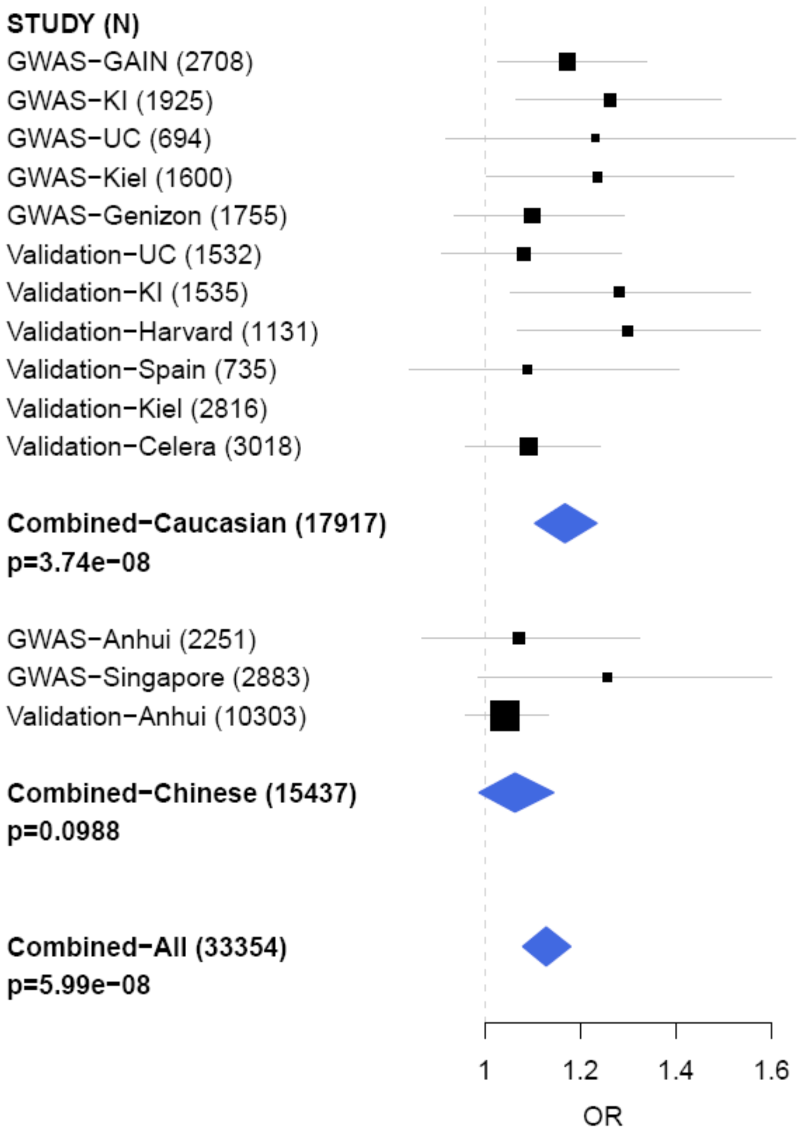

STUDY (N)
GWAS−GAIN (2708)
GWAS−KI (1925)
GWAS−UC (694)
GWAS−Kiel (1600)
GWAS−Genizon (1755)
Validation−UC (1532)
Validation−KI (1535)
Validation−Harvard (1131)
Validation−Spain (735)
Validation−Kiel (2816)
Validation−Celera (3018)
Combined−Caucasian (17917)
p=3.74e−08
GWAS−Anhui (2251)
GWAS−Singapore (2883)
Validation−Anhui (10303)
Combined−Chinese (15437)
p=0.0988
Combined−All (33354)
p=5.99e−08
1
1.2
1.4
1.6
OR

c. RUNX1

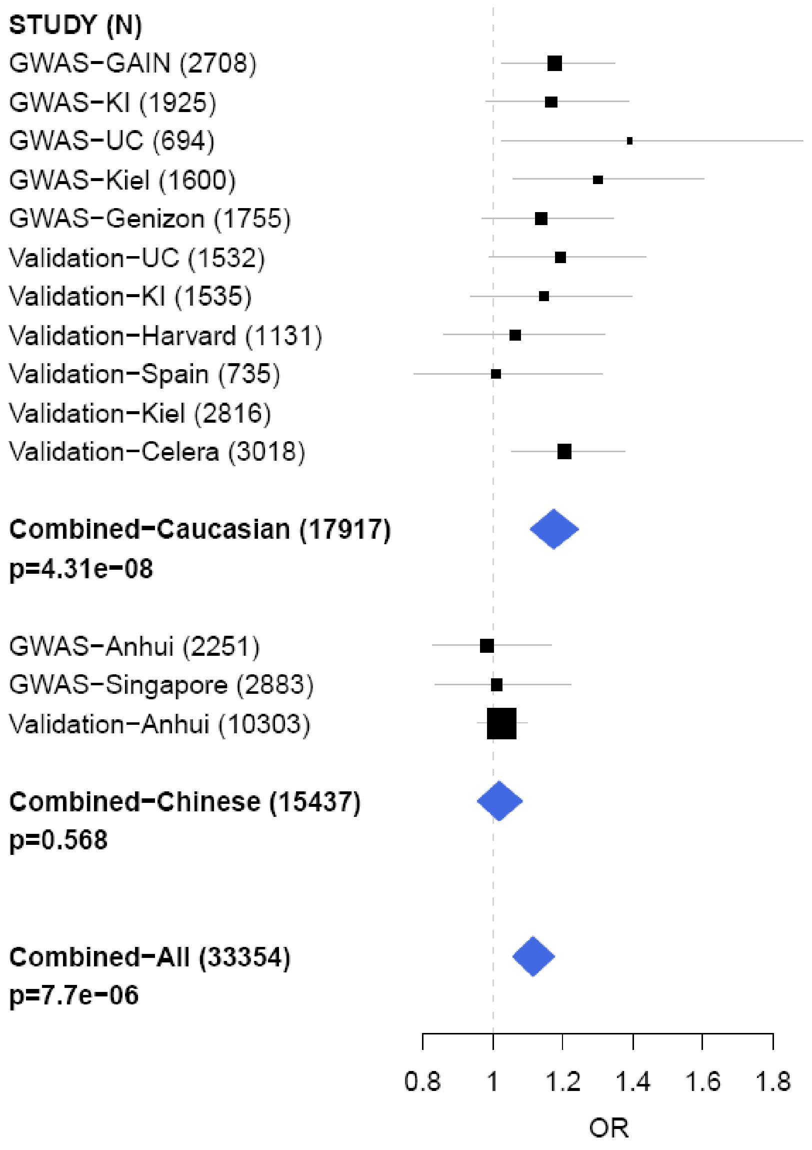

d. TP63

Supplementary Figure 2: The forest plot of four novel psoriasis risk SNPs. (a) rs34394770 in COG6 gene; (b) rs9533962 in LOC144817 gene; (c) rs8128234 in RUNX1 gene; (d) rs28512356 in TP63 gene. In the left, it indicates each study. The Plots show the study-specific association estimates (odds ratios) and 95% confidence intervals for the discovery and second-stage studies presented as bars. The association estimate and confidence interval for the meta-analysis combining the discovery and second-stage results are shown as a blue diamond. Blank spaces indicate occasions in which a particular study was not able to provide results for a given SNP.

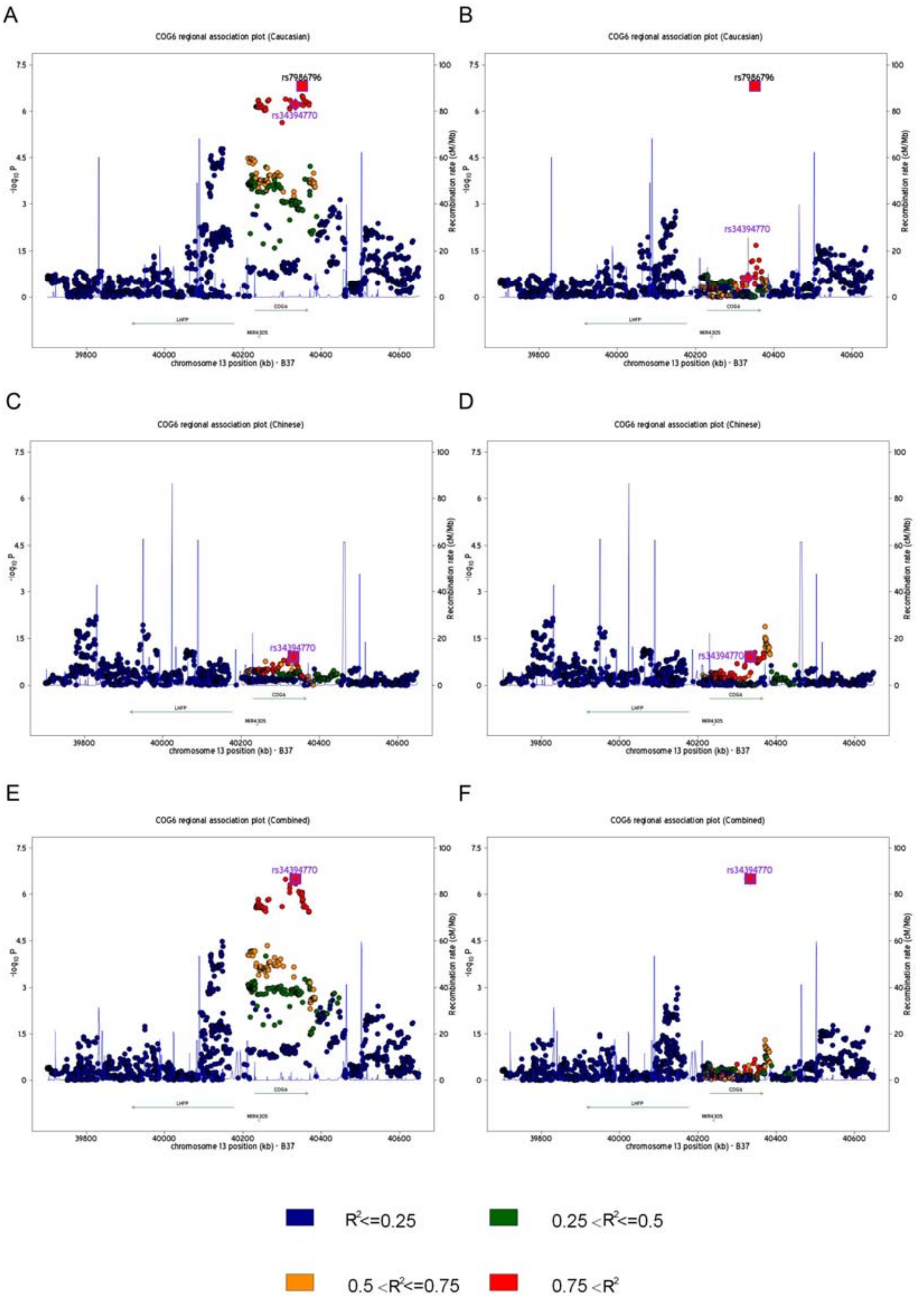
A
COG6 regional association plot (Caucasian)
rs7986796
rs34394770
B
COG6 regional association plot (Caucasian)
rs7986796
rs34394770
C
COG6 regional association plot (Chinese)
rs34394770
D
COG6 regional association plot (Chinese)
rs34394770
E
COG6 regional association plot (Combined)
rs34394770
F
COG6 regional association plot (Combined)
rs34394770
-log10 P
Recombination rate (cM/Mb)
chromosome 13 position (kb) - B37
LHFP
COG6
MIR4305
R2<=0.25
0.25 <R2<=0.5
0.5 <R2<=0.75
0.75 <R2

a. COG6

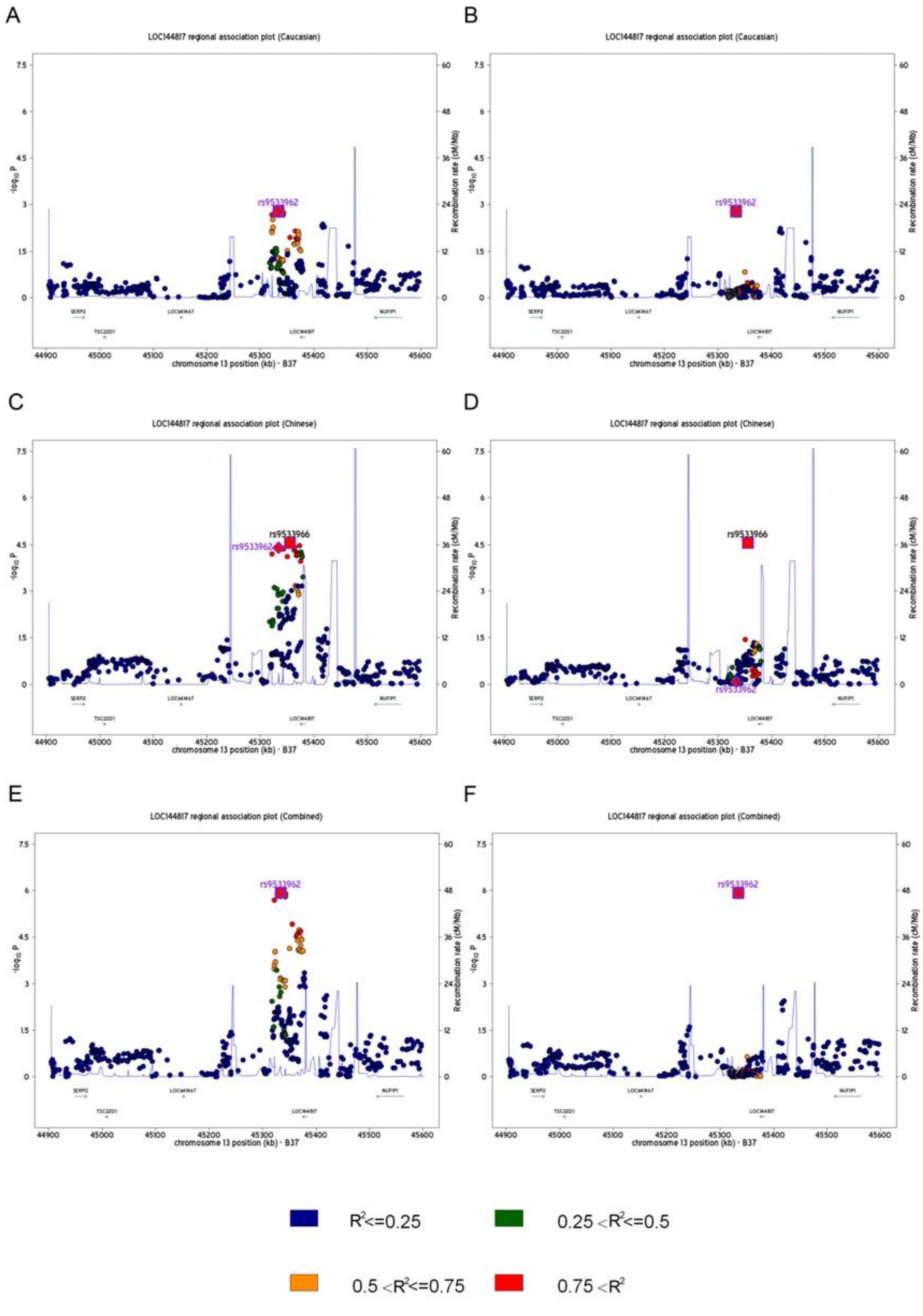
A
LOC144817 regional association plot (Caucasian)
B
LOC144817 regional association plot (Caucasian)
C
LOC144817 regional association plot (Chinese)
D
LOC144817 regional association plot (Chinese)
E
LOC144817 regional association plot (Combined)
F
LOC144817 regional association plot (Combined)
rs9533962
rs9533966
-log10 P
Recombination rate (cM/Mb)
chromosome 13 position (kb) - B37
SERP2
TSC22D1
LOC144817
NUFIP1
R²<=0.25
0.25 < R²<=0.5
0.5 < R²<=0.75
0.75 < R²

b. LOC144817

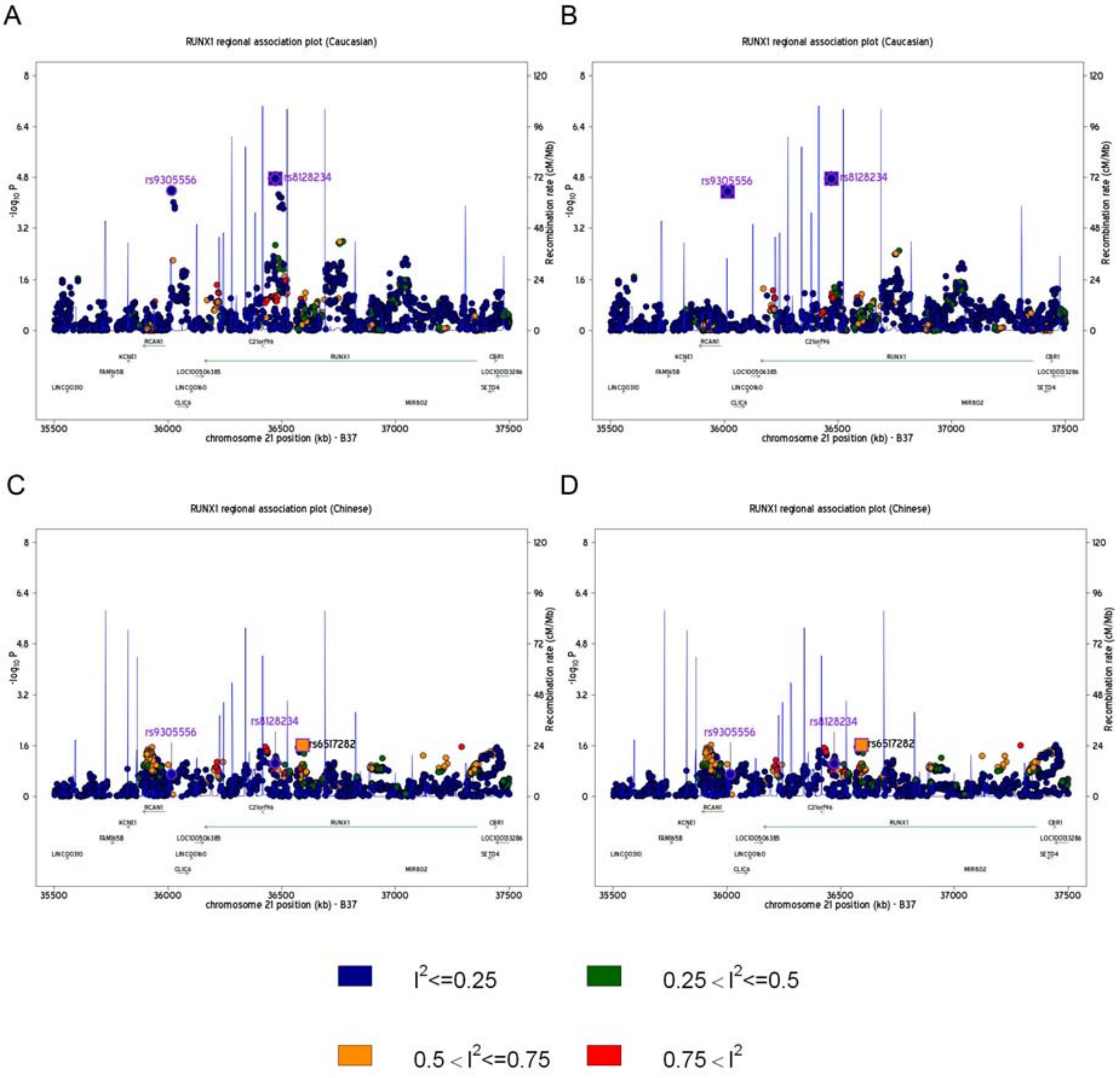
A
RUNX1 regional association plot (Caucasian)
rs9305556
rs8128234
B
RUNX1 regional association plot (Caucasian)
rs9305556
rs8128234
C
RUNX1 regional association plot (Chinese)
rs9305556
rs8128234
rs6517282
D
RUNX1 regional association plot (Chinese)
rs9305556
rs8128234
rs6517282
-log10 P
Recombination rate (cM/Mb)
chromosome 21 position (kb) - B37
RCAN1
KCNE1
FAM165B
LINC00310
LOC100506385
LINC00160
CLIC6
C21orf96
RUNX1
MIR802
CBR1
LOC100133286
SETD4
35500
36000
36500
37000
37500
I²<=0.25
0.25 < I²<=0.5
0.5 < I²<=0.75
0.75 < I²

c. RUNX1

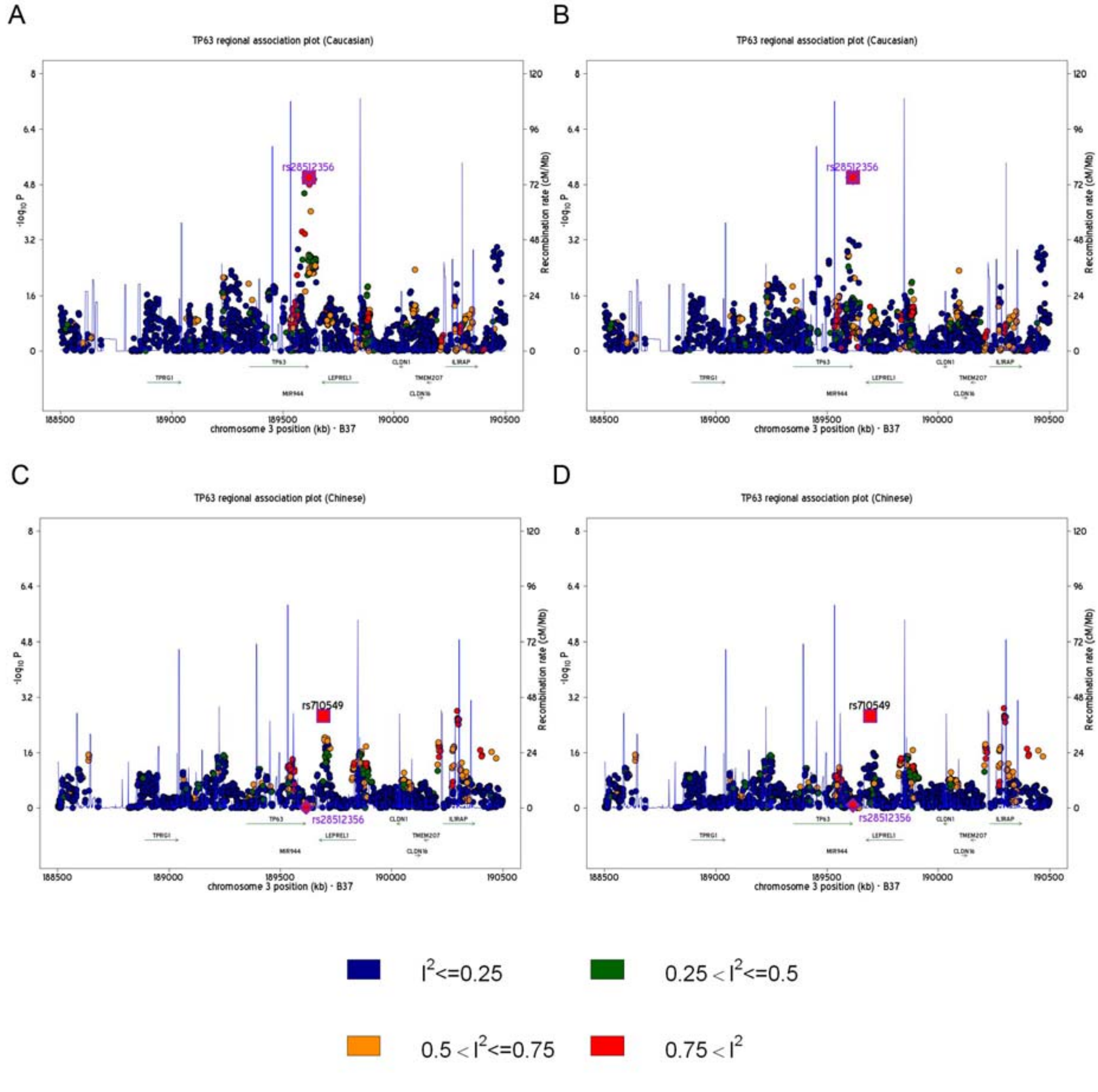
A
TP63 regional association plot (Caucasian)
rs28512356
B
TP63 regional association plot (Caucasian)
rs28512356
C
TP63 regional association plot (Chinese)
rs710549
rs28512356
D
TP63 regional association plot (Chinese)
rs710549
rs28512356
-log10 P
Recombination rate (cM/Mb)
chromosome 3 position (kb) - B37
TPRG1
TP63
MIR944
LEPREL1
CLDN1
TMEM207
CLDN16
IL1RAP
I2<=0.25
0.25 < I2<=0.5
0.5 < I2<=0.75
0.75 < I2

d. TP63

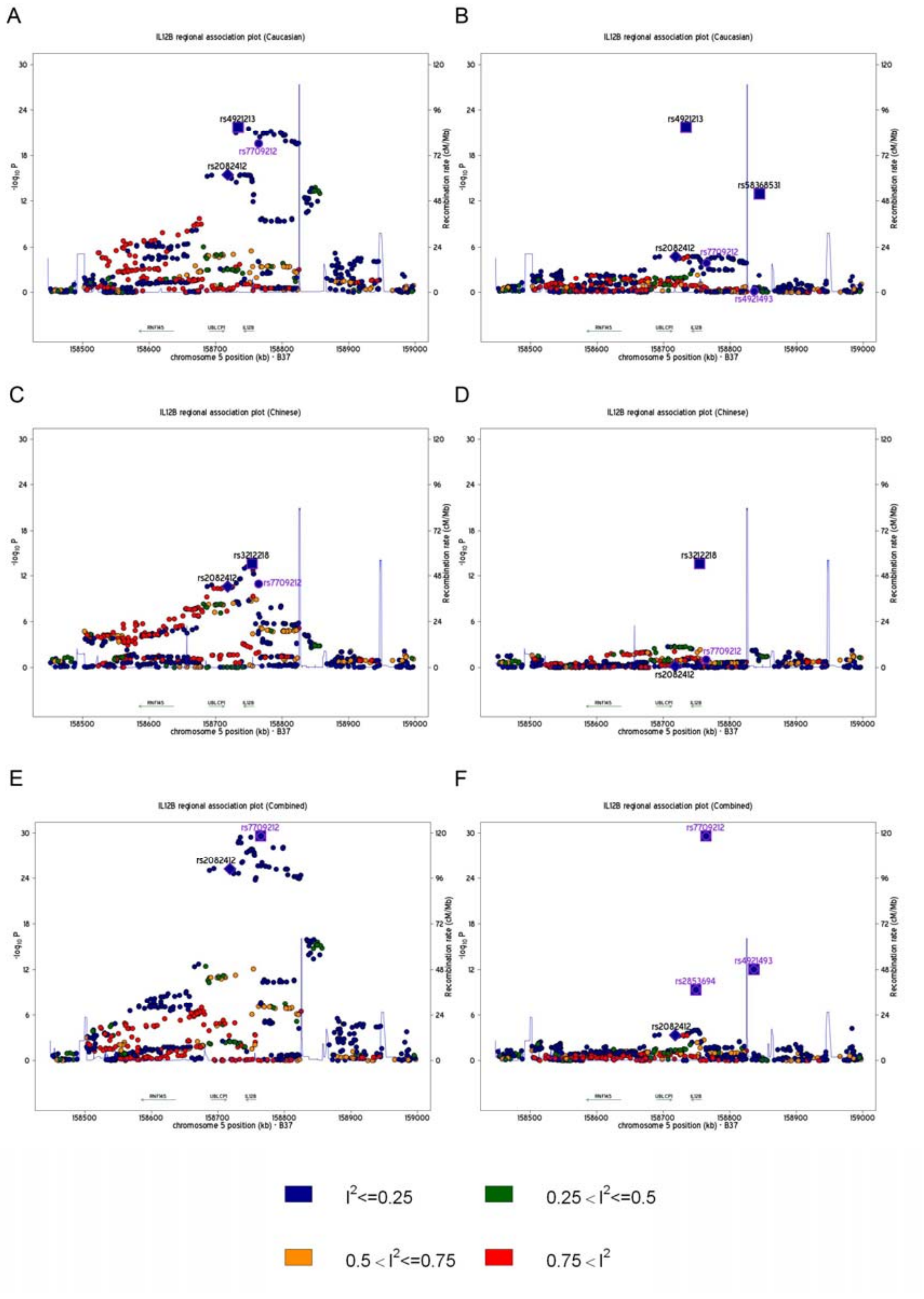
A
IL12B regional association plot (Caucasian)
rs4921213
rs7709212
rs2082412
B
IL12B regional association plot (Caucasian)
rs4921213
rs58368531
rs2082412
rs7709212
rs4921493
C
IL12B regional association plot (Chinese)
rs3212218
rs2082412
rs7709212
D
IL12B regional association plot (Chinese)
rs3212218
rs7709212
rs2082412
E
IL12B regional association plot (Combined)
rs7709212
rs2082412
F
IL12B regional association plot (Combined)
rs7709212
rs4921493
rs2853694
rs2082412
chromosome 5 position (kb) - B37
Recombination rate (cM/Mb)
I2<=0.25
0.25 < I2<=0.5
0.5 < I2<=0.75
0.75 < I2

e. IL12B

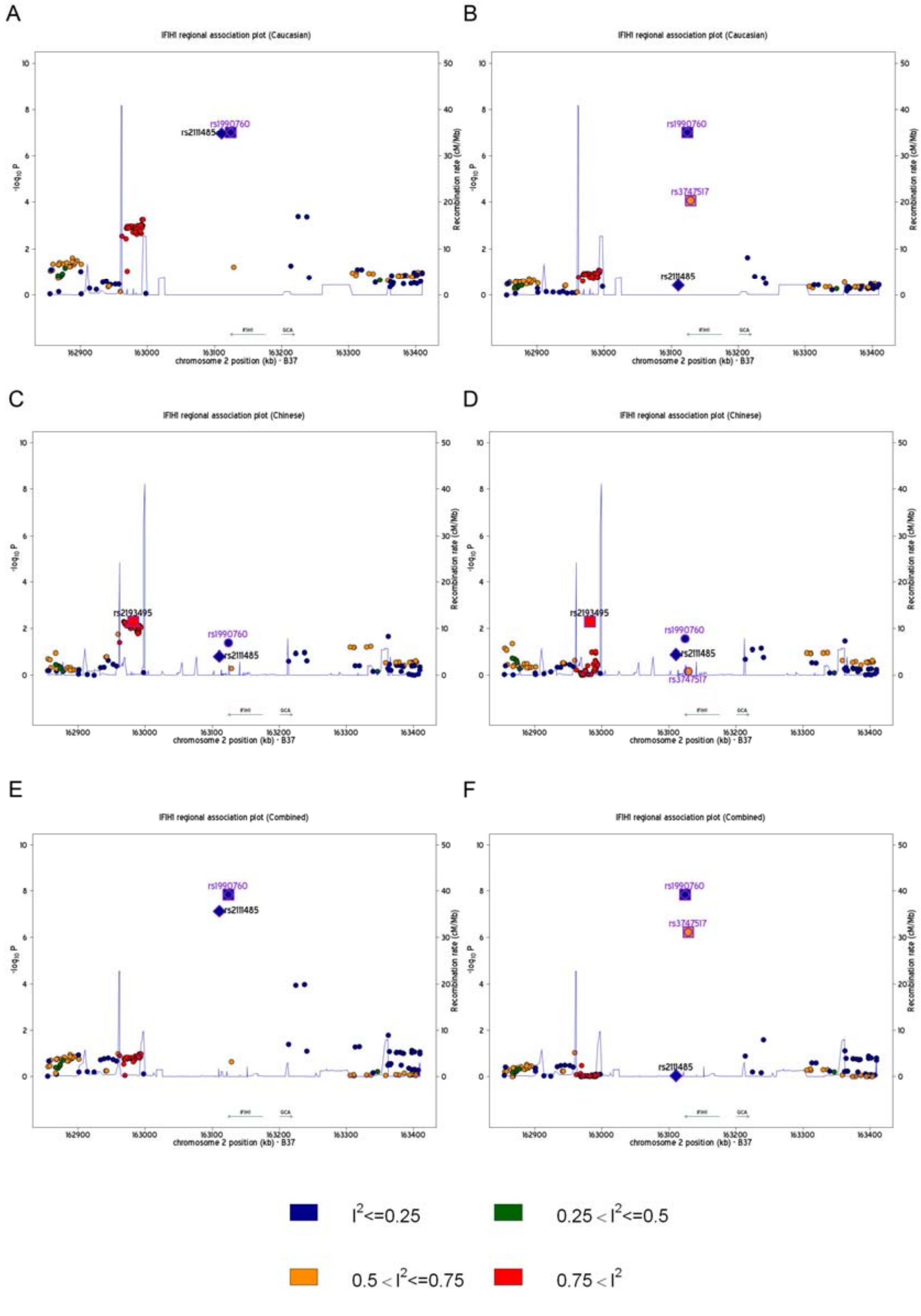
A
IFIH1 regional association plot (Caucasian)
B
IFIH1 regional association plot (Caucasian)
C
IFIH1 regional association plot (Chinese)
D
IFIH1 regional association plot (Chinese)
E
IFIH1 regional association plot (Combined)
F
IFIH1 regional association plot (Combined)
rs1990760
rs2111485
rs3747517
rs2193495
-log10 P
Recombination rate (cM/Mb)
chromosome 2 position (kb) - B37
IFIH1
GCA
r²<=0.25
0.25 < r²<=0.5
0.5 < r²<=0.75
0.75 < r²

f. IFIH1

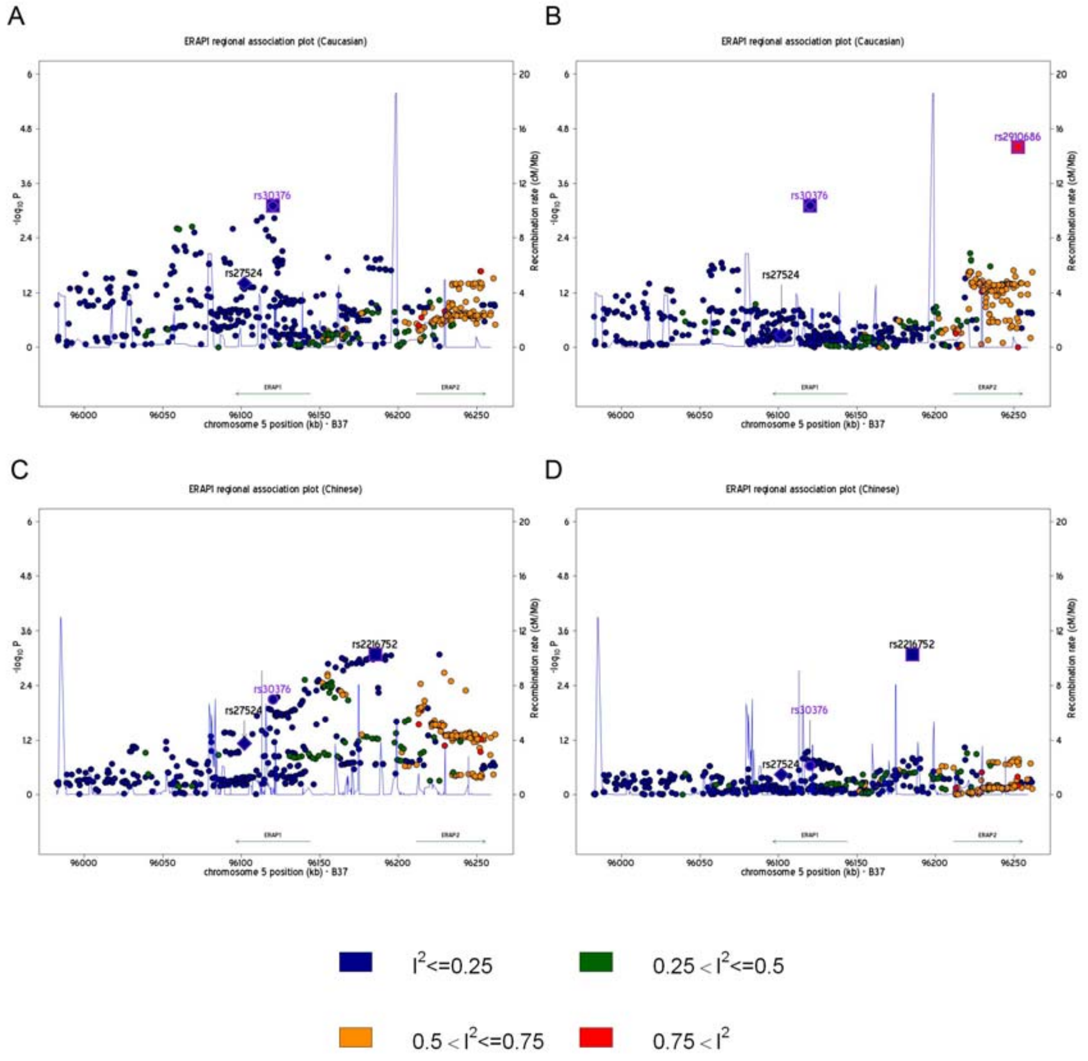

g. ERAP1/ERAP2 locus

Supplementary Figure 3: Regional association plot of four novel psoriasis risk loci and four independent secondary signals. The relative location of annotated genes and the direction of transcription are shown in the lower portion of the figure, and the chromosomal position is shown on the *x* axis. The blue line shows the recombination rate (estimated from HapMap data of CEU, CHB and Combined population) across the region (right *y* axis), and the left y axis shows the significance of the associations. The log10 *P* values from the log-additive genetic model for all SNPs in the region from the population meta analysis, trans-ethnic meta analysis or the conditional analysis. The square indicates the condition SNPs (these are the top or secondary SNPs); the circle labeled with rs IDs are reported psoriasis susceptibility SNPs, All circles and squares are color filled based on the heterogeneity results (such as I^2) in the trans-ethnic meta analysis. A, C and E are unconditional association results of Caucasian, Chinese and combined GWAS meta-analysis respectively. B, D, F are conditional association results of Caucasian, Chinese and combined GWAS meta-analysis respectively.

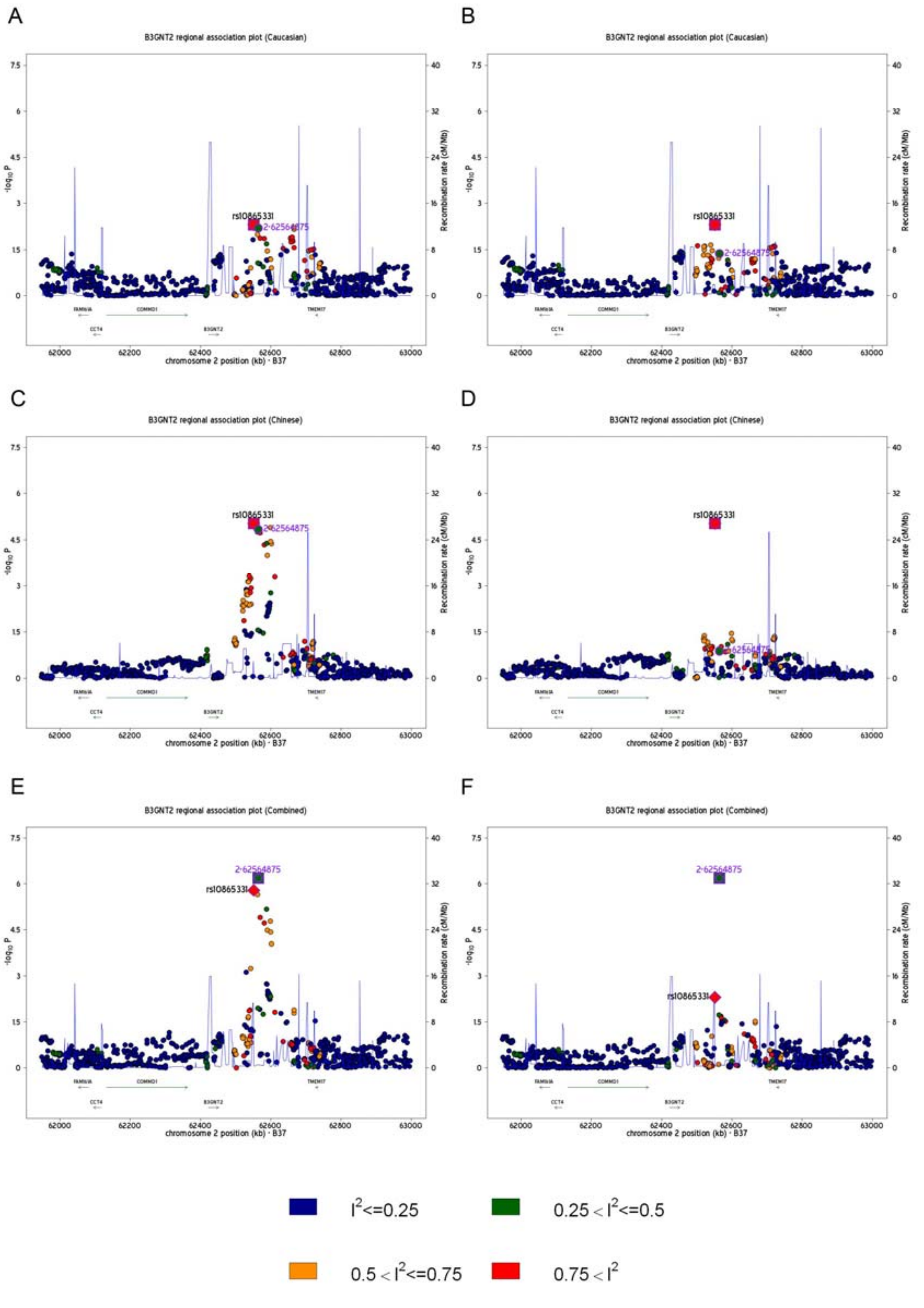
A
B3GNT2 regional association plot (Caucasian)
B
B3GNT2 regional association plot (Caucasian)
C
B3GNT2 regional association plot (Chinese)
D
B3GNT2 regional association plot (Chinese)
E
B3GNT2 regional association plot (Combined)
F
B3GNT2 regional association plot (Combined)
rs10865331
2-62564875
-log10 P
Recombination rate (cM/Mb)
chromosome 2 position (kb) - B37
FAM161A
CCT4
COMMD1
B3GNT2
TMEM17
r²<=0.25
0.25 < r²<=0.5
0.5 < r²<=0.75
0.75 < r²

a. B3GNT2

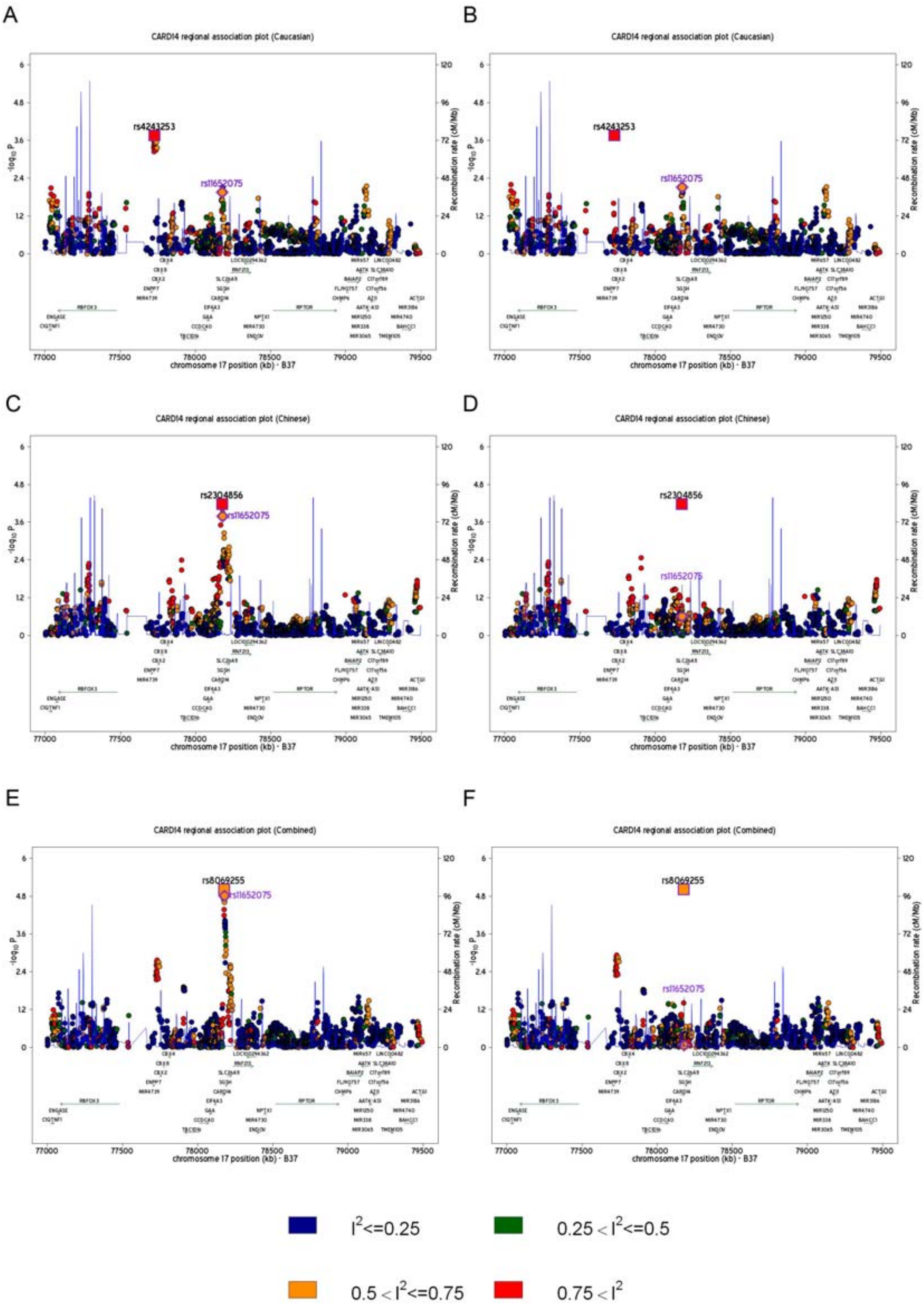
A
CARD14 regional association plot (Caucasian)
rs4243253
rs11652075
B
CARD14 regional association plot (Caucasian)
rs4243253
rs11652075
C
CARD14 regional association plot (Chinese)
rs2304856
rs11652075
D
CARD14 regional association plot (Chinese)
rs2304856
rs11652075
E
CARD14 regional association plot (Combined)
rs8069255
rs11652075
F
CARD14 regional association plot (Combined)
rs8069255
rs11652075
-log10 P
Recombination rate (cM/Mb)
chromosome 17 position (kb) - B37
I2<=0.25
0.25 < I2<=0.5
0.5 < I2<=0.75
0.75 < I2

b. CARD14

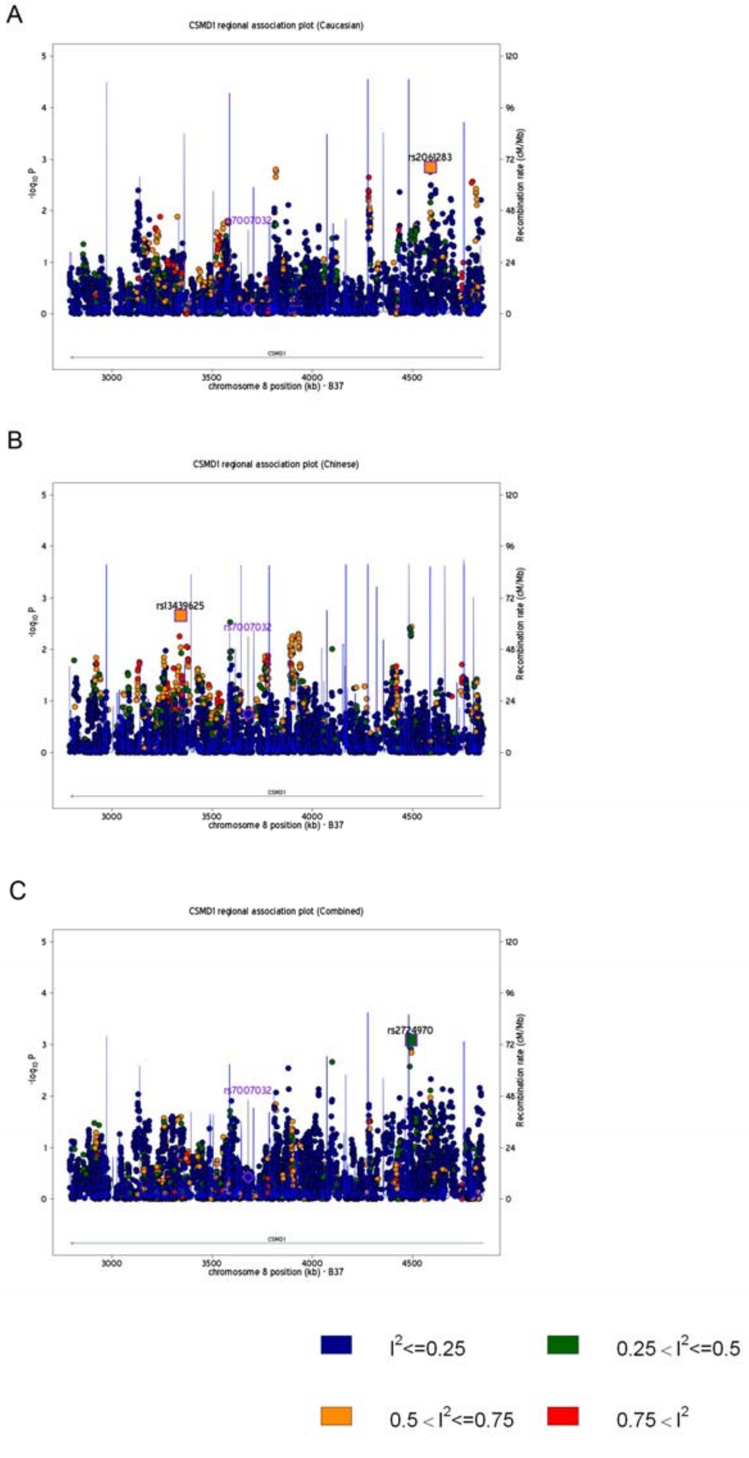
A
CSMD1 regional association plot (Caucasian)
rs2061283
rs7007032
chromosome 8 position (kb) - B37
B
CSMD1 regional association plot (Chinese)
rs13439625
rs7007032
chromosome 8 position (kb) - B37
C
CSMD1 regional association plot (Combined)
rs2724970
rs7007032
chromosome 8 position (kb) - B37
I2<=0.25
0.25 < I2<=0.5
0.5 < I2<=0.75
0.75 < I2

c. CSMD1

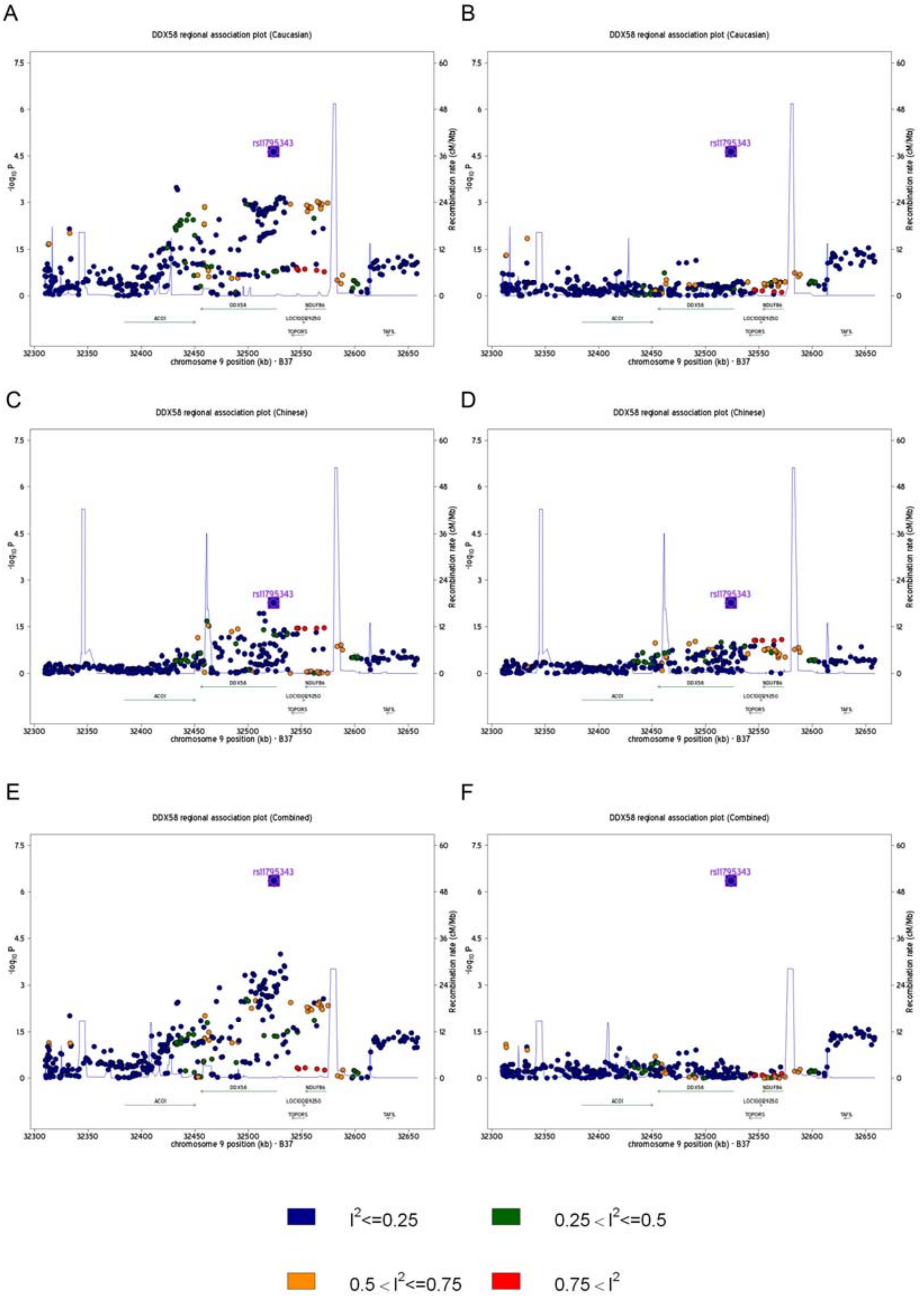
A
DDX58 regional association plot (Caucasian)
B
DDX58 regional association plot (Caucasian)
C
DDX58 regional association plot (Chinese)
D
DDX58 regional association plot (Chinese)
E
DDX58 regional association plot (Combined)
F
DDX58 regional association plot (Combined)
rs11795343
-log10 P
Recombination rate (cM/Mb)
chromosome 9 position (kb) - B37
ACO1
DDX58
NDUFB6
LOC100129250
TOPORS
TAF1L
I2<=0.25
0.25 < I2<=0.5
0.5 < I2<=0.75
0.75 < I2

d. DDX58

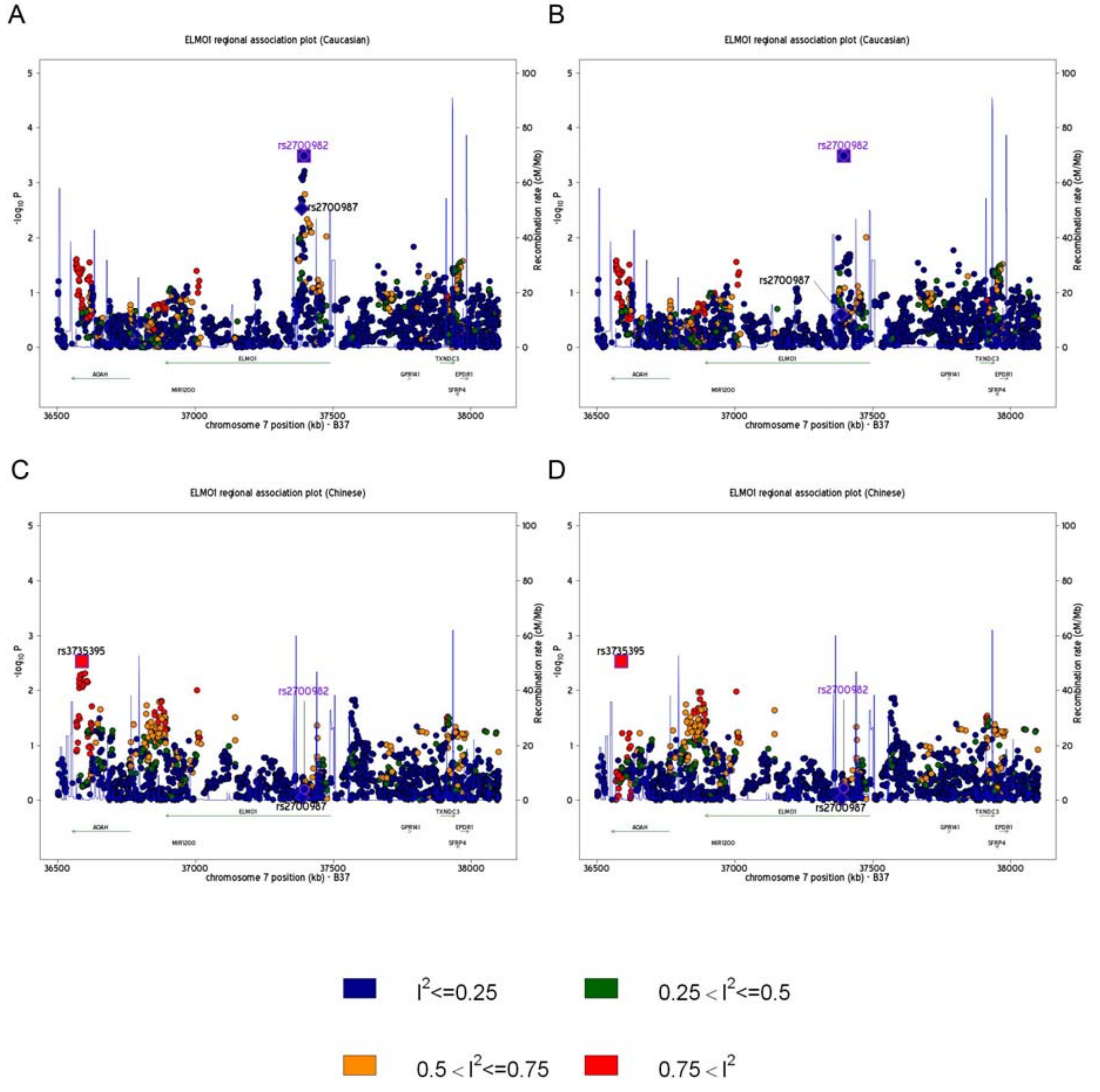
A
ELMO1 regional association plot (Caucasian)
B
ELMO1 regional association plot (Caucasian)
C
ELMO1 regional association plot (Chinese)
D
ELMO1 regional association plot (Chinese)
rs2700982
rs2700987
rs3735395
-log10 P
Recombination rate (cM/Mb)
chromosome 7 position (kb) - B37
I2<=0.25
0.25 < I2<=0.5
0.5 < I2<=0.75
0.75 < I2

e. ELMO1

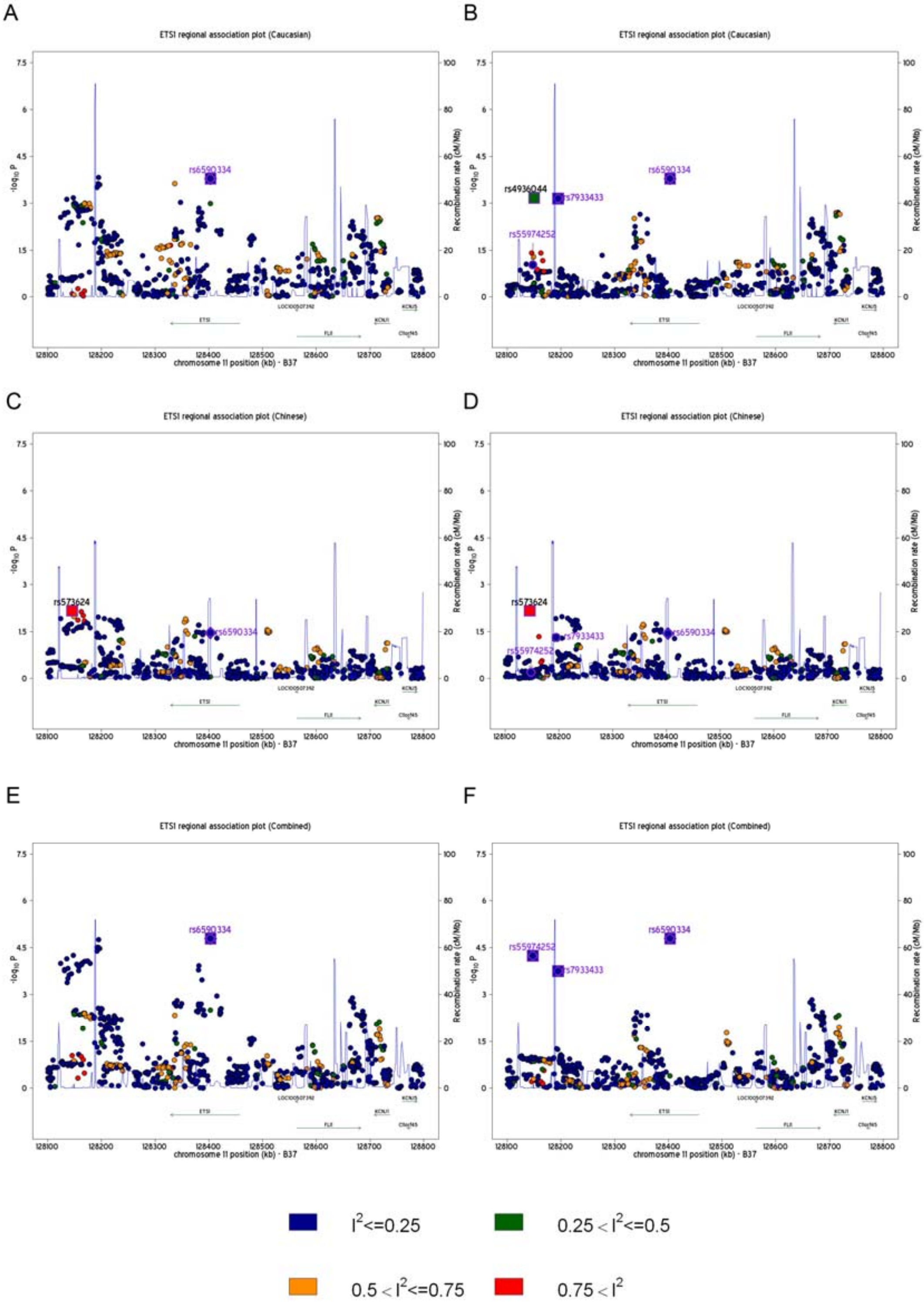
A
ETS1 regional association plot (Caucasian)
rs6590334
B
ETS1 regional association plot (Caucasian)
rs4936044
rs7933433
rs6590334
rs55974252
C
ETS1 regional association plot (Chinese)
rs573624
rs6590334
D
ETS1 regional association plot (Chinese)
rs573624
rs7933433
rs6590334
rs55974252
E
ETS1 regional association plot (Combined)
rs6590334
F
ETS1 regional association plot (Combined)
rs55974252
rs6590334
rs7933433
-log10 P
Recombination rate (cM/Mb)
chromosome 11 position (kb) - B37
ETS1
FLI1
KCNJ1
KCNJ5
LOC100507392
C11orf45
I^2<=0.25
0.25 < I^2<=0.5
0.5 < I^2<=0.75
0.75 < I^2

f. ETS1

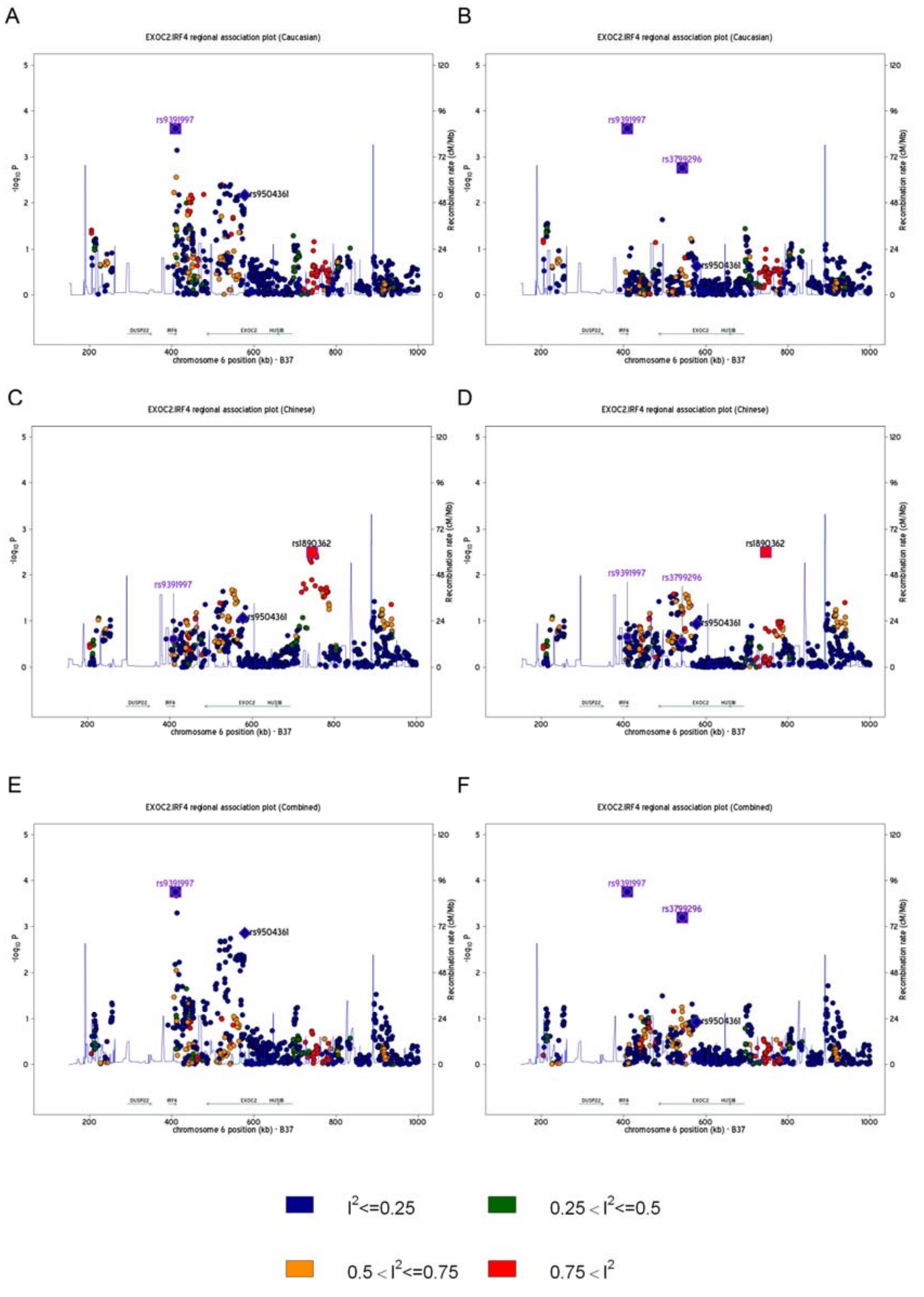
A
EXOC2.IRF4 regional association plot (Caucasian)
rs9391997
rs9504361
B
EXOC2.IRF4 regional association plot (Caucasian)
rs9391997
rs3799296
rs9504361
C
EXOC2.IRF4 regional association plot (Chinese)
rs1890362
rs9391997
rs9504361
D
EXOC2.IRF4 regional association plot (Chinese)
rs1890362
rs9391997
rs3799296
rs9504361
E
EXOC2.IRF4 regional association plot (Combined)
rs9391997
rs9504361
F
EXOC2.IRF4 regional association plot (Combined)
rs9391997
rs3799296
rs9504361
-log10 P
Recombination rate (cM/Mb)
chromosome 6 position (kb) - B37
DUSP22
IRF4
EXOC2
HUS1B
I2<=0.25
0.25 < I2<=0.5
0.5 < I2<=0.75
0.75 < I2

g. EXOC2.IRF4

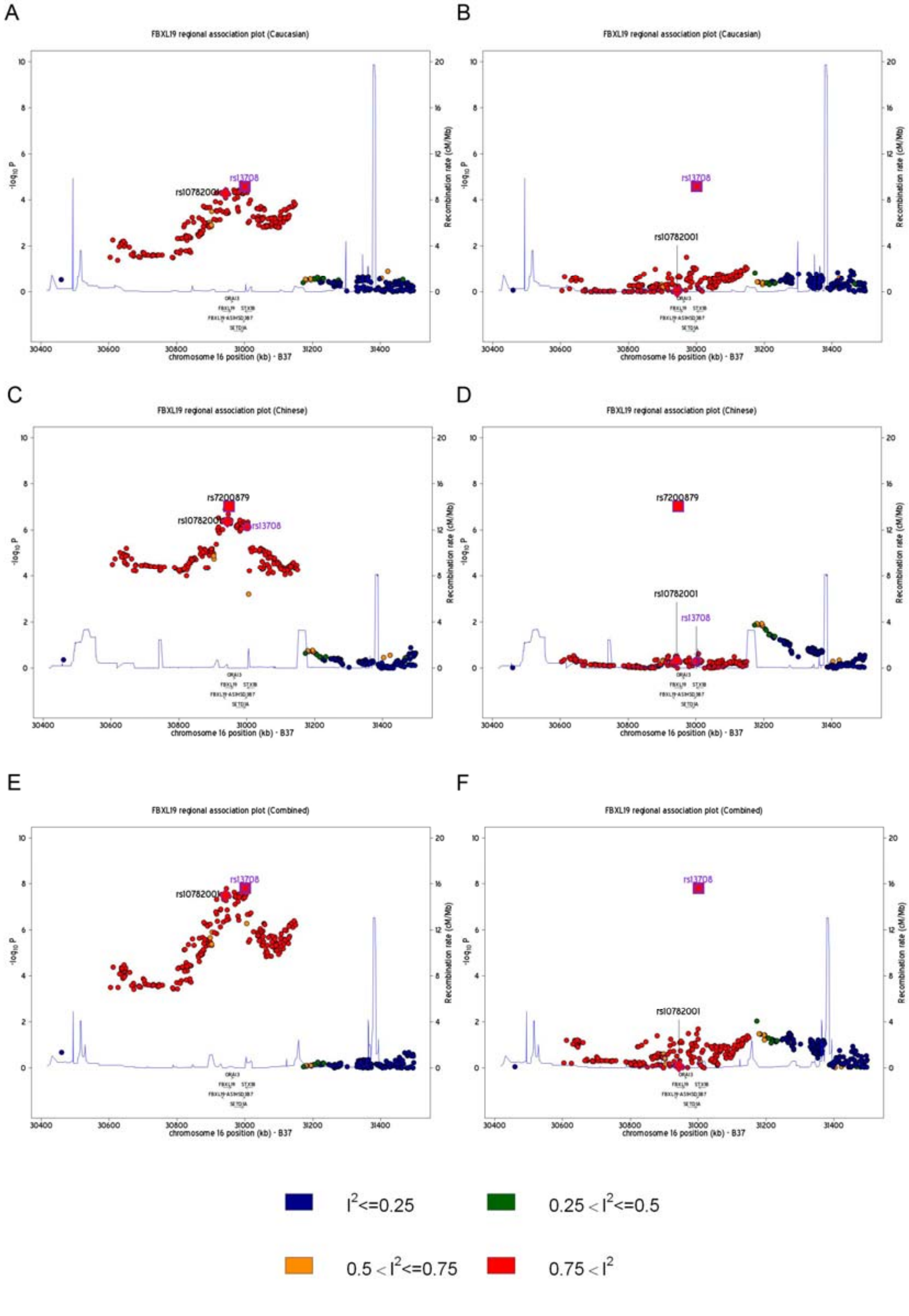

A
FBXL19 regional association plot (Caucasian)
B
FBXL19 regional association plot (Caucasian)
C
FBXL19 regional association plot (Chinese)
D
FBXL19 regional association plot (Chinese)
E
FBXL19 regional association plot (Combined)
F
FBXL19 regional association plot (Combined)
rs13708
rs10782001
rs7200879
-log10 P
Recombination rate (cM/Mb)
chromosome 16 position (kb) - B37
I2<=0.25
0.25 < I2<=0.5
0.5 < I2<=0.75
0.75 < I2

h. FBXL19

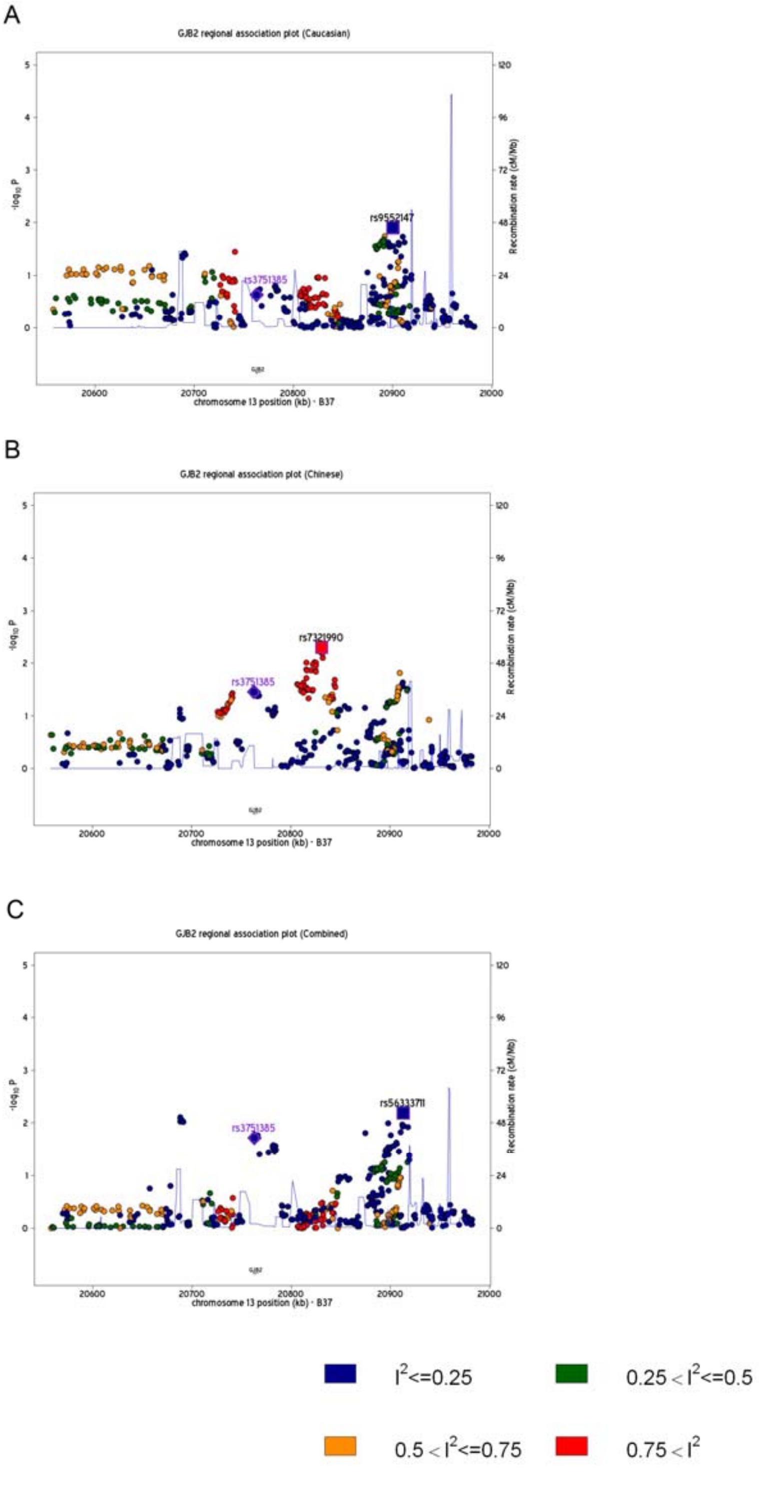
A
GJB2 regional association plot (Caucasian)
rs9552147
rs3751385
B
GJB2 regional association plot (Chinese)
rs7321990
rs3751385
C
GJB2 regional association plot (Combined)
rs56333711
rs3751385
-log10 P
Recombination rate (cM/Mb)
chromosome 13 position (kb) - B37
I2<=0.25
0.25<I2<=0.5
0.5<I2<=0.75
0.75<I2

i. GJB2

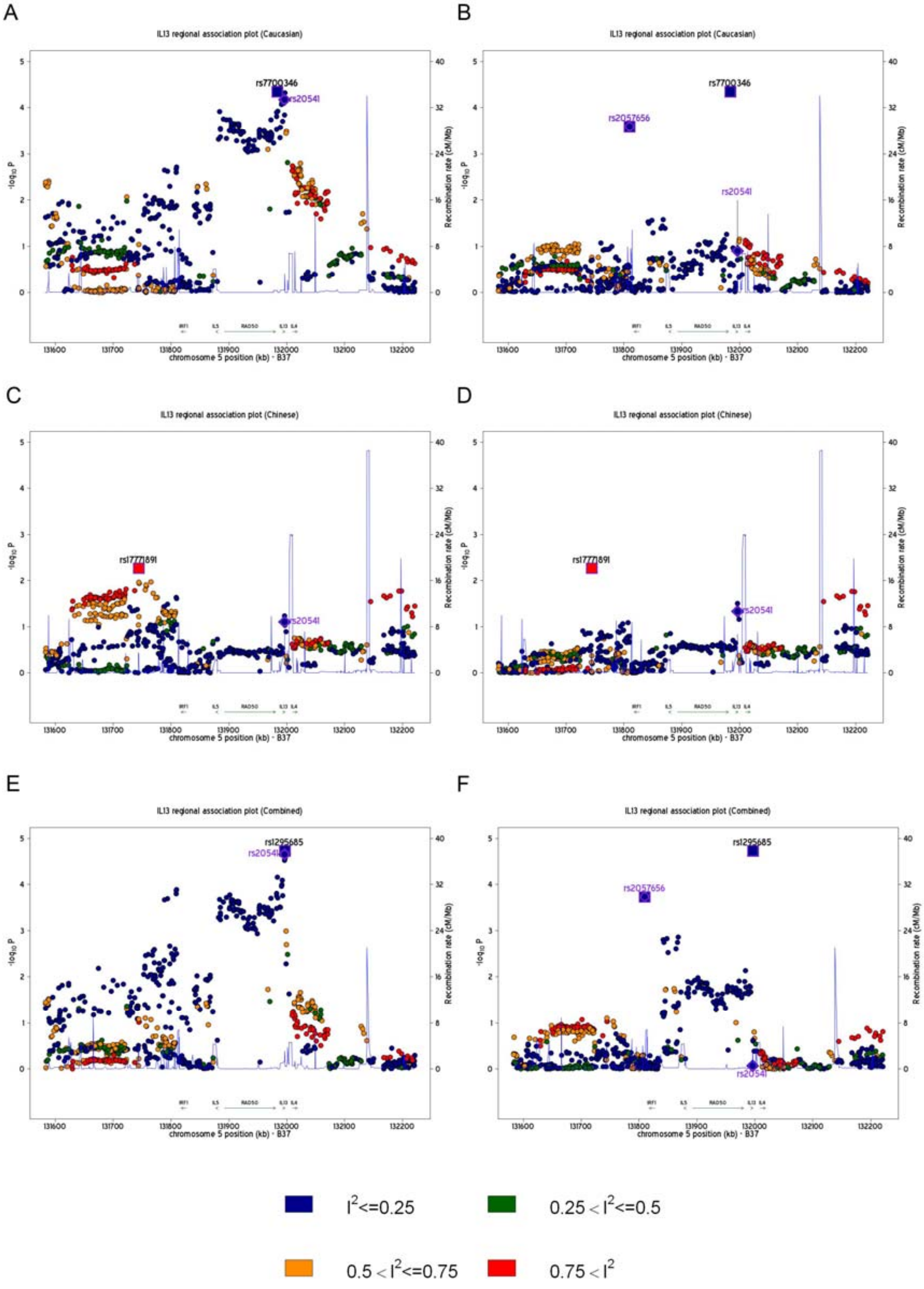
A
IL13 regional association plot (Caucasian)
rs7700346
rs20541
B
IL13 regional association plot (Caucasian)
rs2057656
rs7700346
rs20541
C
IL13 regional association plot (Chinese)
rs17771891
rs20541
D
IL13 regional association plot (Chinese)
rs17771891
rs20541
E
IL13 regional association plot (Combined)
rs1295685
rs20541
F
IL13 regional association plot (Combined)
rs1295685
rs2057656
rs20541
-log10 P
Recombination rate (cM/Mb)
chromosome 5 position (kb) - B37
IRF1
IL5
RAD50
IL13
IL4
I2<=0.25
0.25 < I2<=0.5
0.5 < I2<=0.75
0.75 < I2

j. IL13

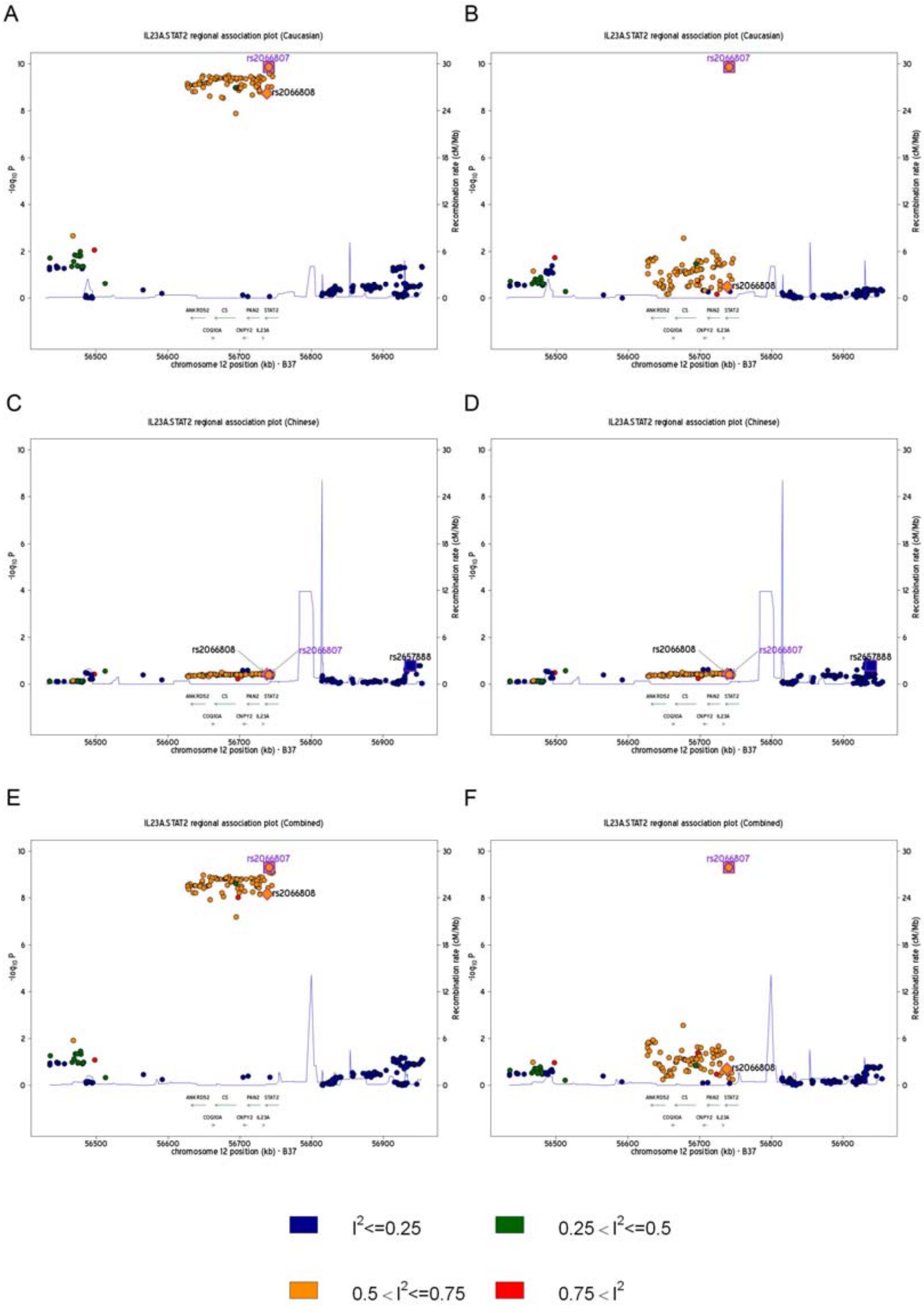

k. IL23A.STAT2

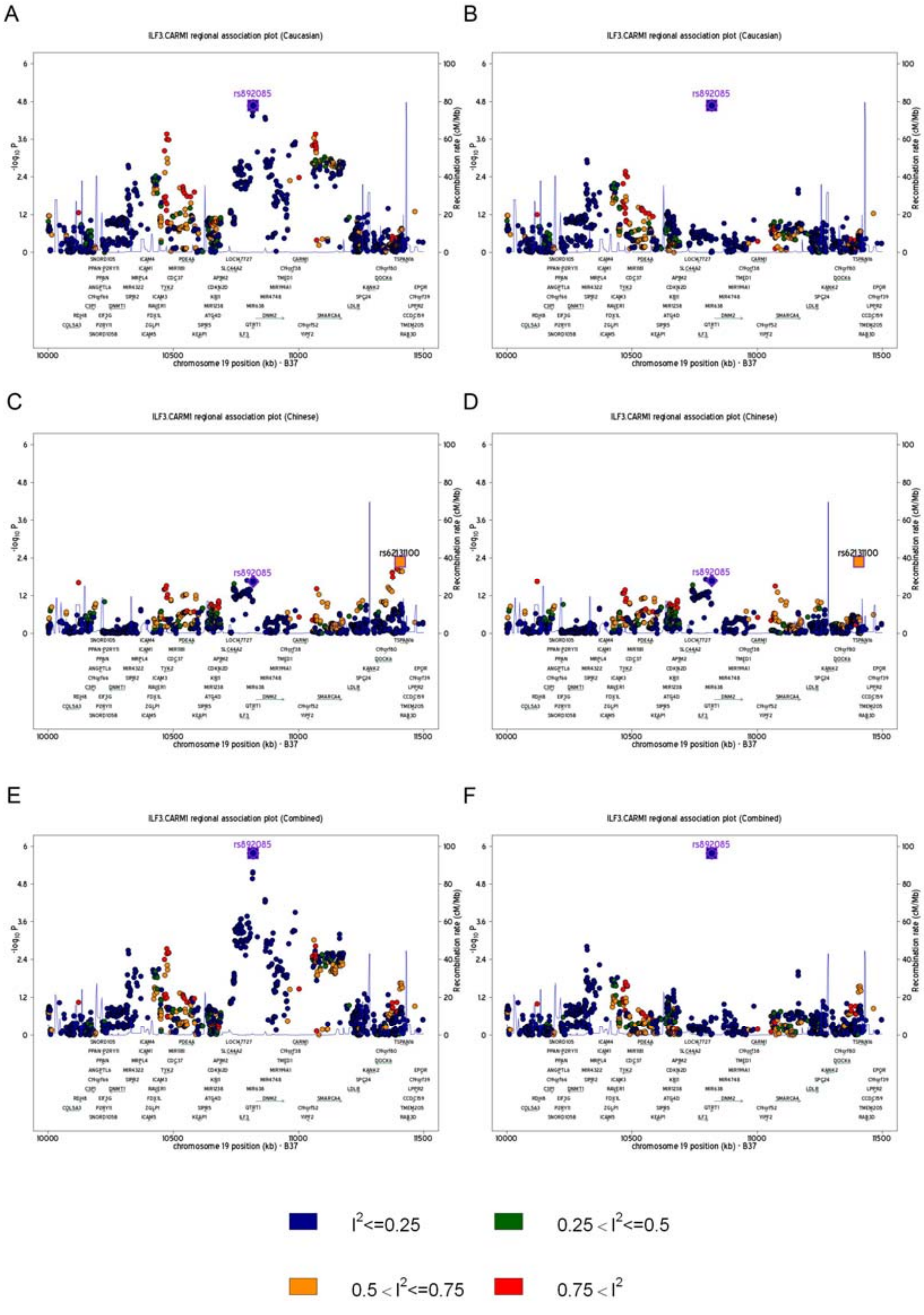
A
ILF3.CARM1 regional association plot (Caucasian)
rs892085
B
ILF3.CARM1 regional association plot (Caucasian)
rs892085
C
ILF3.CARM1 regional association plot (Chinese)
rs892085
rs62131100
D
ILF3.CARM1 regional association plot (Chinese)
rs892085
rs62131100
E
ILF3.CARM1 regional association plot (Combined)
rs892085
F
ILF3.CARM1 regional association plot (Combined)
rs892085
-log10 P
Recombination rate (cM/Mb)
chromosome 19 position (kb) - B37
I2<=0.25
0.25 < I2<=0.5
0.5 < I2<=0.75
0.75 < I2

l. ILF3.CARM1

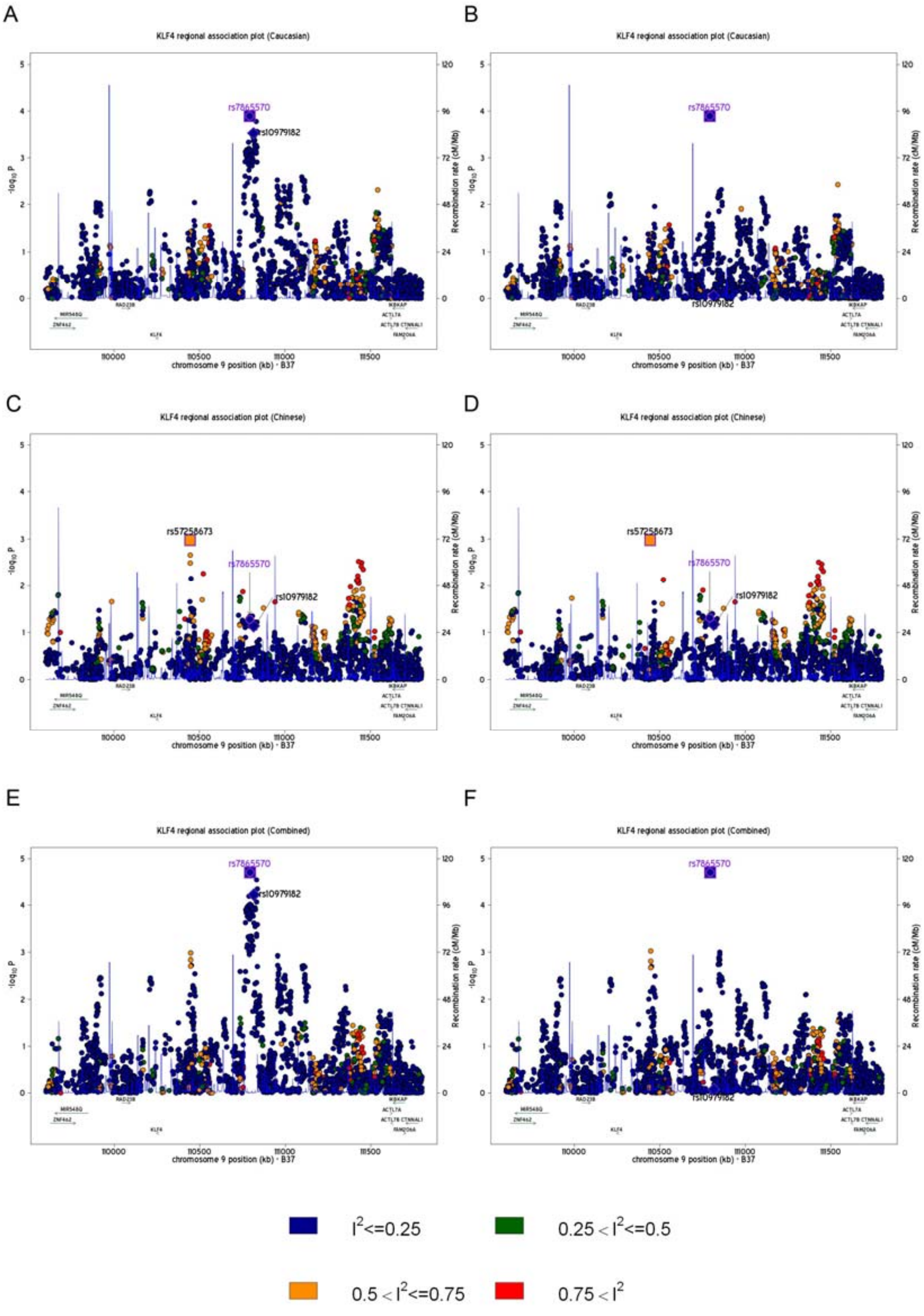

A
KLF4 regional association plot (Caucasian)
rs7865570
rs10979182
B
KLF4 regional association plot (Caucasian)
rs7865570
rs10979182
C
KLF4 regional association plot (Chinese)
rs57258673
rs7865570
rs10979182
D
KLF4 regional association plot (Chinese)
rs57258673
rs7865570
rs10979182
E
KLF4 regional association plot (Combined)
rs7865570
rs10979182
F
KLF4 regional association plot (Combined)
rs7865570
rs10979182
-log10 P
Recombination rate (cM/Mb)
chromosome 9 position (kb) - B37
110000
110500
111000
111500
I^2<=0.25
0.25 < I^2<=0.5
0.5 < I^2<=0.75
0.75 < I^2

m. KLF4

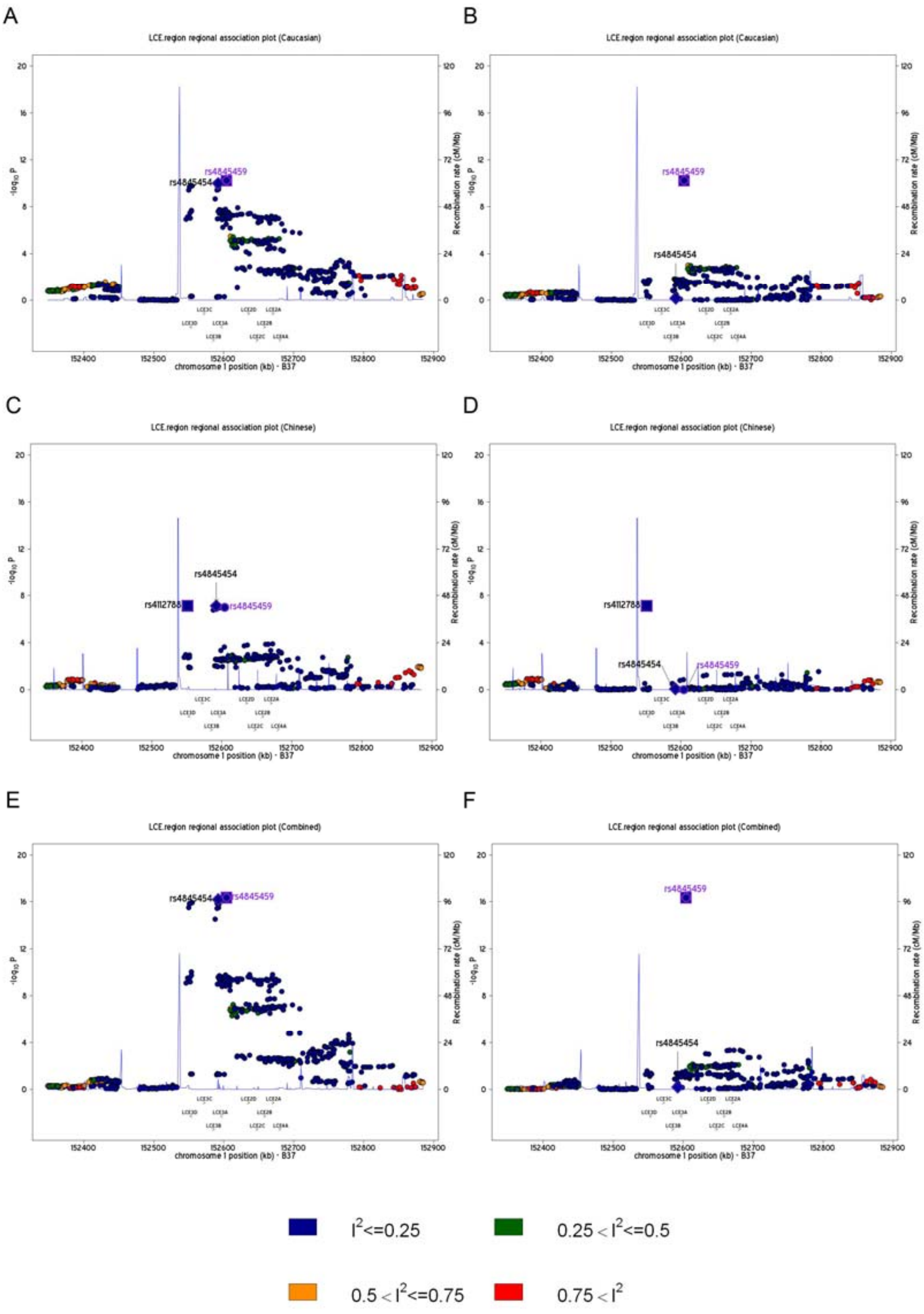
A
LCE.region regional association plot (Caucasian)
rs4845459
rs4845454
B
LCE.region regional association plot (Caucasian)
rs4845459
rs4845454
C
LCE.region regional association plot (Chinese)
rs4845454
rs4112788
rs4845459
D
LCE.region regional association plot (Chinese)
rs4112788
rs4845454
rs4845459
E
LCE.region regional association plot (Combined)
rs4845454
rs4845459
F
LCE.region regional association plot (Combined)
rs4845459
rs4845454
-log10 P
Recombination rate (cM/Mb)
chromosome 1 position (kb) - B37
I2<=0.25
0.25 < I2<=0.5
0.5 < I2<=0.75
0.75 < I2

n. LCE region

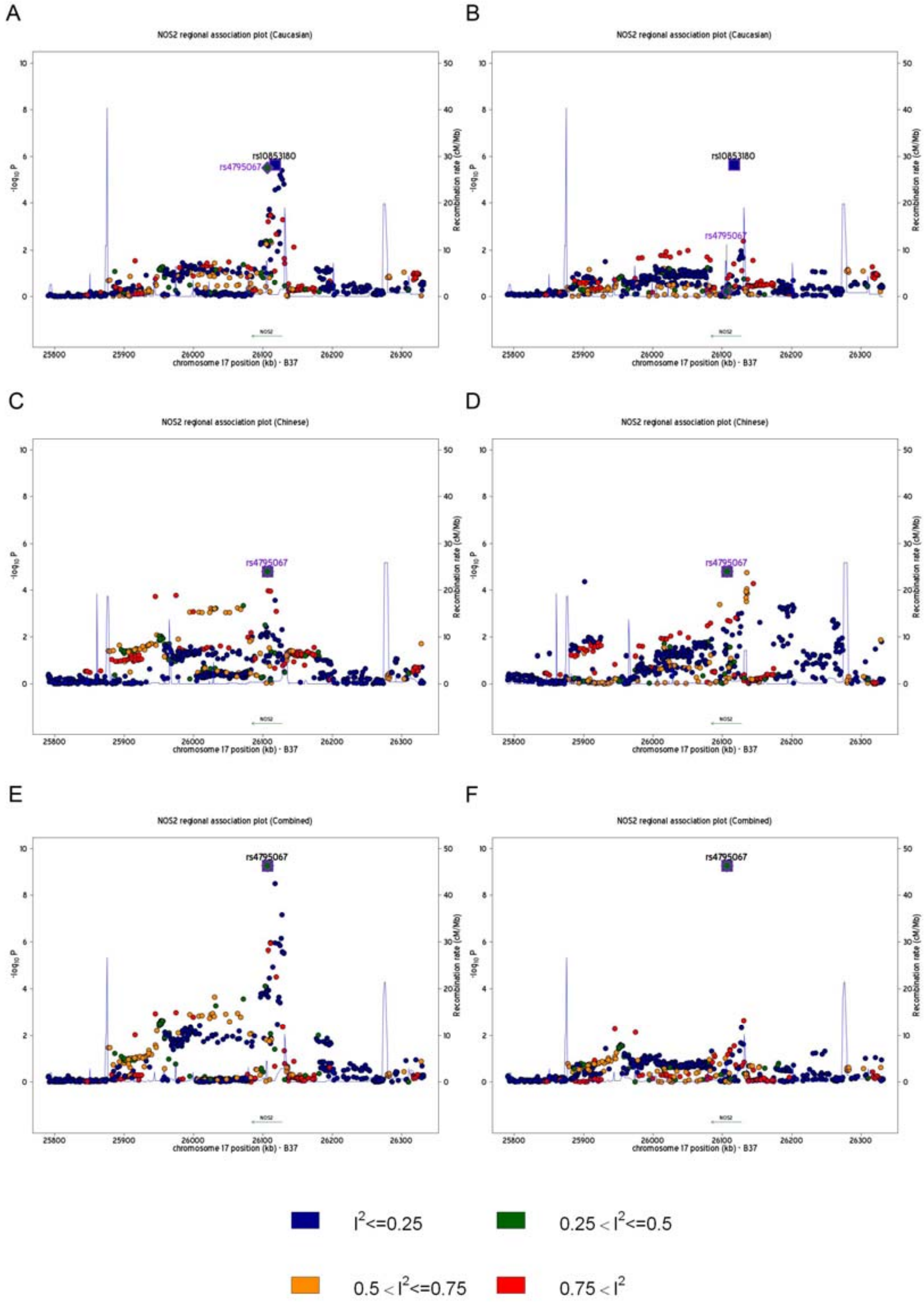
A
NOS2 regional association plot (Caucasian)
rs10853180
rs4795067
B
NOS2 regional association plot (Caucasian)
rs10853180
rs4795067
C
NOS2 regional association plot (Chinese)
rs4795067
D
NOS2 regional association plot (Chinese)
rs4795067
E
NOS2 regional association plot (Combined)
rs4795067
F
NOS2 regional association plot (Combined)
rs4795067
-log10 P
Recombination rate (cM/Mb)
chromosome 17 position (kb) - B37
NOS2
I2<=0.25
0.25 < I2<=0.5
0.5 < I2<=0.75
0.75 < I2

o. NOS2

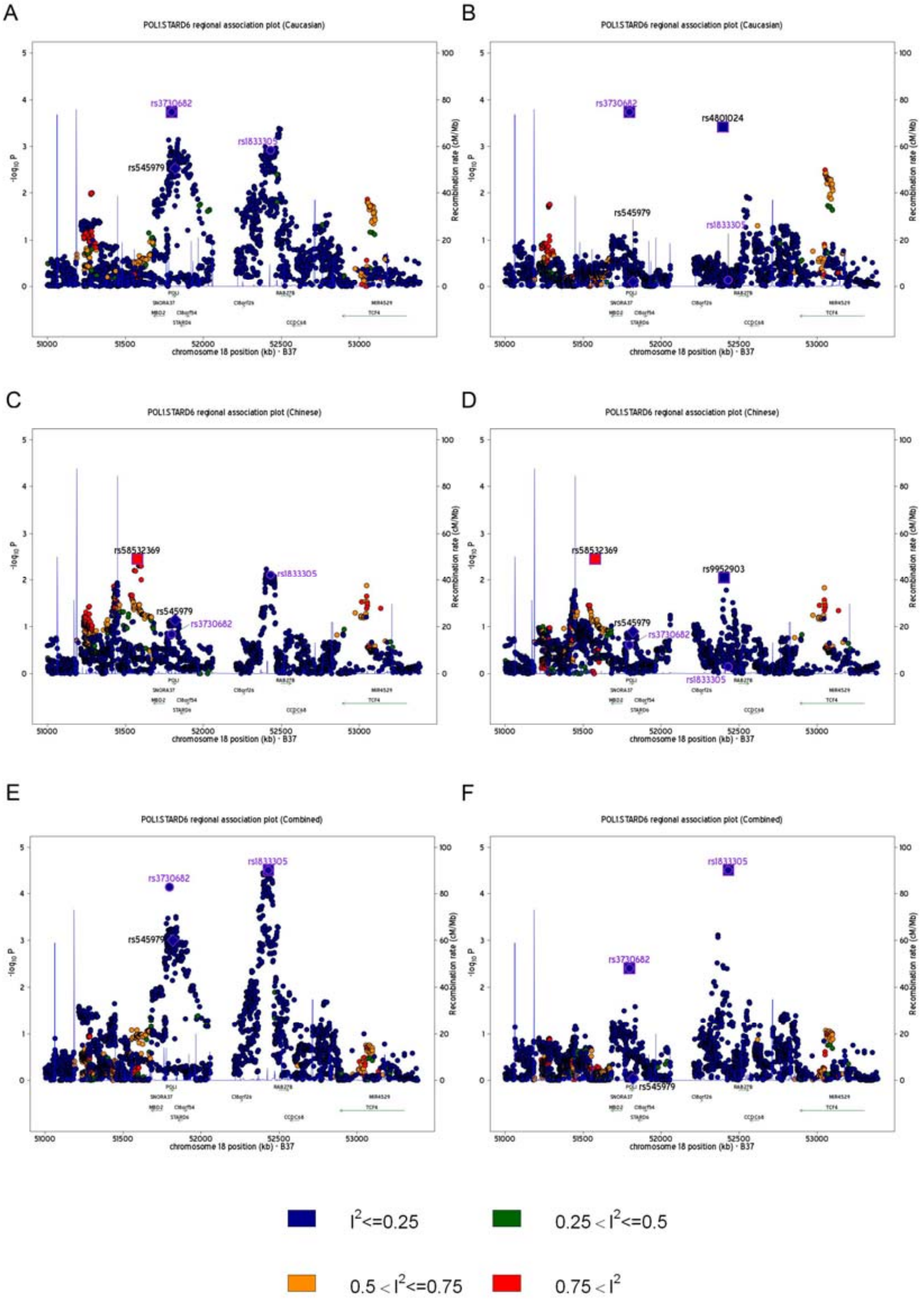

p. POL1.STARD6

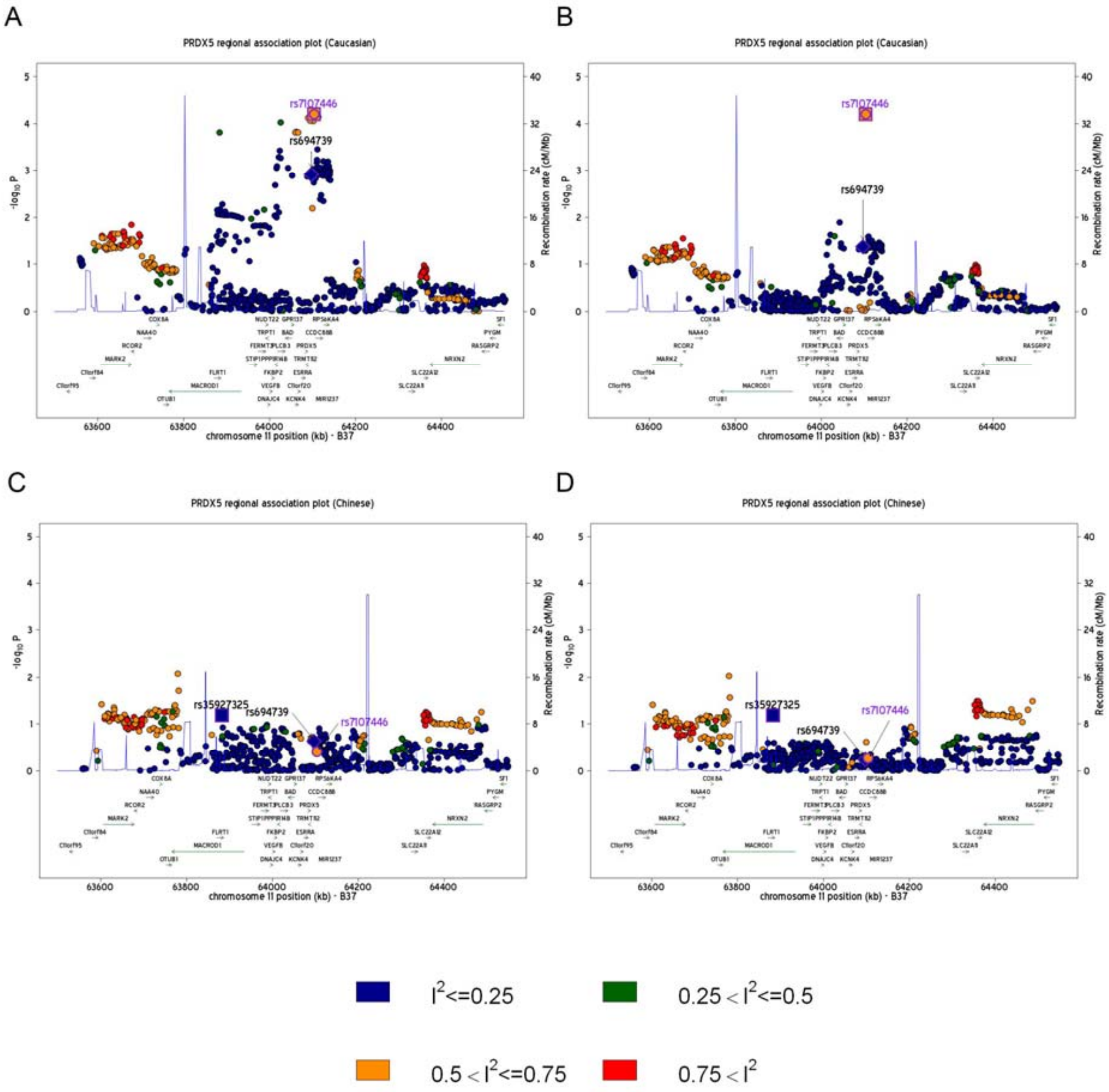
A
PRDX5 regional association plot (Caucasian)
rs7107446
rs694739
B
PRDX5 regional association plot (Caucasian)
rs7107446
rs694739
C
PRDX5 regional association plot (Chinese)
rs35927325
rs694739
rs7107446
D
PRDX5 regional association plot (Chinese)
rs35927325
rs7107446
rs694739
-log10 P
Recombination rate (cM/Mb)
chromosome 11 position (kb) - B37
I^2<=0.25
0.25 < I^2<=0.5
0.5 < I^2<=0.75
0.75 < I^2

q. PRDX5

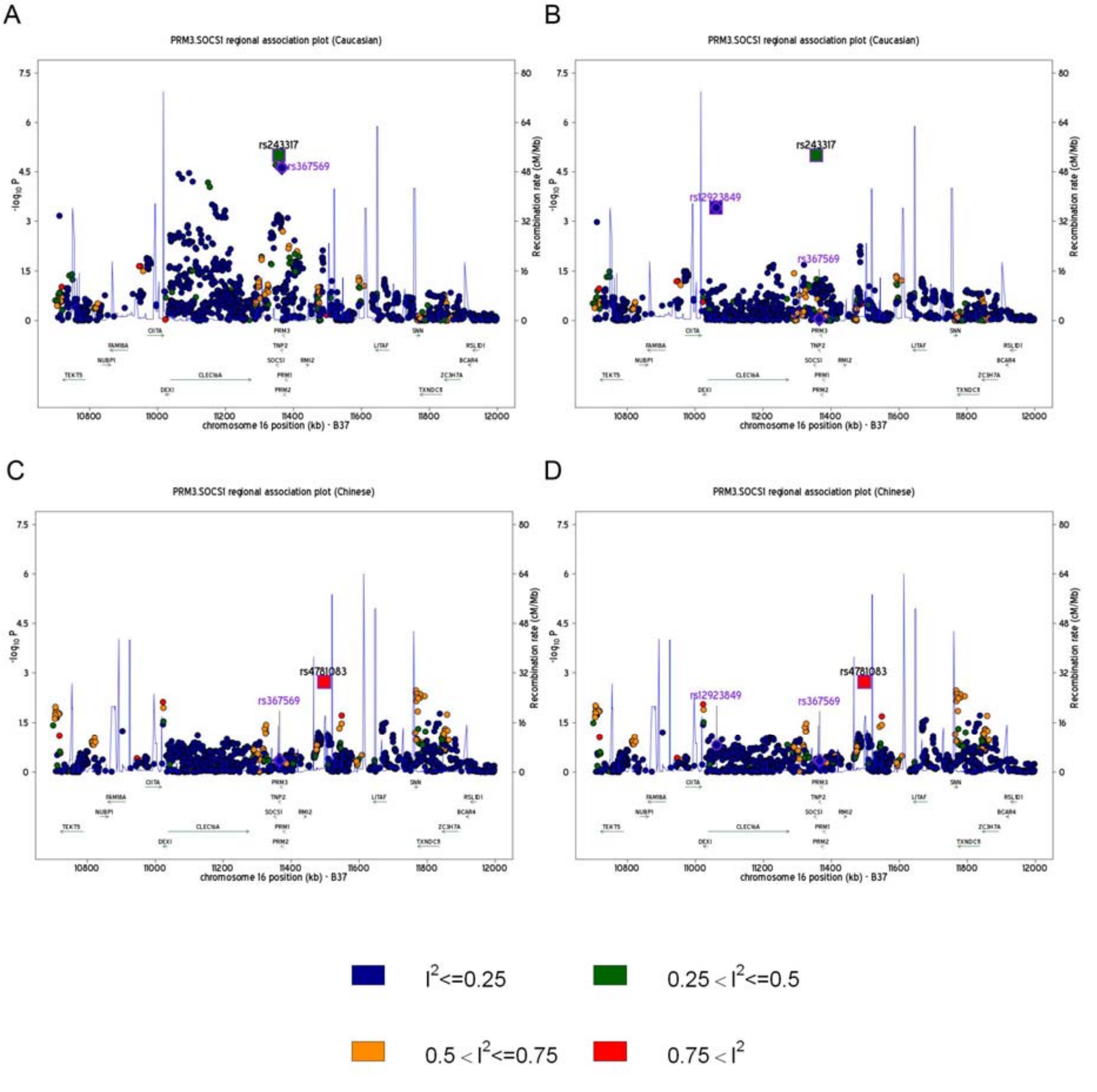
A
PRM3.SOCS1 regional association plot (Caucasian)
rs243317
rs367569
B
PRM3.SOCS1 regional association plot (Caucasian)
rs243317
rs12923849
rs367569
C
PRM3.SOCS1 regional association plot (Chinese)
rs4781083
rs367569
D
PRM3.SOCS1 regional association plot (Chinese)
rs4781083
rs12923849
rs367569
-log10 P
Recombination rate (cM/Mb)
chromosome 16 position (kb) - B37
I²<=0.25
0.25 < I²<=0.5
0.5 < I²<=0.75
0.75 < I²

r. PRM3.SOCS1

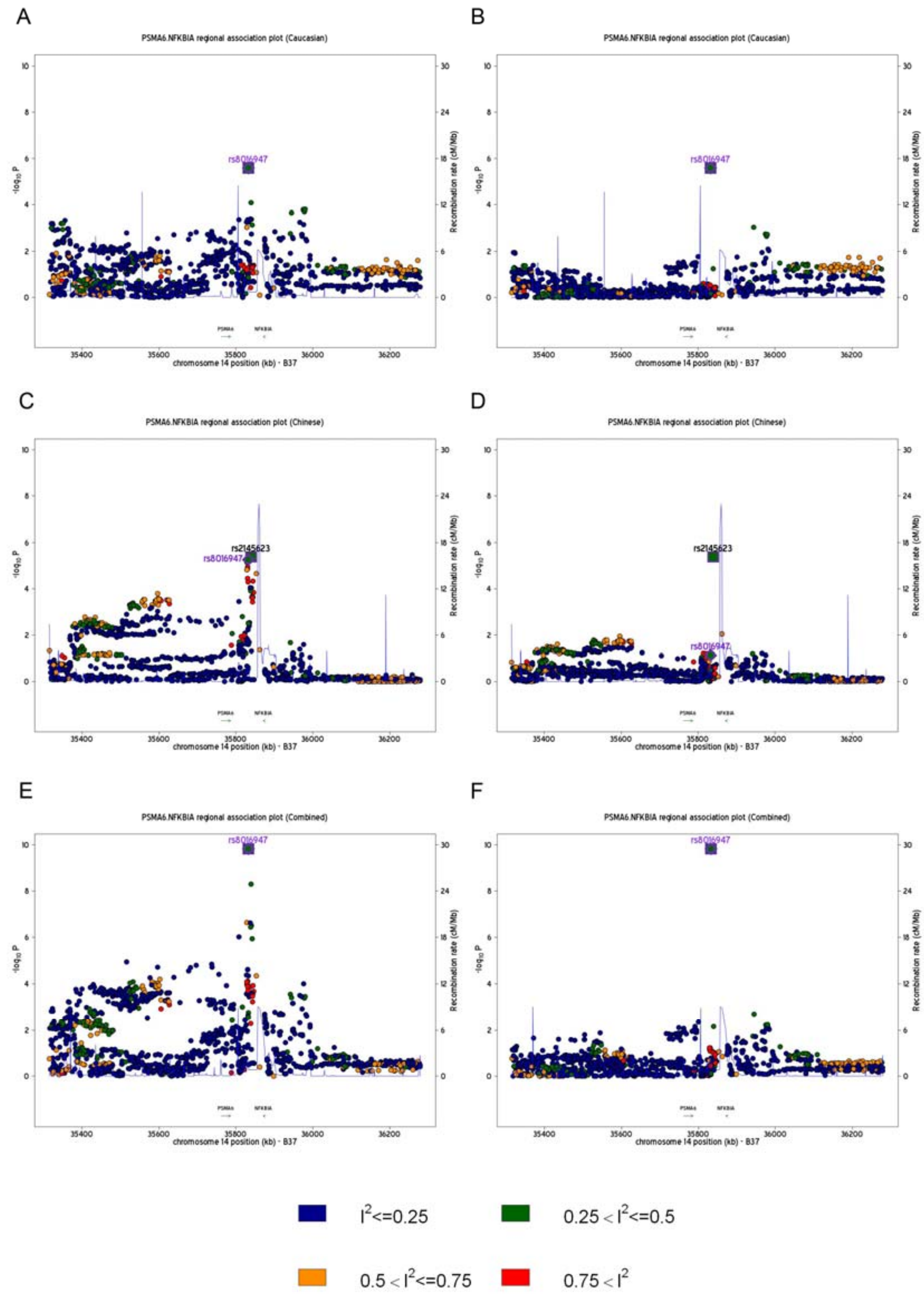

s. PSMA6.NFKBIA

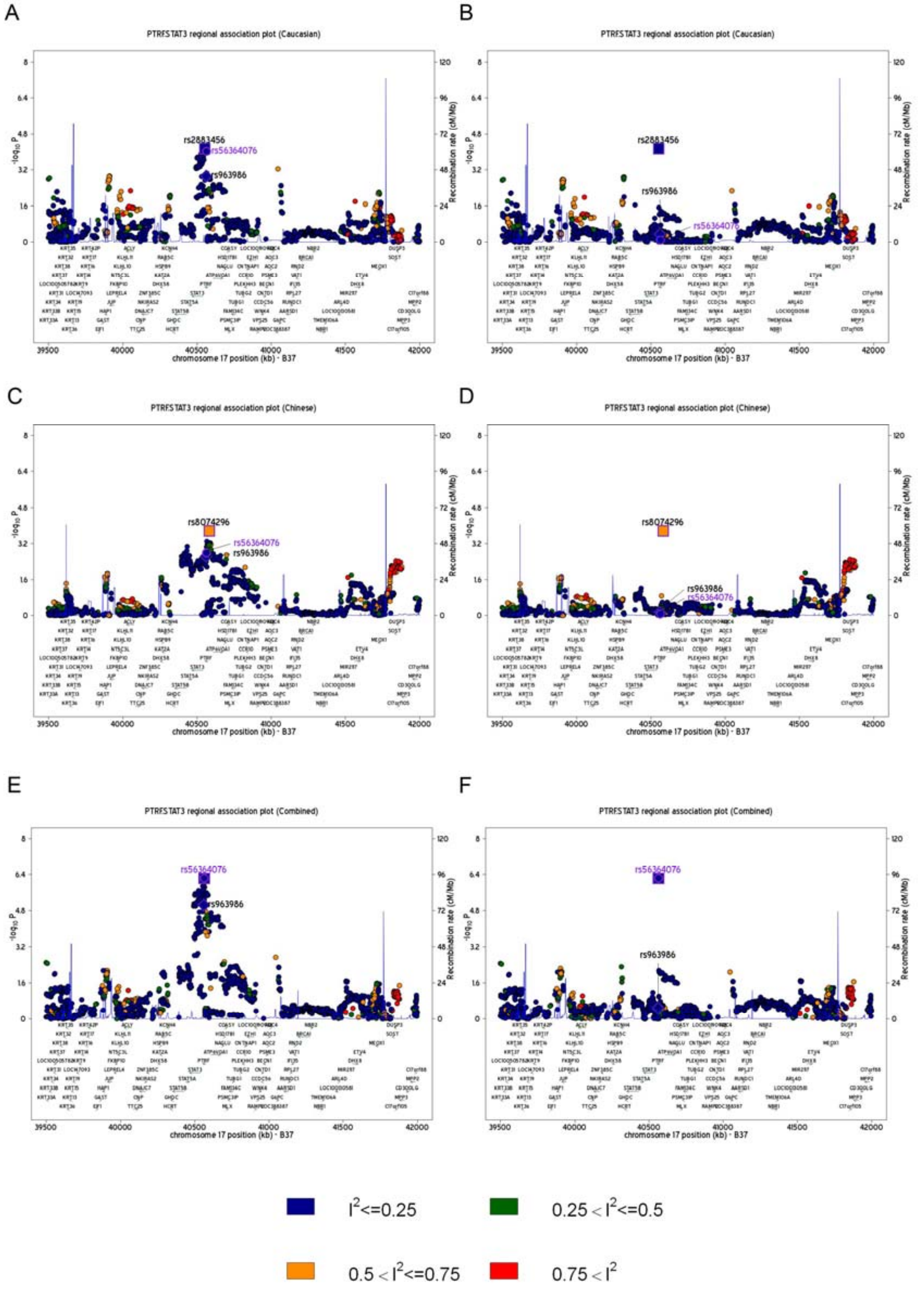

A
PTRFSTAT3 regional association plot (Caucasian)
rs2883456
rs56364076
rs963986
B
PTRFSTAT3 regional association plot (Caucasian)
rs2883456
rs963986
rs56364076
C
PTRFSTAT3 regional association plot (Chinese)
rs8074296
rs56364076
rs963986
D
PTRFSTAT3 regional association plot (Chinese)
rs8074296
rs963986
rs56364076
E
PTRFSTAT3 regional association plot (Combined)
rs56364076
rs963986
F
PTRFSTAT3 regional association plot (Combined)
rs56364076
rs963986
chromosome 17 position (kb) - B37
Recombination rate (cM/Mb)
-log10 P
I2<=0.25
0.25 < I2<=0.5
0.5 < I2<=0.75
0.75 < I2

t. PTRF.STAT3

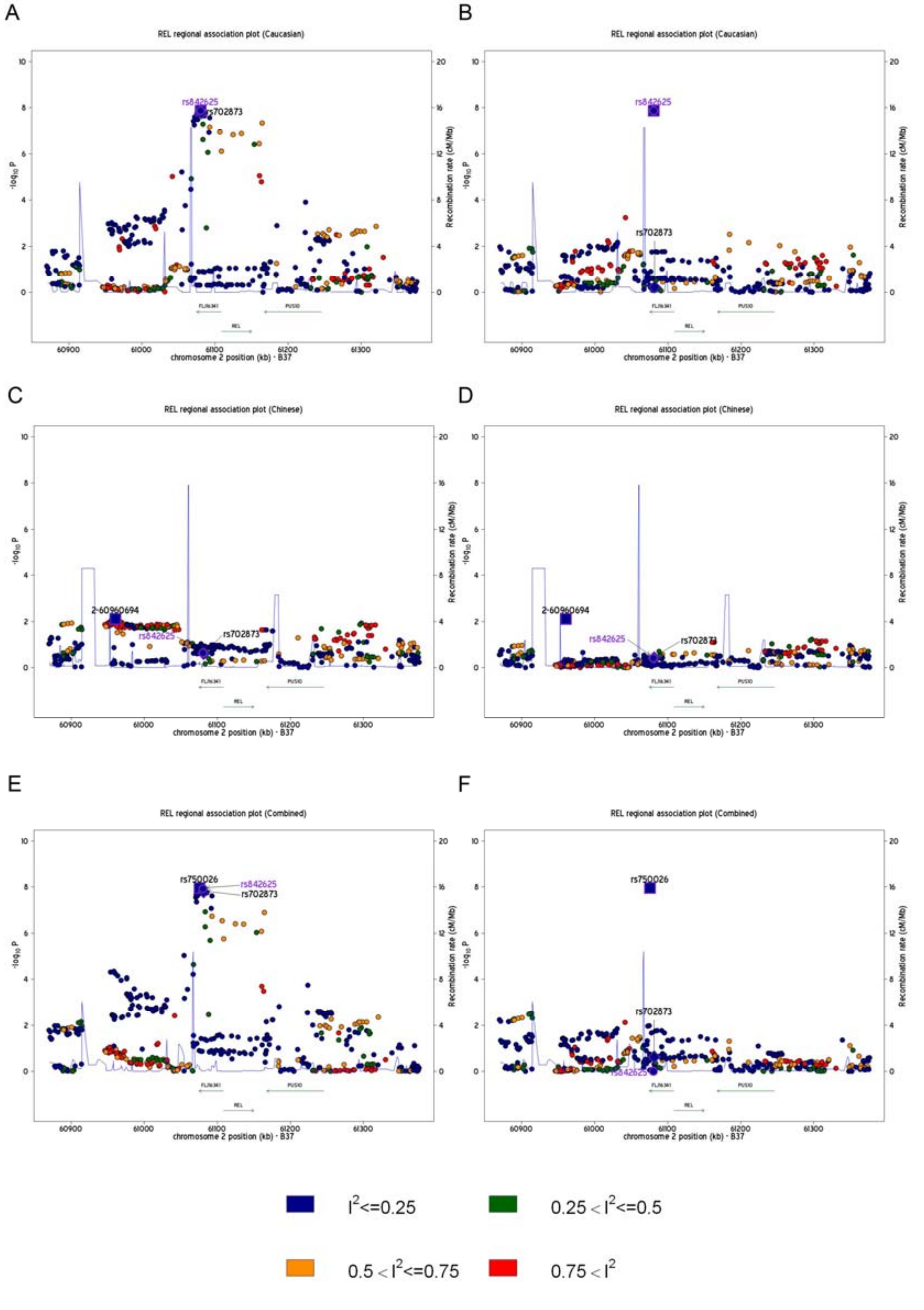
A
REL regional association plot (Caucasian)
rs842625
rs702873
B
REL regional association plot (Caucasian)
rs842625
rs702873
C
REL regional association plot (Chinese)
2-60960694
rs842625
rs702873
D
REL regional association plot (Chinese)
2-60960694
rs842625
rs702873
E
REL regional association plot (Combined)
rs750026
rs842625
rs702873
F
REL regional association plot (Combined)
rs750026
rs702873
rs842625
-log10 P
Recombination rate (cM/Mb)
chromosome 2 position (kb) - B37
FLJ16341
PUS10
REL
I^2<=0.25
0.25 < I^2<=0.5
0.5 < I^2<=0.75
0.75 < I^2

u. REL

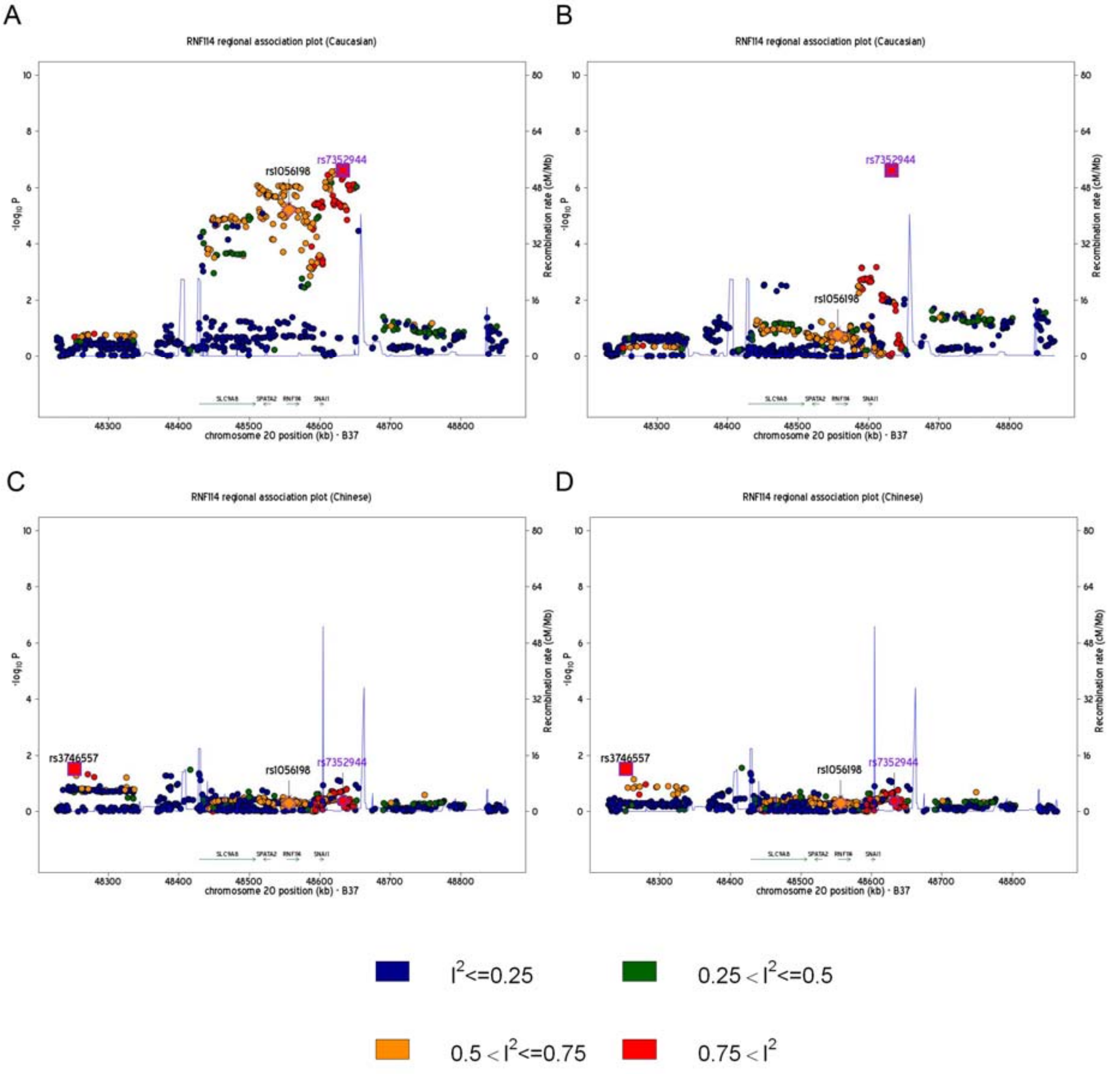
A
RNF114 regional association plot (Caucasian)
B
RNF114 regional association plot (Caucasian)
C
RNF114 regional association plot (Chinese)
D
RNF114 regional association plot (Chinese)
-log10 P
Recombination rate (cM/Mb)
chromosome 20 position (kb) - B37
rs7352944
rs1056198
rs3746557
SLC9A8
SPATA2
RNF114
SNAI1
I2<=0.25
0.25 < I2<=0.5
0.5 < I2<=0.75
0.75 < I2

v. RNF114

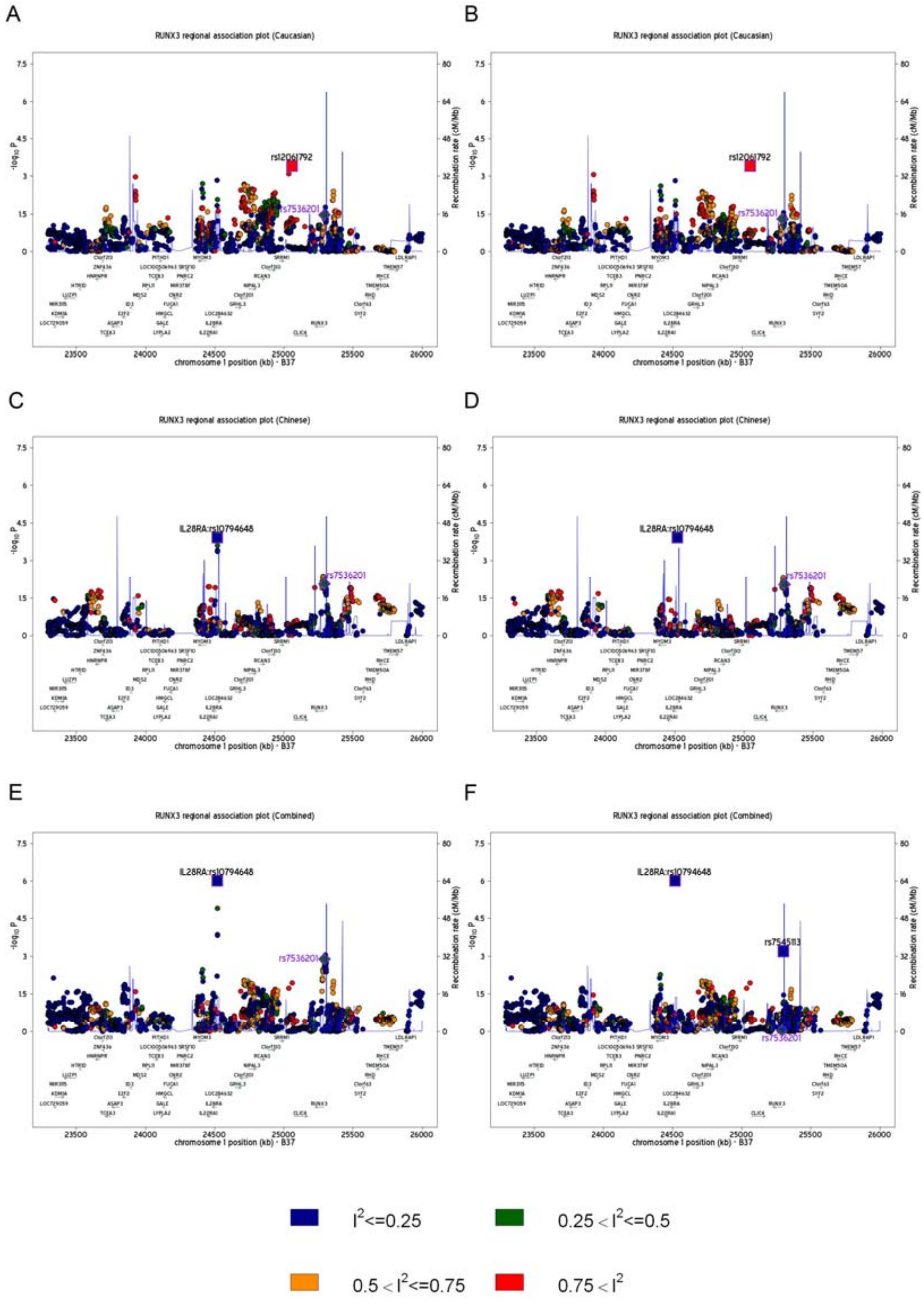
A
RUNX3 regional association plot (Caucasian)
rs12061792
rs7536201
B
RUNX3 regional association plot (Caucasian)
rs12061792
rs7536201
C
RUNX3 regional association plot (Chinese)
IL28RA:rs10794648
rs7536201
D
RUNX3 regional association plot (Chinese)
IL28RA:rs10794648
rs7536201
E
RUNX3 regional association plot (Combined)
IL28RA:rs10794648
rs7536201
F
RUNX3 regional association plot (Combined)
IL28RA:rs10794648
rs7545113
rs7536201
chromosome 1 position (kb) - B37
Recombination rate (cM/Mb)
I2<=0.25
0.25 < I2<=0.5
0.5 < I2<=0.75
0.75 < I2

w. RUNX3

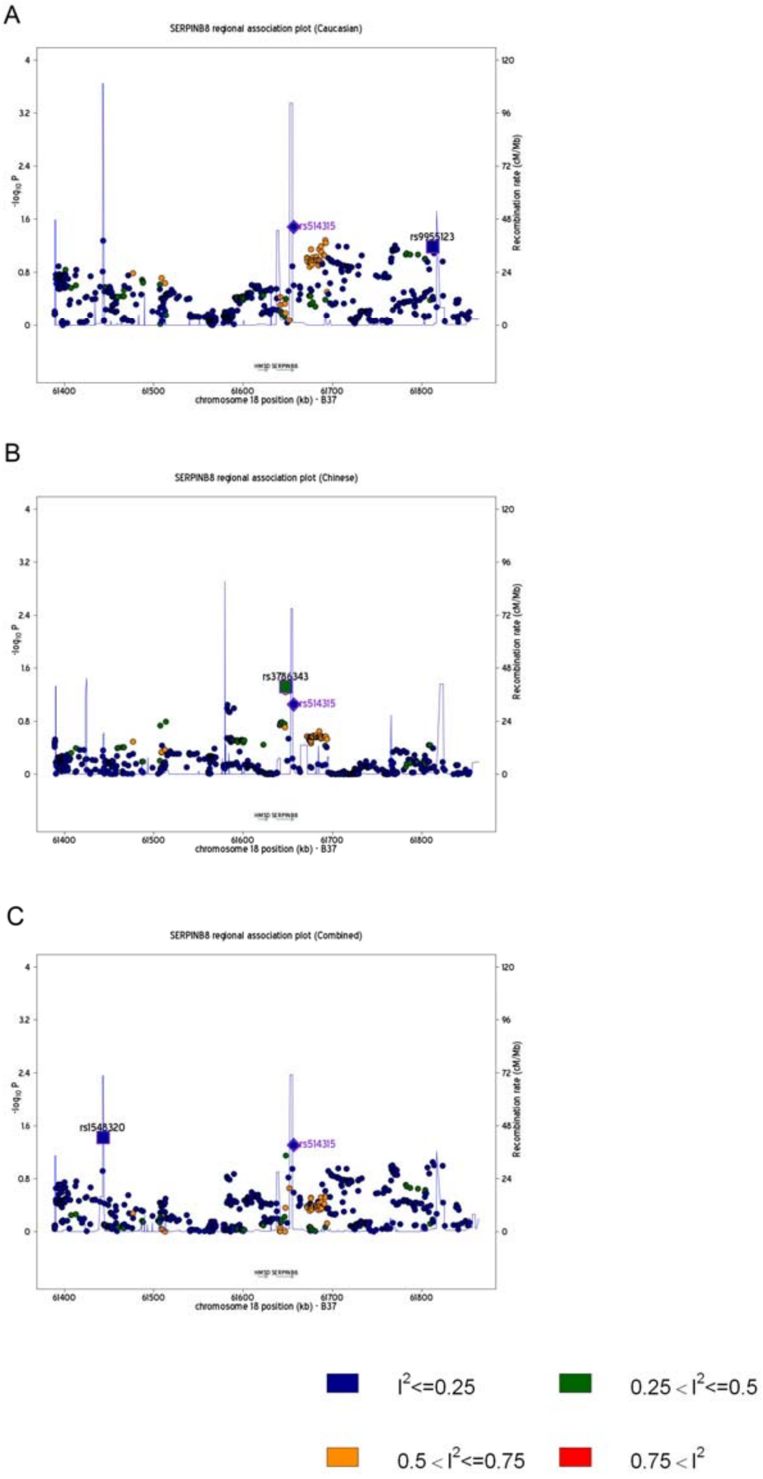
A
SERPINB8 regional association plot (Caucasian)
rs514315
rs9955123
B
SERPINB8 regional association plot (Chinese)
rs3786343
rs514315
C
SERPINB8 regional association plot (Combined)
rs1548320
rs514315
-log10 P
Recombination rate (cM/Mb)
chromosome 18 position (kb) - B37
61400
61500
61600
61700
61800
I2<=0.25
0.25 < I2<=0.5
0.5 < I2<=0.75
0.75 < I2

x. SERPINB8

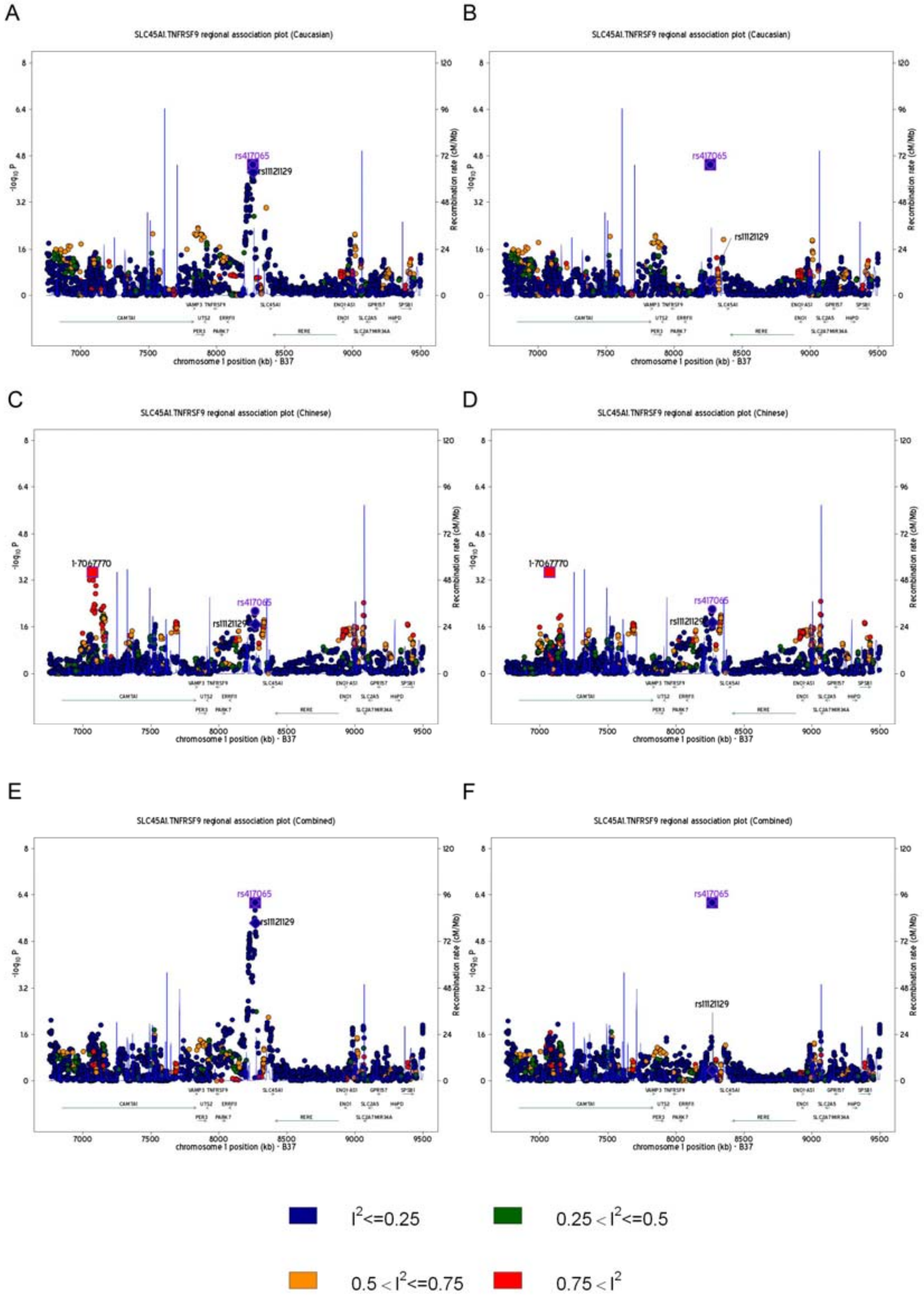

y. SLC45A1.TNFRSF9

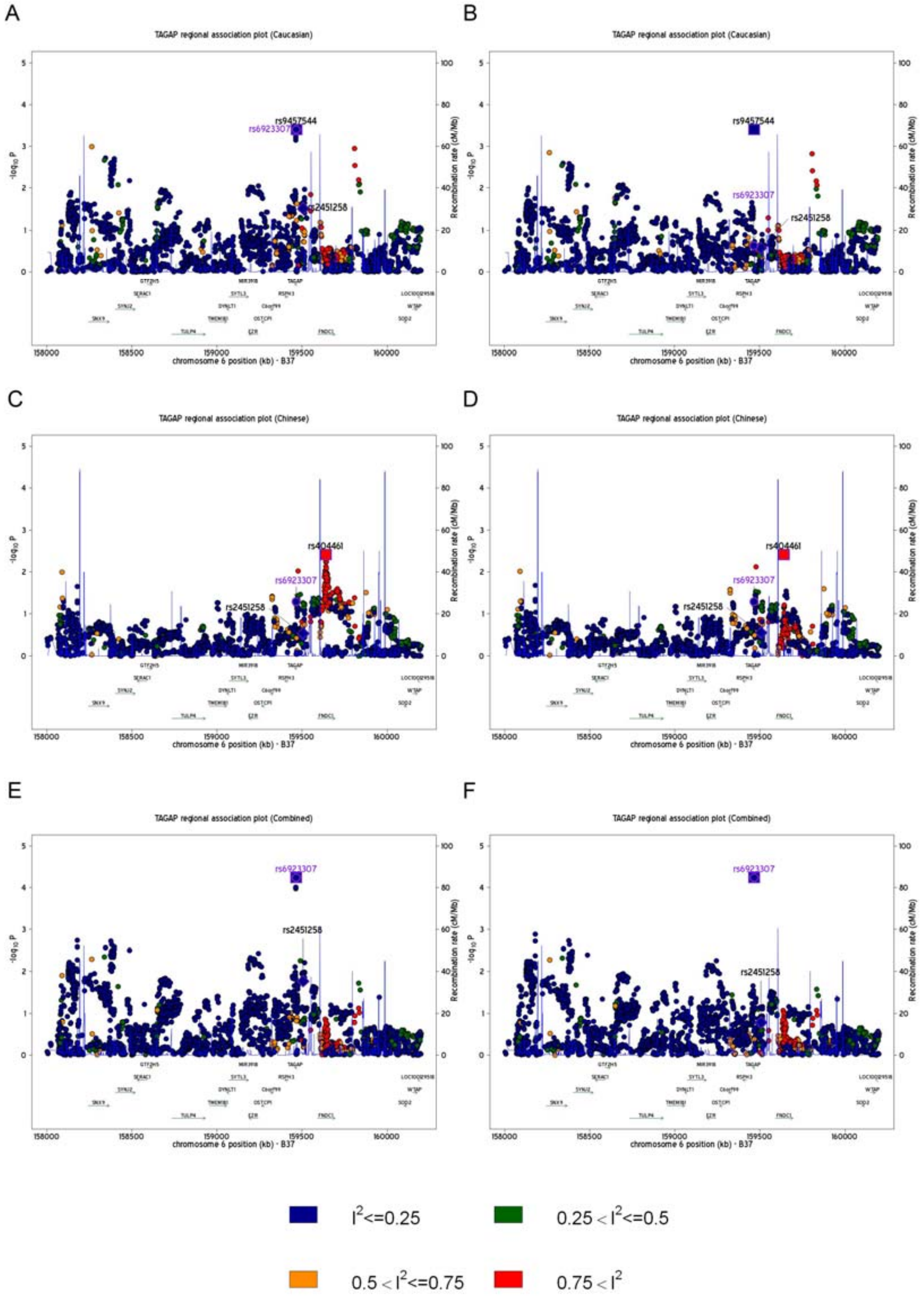
A
TAGAP regional association plot (Caucasian)
rs9457544
rs6923307
rs2451258
B
TAGAP regional association plot (Caucasian)
rs9457544
rs6923307
rs2451258
C
TAGAP regional association plot (Chinese)
rs404461
rs6923307
rs2451258
D
TAGAP regional association plot (Chinese)
rs404461
rs6923307
rs2451258
E
TAGAP regional association plot (Combined)
rs6923307
rs2451258
F
TAGAP regional association plot (Combined)
rs6923307
rs2451258
-log10 P
Recombination rate (cM/Mb)
chromosome 6 position (kb) - B37
I2<=0.25
0.25 < I2<=0.5
0.5 < I2<=0.75
0.75 < I2

z. TAGAP

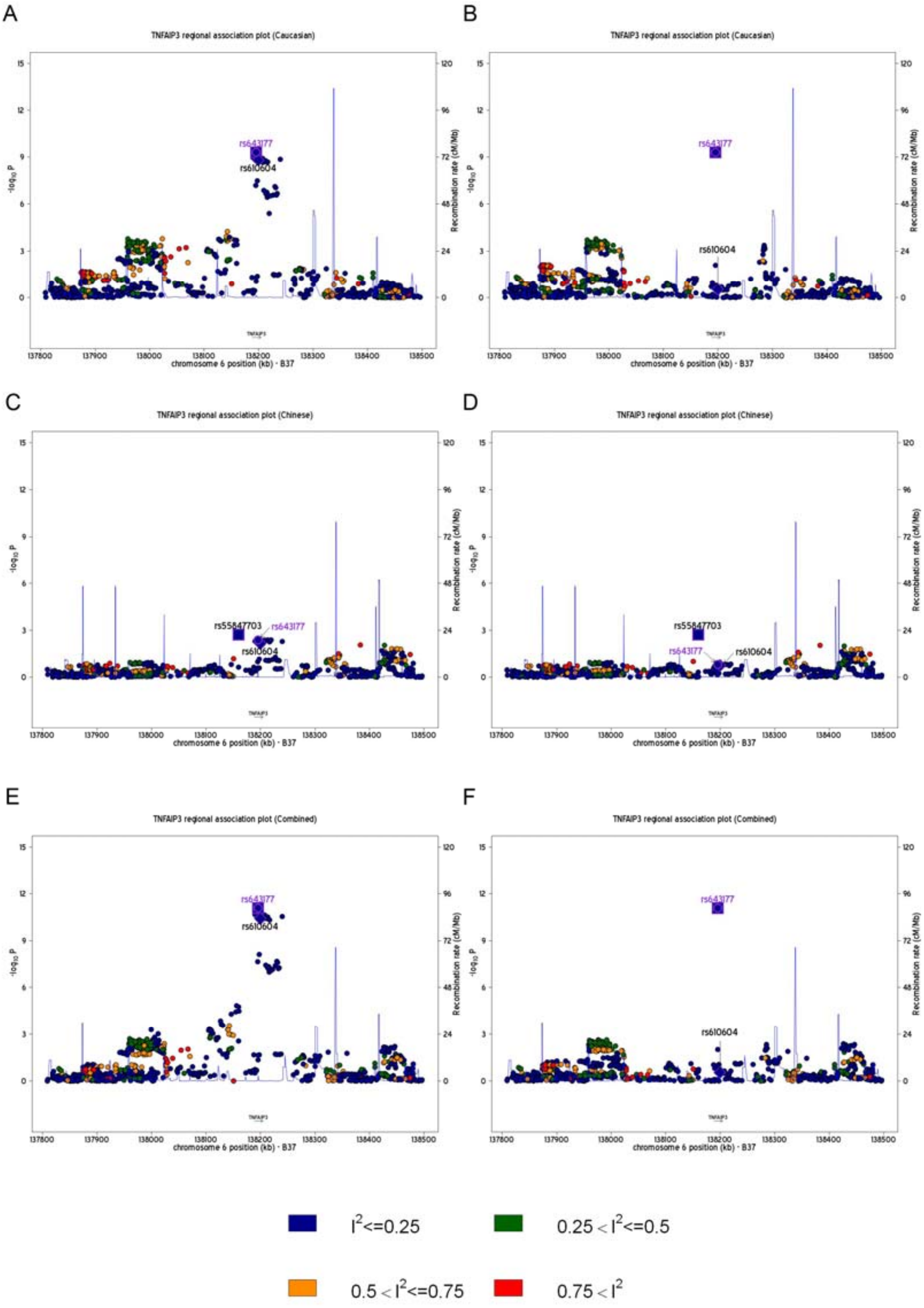
A
TNFAIP3 regional association plot (Caucasian)
rs643177
rs610604
B
TNFAIP3 regional association plot (Caucasian)
rs643177
rs610604
C
TNFAIP3 regional association plot (Chinese)
rs55847703
rs643177
rs610604
D
TNFAIP3 regional association plot (Chinese)
rs55847703
rs643177
rs610604
E
TNFAIP3 regional association plot (Combined)
rs643177
rs610604
F
TNFAIP3 regional association plot (Combined)
rs643177
rs610604
-log10 P
Recombination rate (cM/Mb)
chromosome 6 position (kb) - B37
TNFAIP3
I2<=0.25
0.25 < I2<=0.5
0.5 < I2<=0.75
0.75 < I2

aa. TNFAIP3

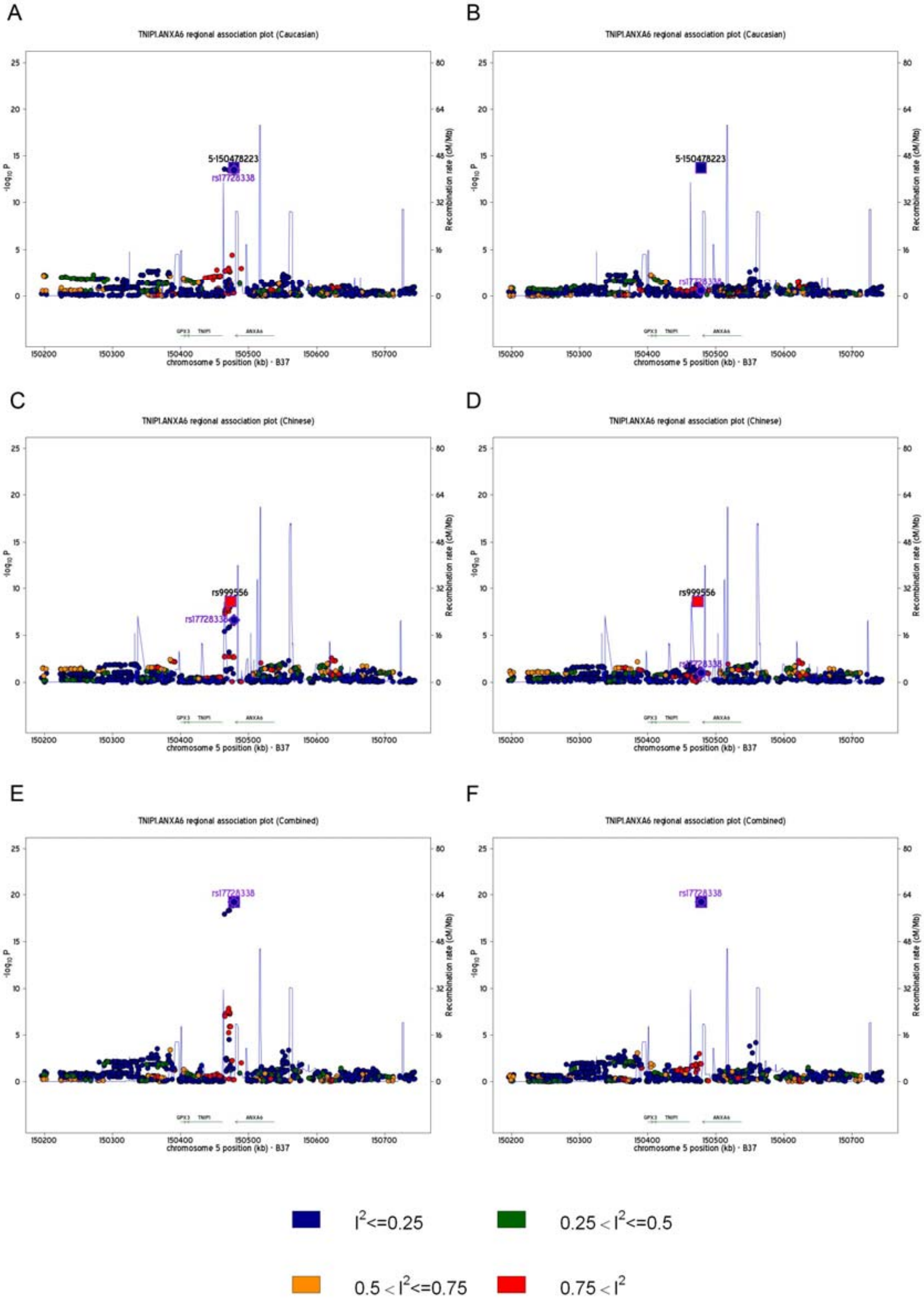

ab. TNIP1.ANXA6

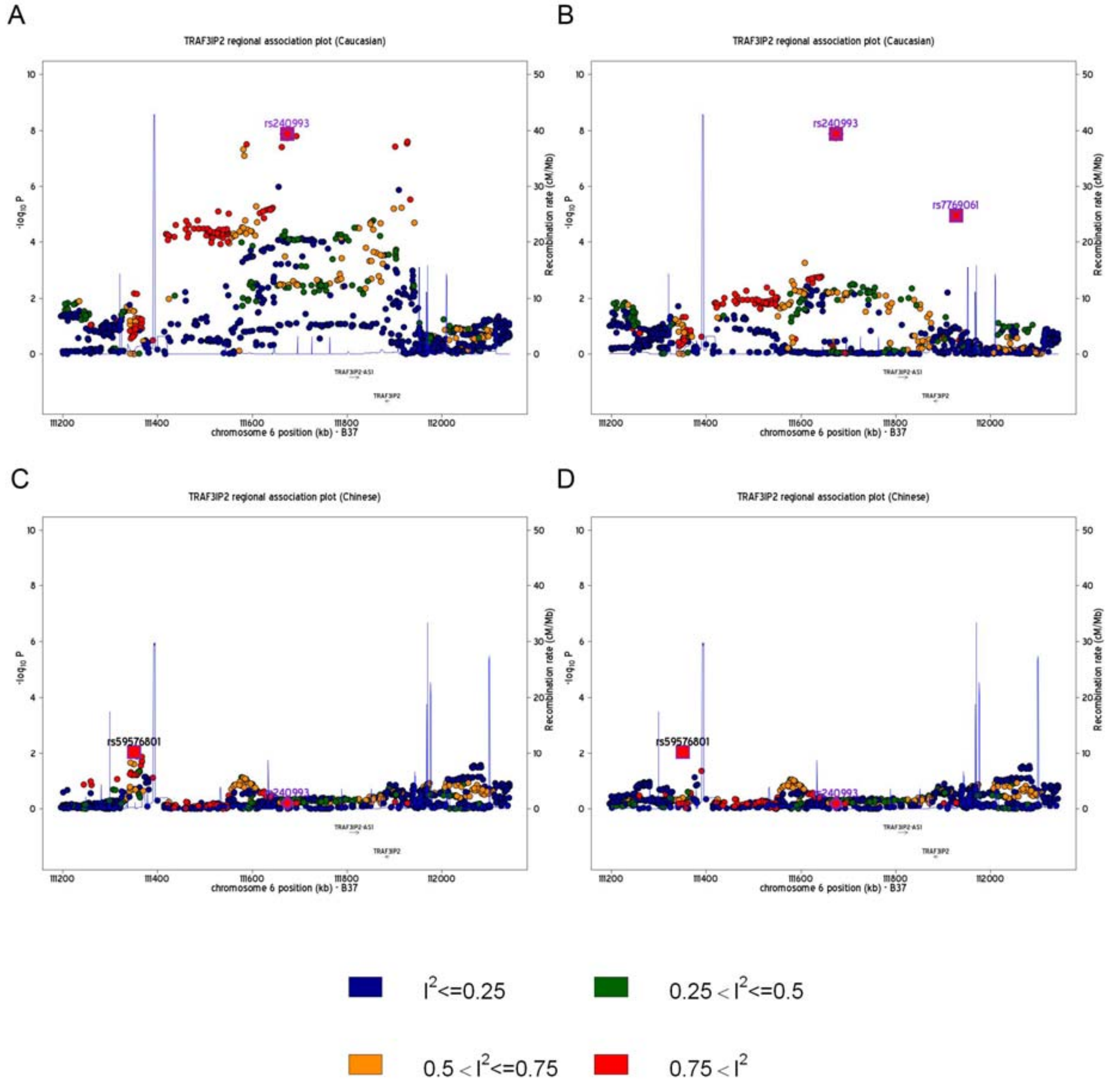

A
TRAF3IP2 regional association plot (Caucasian)
rs240993
B
TRAF3IP2 regional association plot (Caucasian)
rs240993
rs7769061
C
TRAF3IP2 regional association plot (Chinese)
rs59576801
D
TRAF3IP2 regional association plot (Chinese)
rs59576801
-log10 P
Recombination rate (cM/Mb)
chromosome 6 position (kb) - B37
TRAF3IP2-AS1
TRAF3IP2
I2<=0.25
0.25 < I2<=0.5
0.5 < I2<=0.75
0.75 < I2

ac. TRAF3IP2

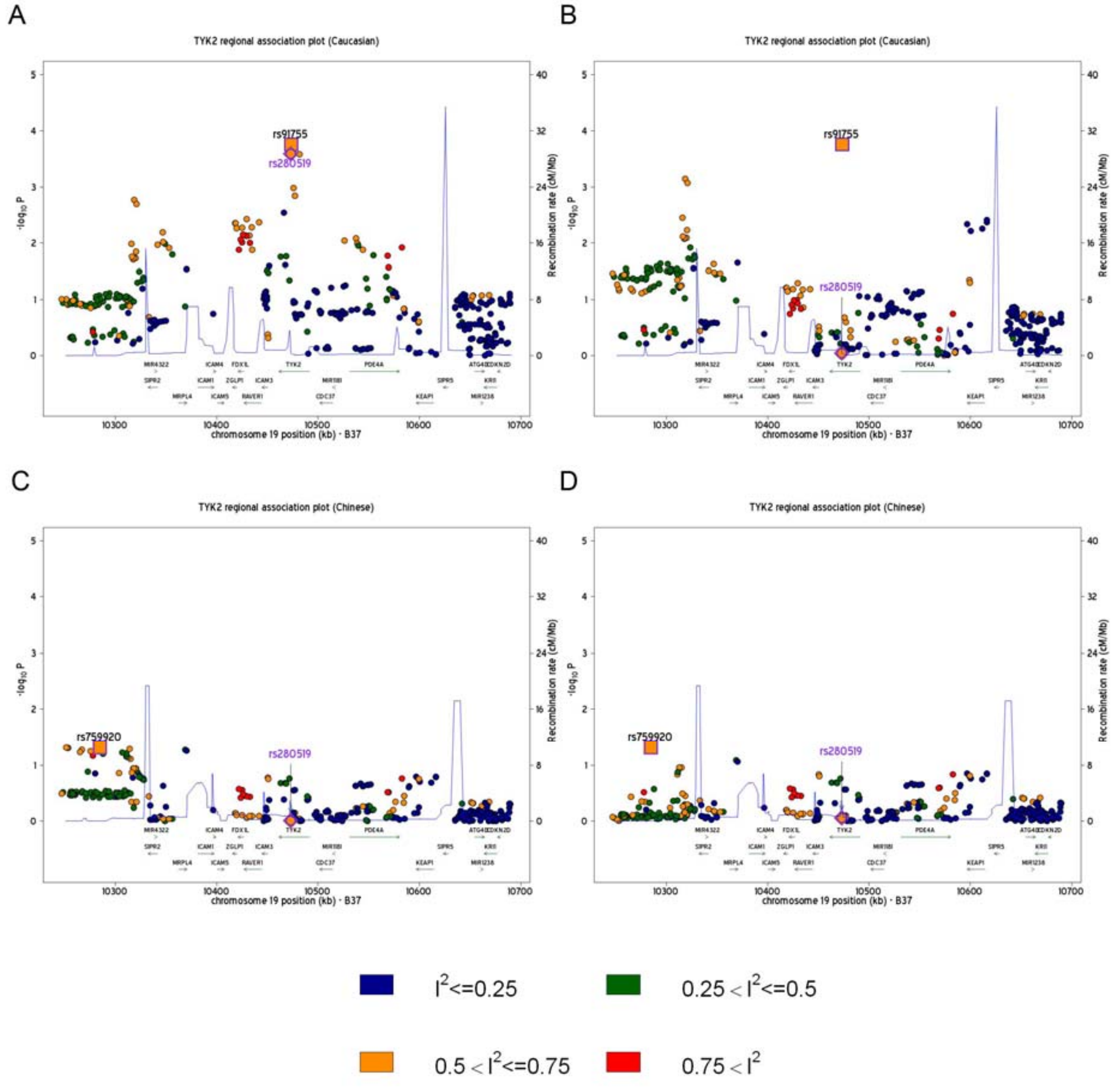
A
TYK2 regional association plot (Caucasian)
rs91755
rs280519
B
TYK2 regional association plot (Caucasian)
rs91755
rs280519
C
TYK2 regional association plot (Chinese)
rs759920
rs280519
D
TYK2 regional association plot (Chinese)
rs759920
rs280519
-log10 P
Recombination rate (cM/Mb)
chromosome 19 position (kb) - B37
I2<=0.25
0.25 < I2<=0.5
0.5 < I2<=0.75
0.75 < I2

ad.TYK2

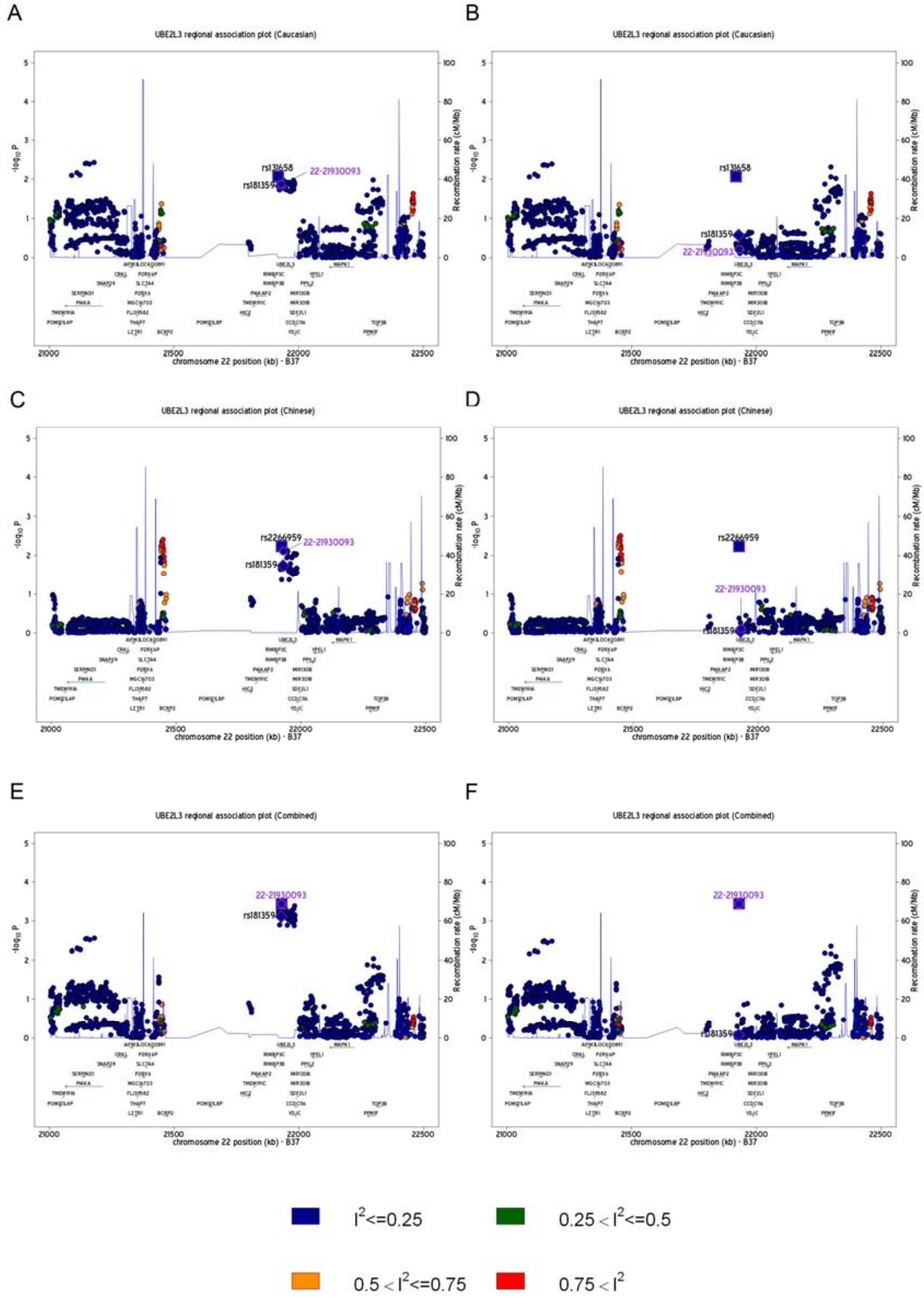
A
UBE2L3 regional association plot (Caucasian)
B
UBE2L3 regional association plot (Caucasian)
C
UBE2L3 regional association plot (Chinese)
D
UBE2L3 regional association plot (Chinese)
E
UBE2L3 regional association plot (Combined)
F
UBE2L3 regional association plot (Combined)
-log10 P
Recombination rate (cM/Mb)
chromosome 22 position (kb) - B37
rs131658
rs181359
rs2266959
22-21930093
I²<=0.25
0.25 < I²<=0.5
0.5 < I²<=0.75
0.75 < I²

ae. UBE2L3

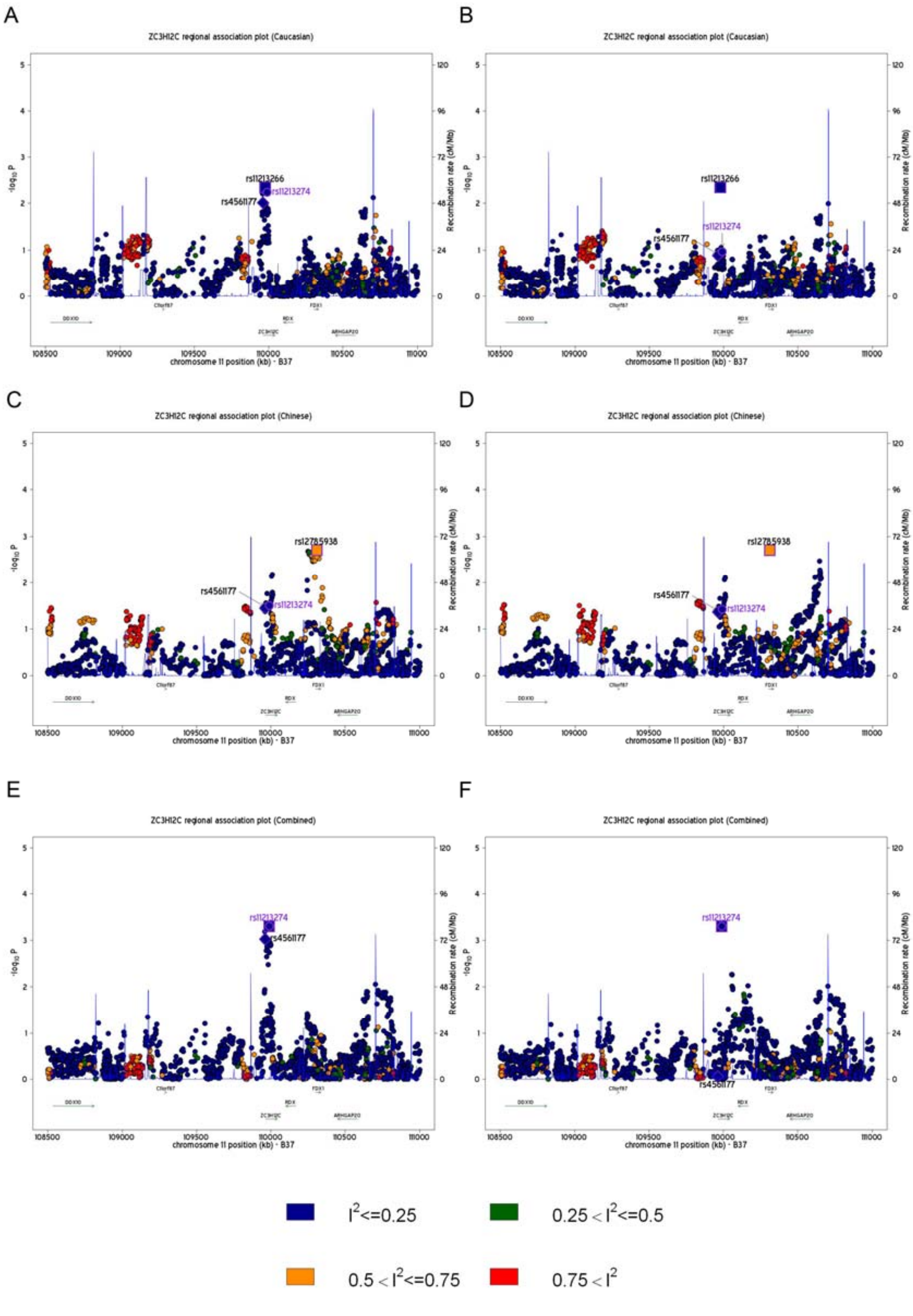
A
ZC3H12C regional association plot (Caucasian)
rs11213266
rs11213274
rs4561177
B
ZC3H12C regional association plot (Caucasian)
rs11213266
rs11213274
rs4561177
C
ZC3H12C regional association plot (Chinese)
rs12785938
rs4561177
rs11213274
D
ZC3H12C regional association plot (Chinese)
rs12785938
rs4561177
rs11213274
E
ZC3H12C regional association plot (Combined)
rs11213274
rs4561177
F
ZC3H12C regional association plot (Combined)
rs11213274
rs4561177
-log10 P
Recombination rate (cM/Mb)
chromosome 11 position (kb) - B37
DDX10
C11orf87
RDX
FDX1
ZC3H12C
ARHGAP20
I2<=0.25
0.25 < I2<=0.5
0.5 < I2<=0.75
0.75 < I2

af. ZC3H12C

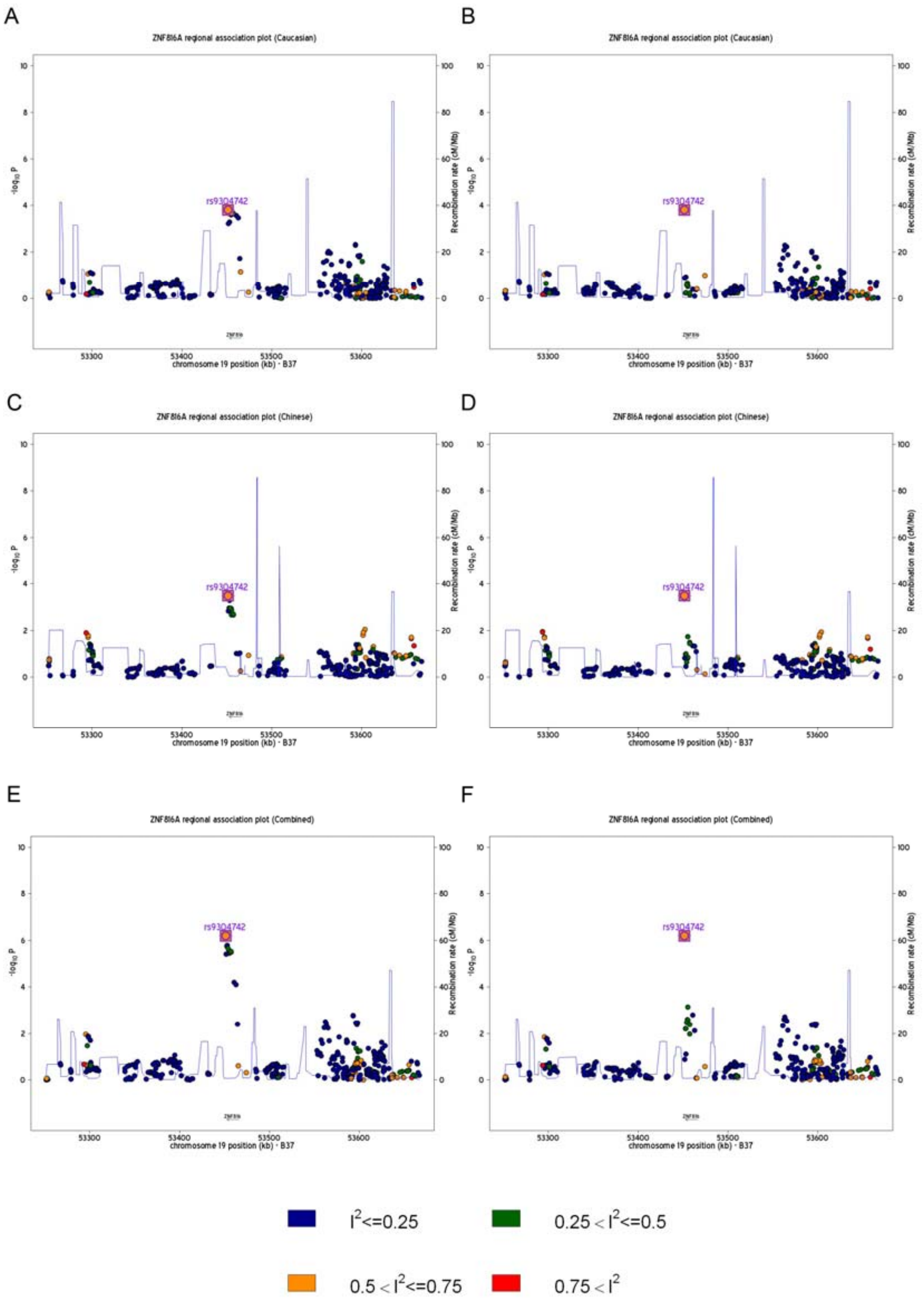
A
ZNF816A regional association plot (Caucasian)
B
ZNF816A regional association plot (Caucasian)
C
ZNF816A regional association plot (Chinese)
D
ZNF816A regional association plot (Chinese)
E
ZNF816A regional association plot (Combined)
F
ZNF816A regional association plot (Combined)
rs9304742
-log10 P
Recombination rate (cM/Mb)
chromosome 19 position (kb) - B37
53300
53400
53500
53600
I2<=0.25
0.25 < I2<=0.5
0.5 < I2<=0.75
0.75 < I2

ag. ZNF816A

Supplementary Figure 4: Regional association plot of 33 psoriasis risk loci. The relative location of annotated genes and the direction of transcription are shown in the lower portion of the figure, and the chromosomal position is shown on the *x* axis. The blue line shows the recombination rate (estimated from HapMap data of CEU, CHB+JPT and Combined population) across the region (right *y* axis), and the left *y* axis shows the significance of the associations. The log10 *P* values from the log-additive genetic model for all SNPs in the region from the population meta analysis, trans-ethnic meta analysis or the conditional analysis. A, C and E are unconditional association results of Caucasian, Chinese and combined GWAS meta-analysis respectively. B, D, F are conditional association results of Caucasian, Chinese and combined GWAS meta-analysis respectively. The purple diamond indicates the *P* value for the reported SNP; the purple square indicates the condition SNPs (these are the top or secondary SNPs), their unconditional P values are shown; the purple circle labeled with rs IDs in purple font are psoriasis susceptibility SNPs with statistics presented in the tables, they are in the same LD block with reported or condition SNPs which are in black font rs IDs; grey font rs IDs are secondary SNPs which were not validated in our studies. All circles, squares and diamonds are color filled according to the level of heterogeneity among the 2 populations (such as I^2).

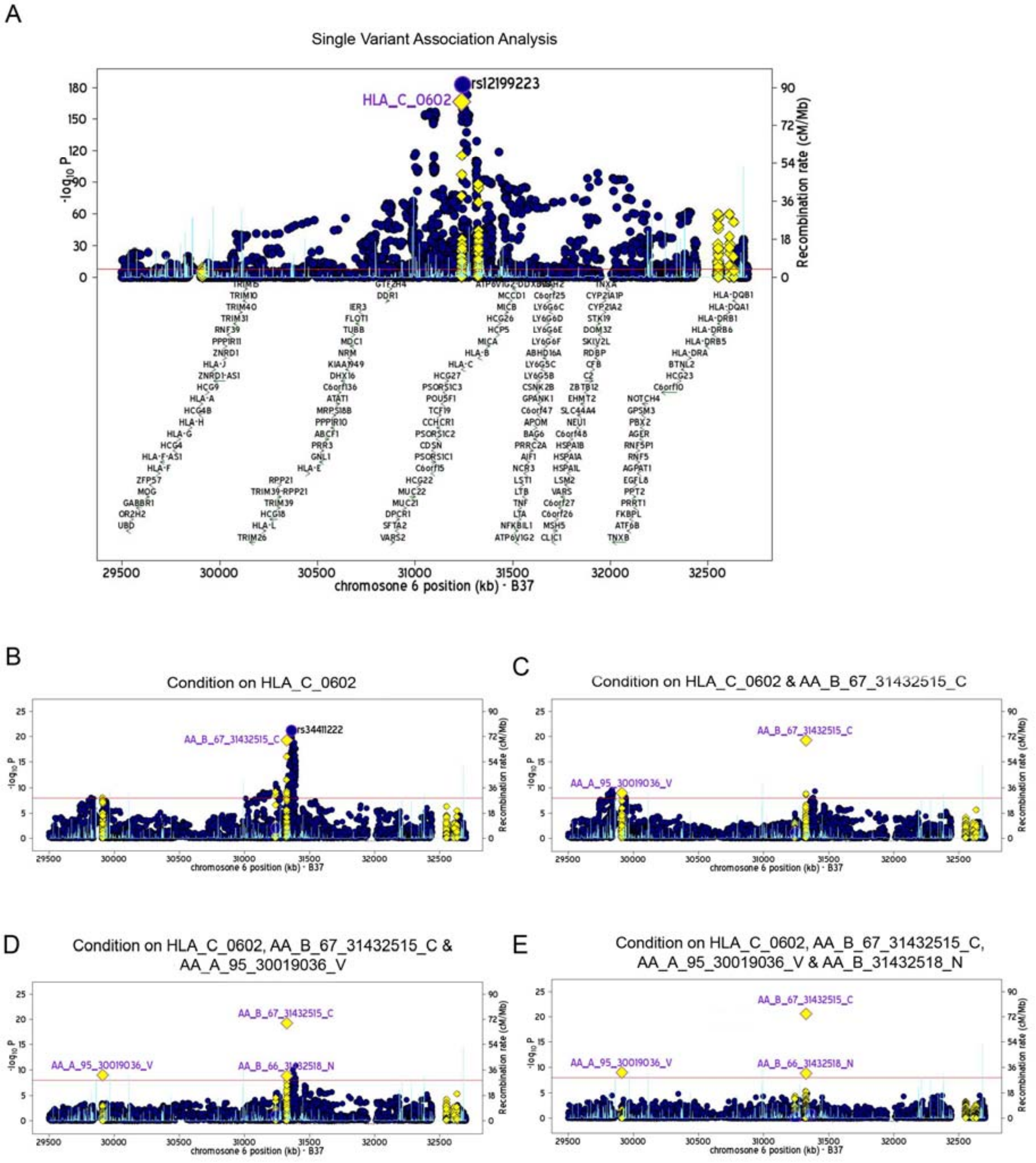

a. In the Caucasian GWAS cohort

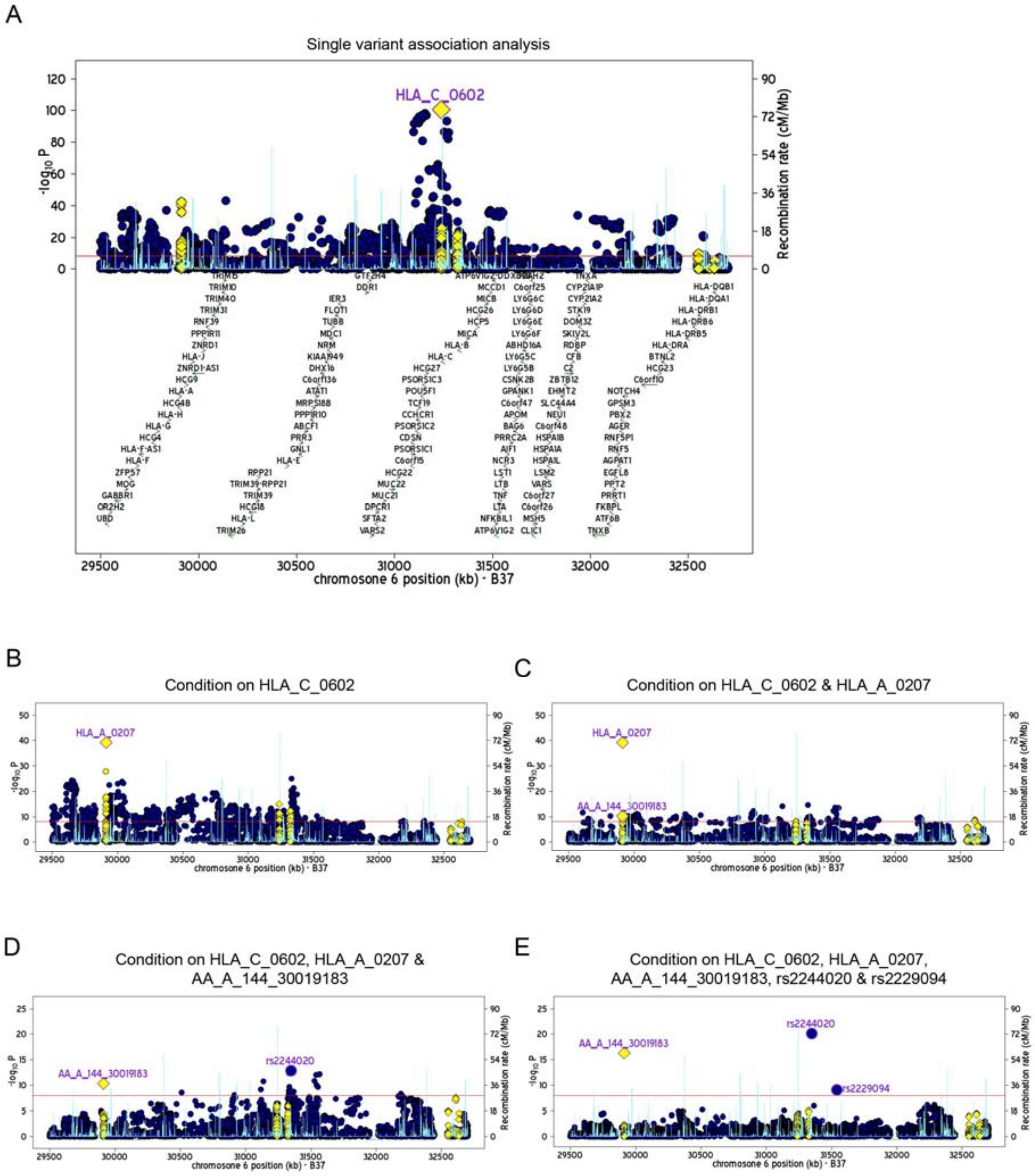

b. In the Chinese GWAS cohort

Supplementary Figure 5: Regional association plot of *HLA* region. The relative location of annotated genes and the direction of transcription are shown in the lower portion of the figure, and the chromosomal position is shown on the *x* axis. The green line shows the recombination rate (estimated from HapMap data of CEU, CHB+JPT and Combined population) across the region (right *y* axis), and the left *y* axis shows the significance of the associations. The log10 *P* values from the log-additive genetic model for all SNPs in the region from the population meta analysis or the conditional analysis. The horizontal red line indicates the genome-wide association significance criteria ($P=5\times10^{-8}$), a Bonferroni corrected type I error rate based on the analysis in the whole genome common markers. A is unconditional association results of Caucasian, Chinese respectively. B, C, D, E are conditional association results of Caucasian, Chinese respectively. The Yellow diamond indicates the *P* value for the reported classical alleles; The blue circle indicates the reported SNPs in the paper; rs IDs in black or purple font are psoriasis susceptibility SNPs with statistics presented in the tables. (a) In the Caucasian GWAS cohort. (b) In the Chinese GWAS cohort.

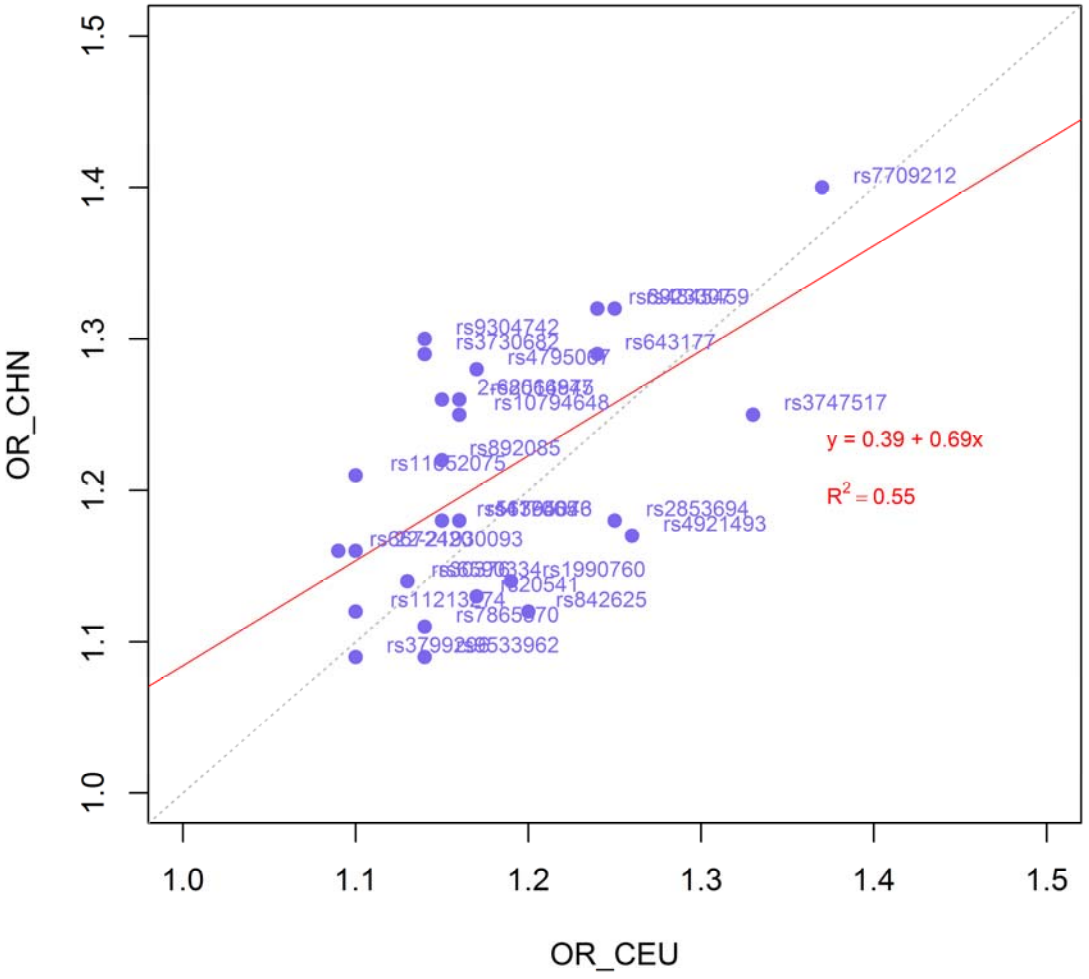

Supplementary Figure 6: The correlation of odds ratios for the 30 SNPs in 27 shared loci between European and Chinese populations in this study. The risk effect size (odds ratios, OR) frequencies of 30 SNPs in 27 shared loci between two ancestries were significantly correlated ($y = 0.39 + 0.69x$, $R^2 = 0.55$, $P = 1.49 \times 10^{-6}$), *P*-value from linear regression of odd ratios between in European and in Chinese populations.

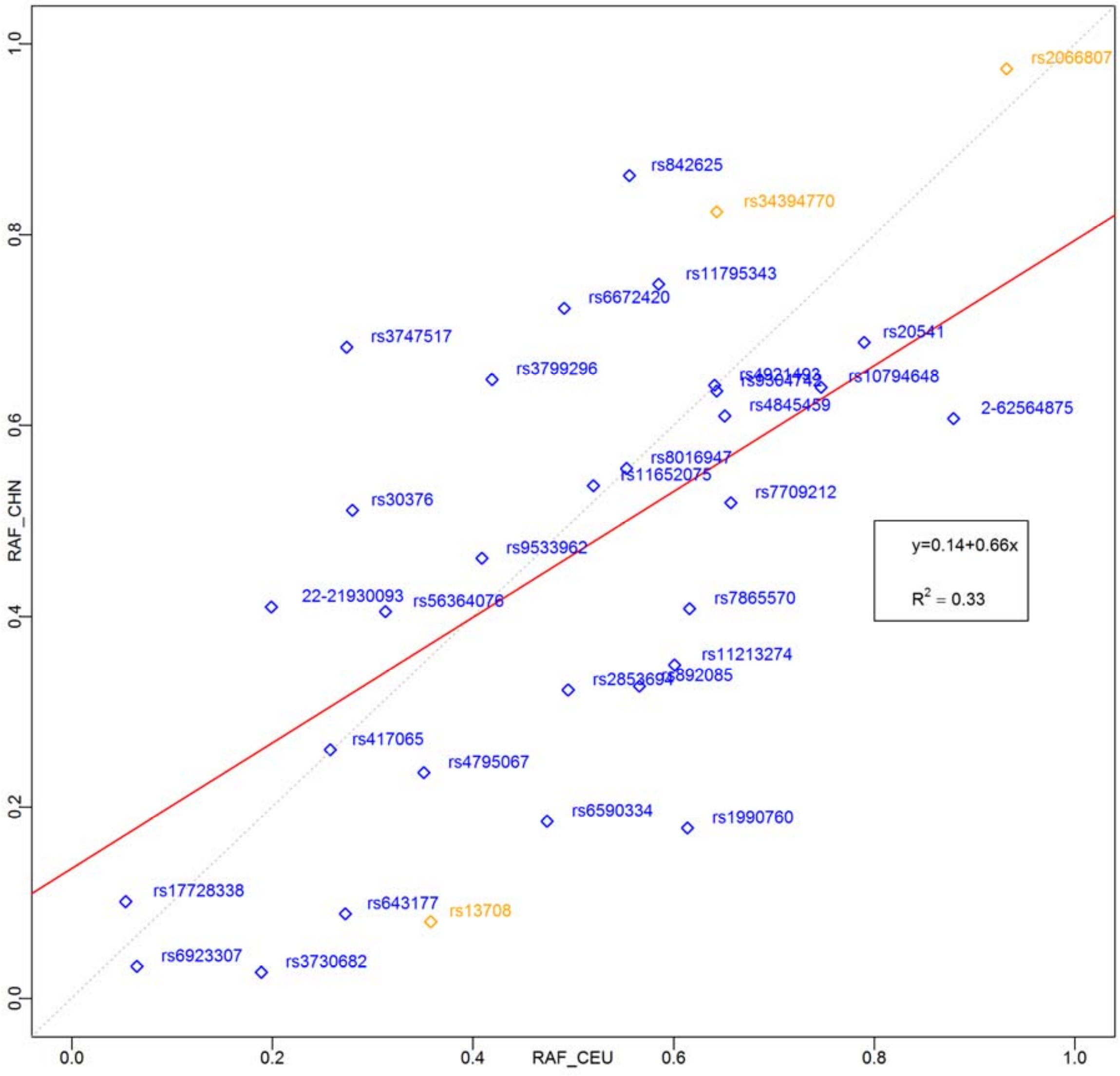

Supplementary Figure 7: The correlation of risk allele frequencies of 33 SNPs in 30 shared loci and between European and Chinese populations in this study. The three SNPs (rs13708, rs34394770 and rs2066807) with significant allele frequency heterogeneity were labelled in orange. The 30 SNPs in 27 shared loci were labelled in blue. The risk allele frequencies of 30 SNPs in 27 shared loci between two ancestries were significantly correlated (y = 0.14 + 0.66x, R^2 = 0.33, P = 0.0005), P-value from linear regression of risk alleles frequencies between in European and in Chinese populations.

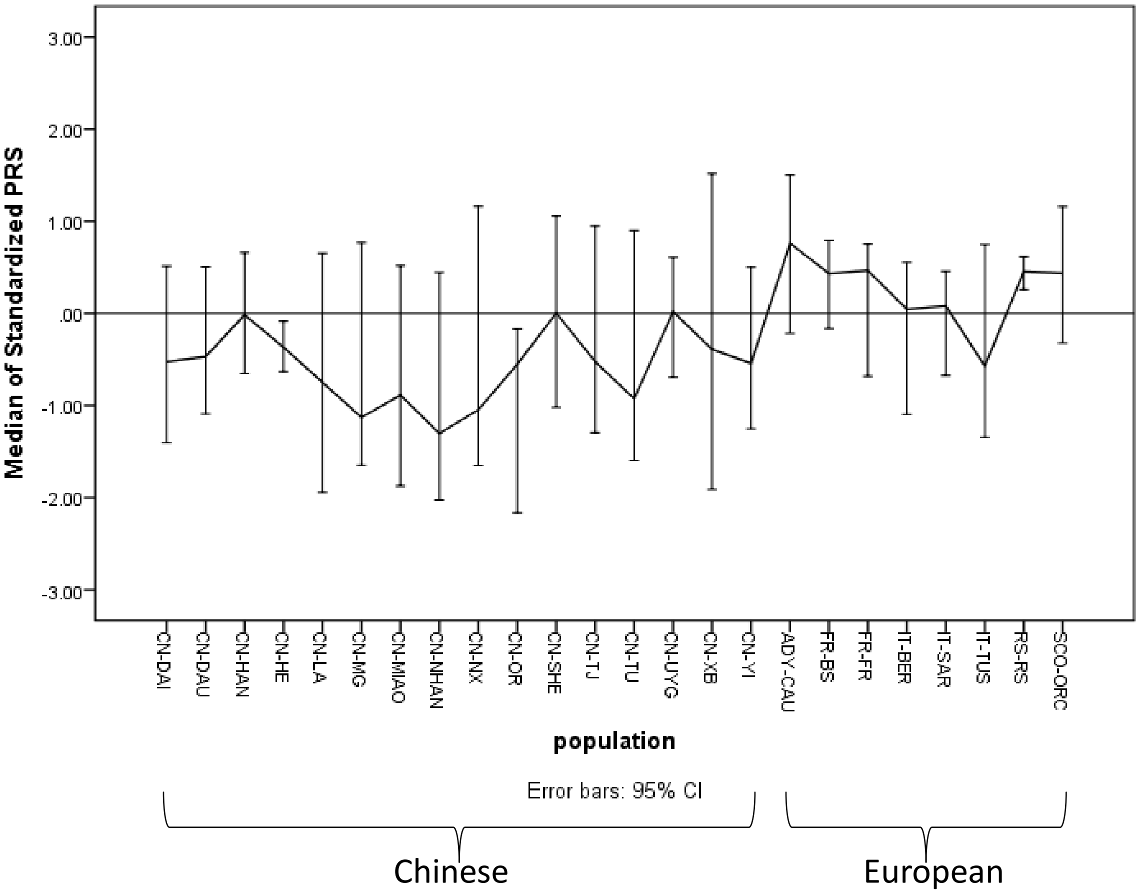

Supplementary Figure 8: The distribution of median of standardized polygenic risk score (PRS) among Chinese and European in HGDP. The polygenic risk score (PRS) was calculated as described (Online Method). The *x*-axis indicates the populations. The *y*-axis indicates the median value of standardized PRS. The horizontal line indicates that the median value of standardized PRS is zero. The error bars show the 95% confidential interval. All median value of standardized PRS are connected by a line. Standardized genetic risk score is quite different among 52 HGDP populations with *P* of 1.15E-09 from Kruskal-Wallis test (non-parametric test). And the risk score is negatively correlated with longitude (*P*=3.23E-07), however not related to latitude of populations (*P*=0.053) from the spearman's rank correlation test. The Figure shows that the risk score is lower in Chinese than in European.

Supplementary Table 1: Power calculation in Chinese sample. The statistical power was as a function of disease allele frequency and genotype relation risk for the actual Chinese sample size used in our study. The power was calculated by QUANTO.

	Genotype Relation Risks(Log-Additive Model)						
Disease Allele Frequency	1.10	1.15	1.20	1.25	1.30	1.35	1.40
0.10	0.18%	4.53%	30.62%	74.19%	96.21%	99.80%	99.99%
0.20	1.87%	31.65%	85.90%	99.51%	99.99%	99.99%	99.99%
0.30	5.37%	58.10%	97.46%	99.99%	99.99%	99.99%	99.99%
0.40	8.61%	70.90%	99.15%	99.99%	99.99%	99.99%	99.99%
0.50	9.66%	73.57%	99.33%	99.99%	99.99%	99.99%	99.99%

Supplementary Table 2: The functional annotation of novel SNPs in ENCODE through HaploReg v2

SNP	chr	POS	r²	D'	Proxy SNPs	Ref	Alt	Enhancer histone marks	DNAse	Proteins bound
rs28512356	3	189615475	1	1	rs28512356	C	A	NHEK, HMEC	SAEC	GR,NRSF,POL2B
rs28512356	3	189617088	0.98	1	rs71298526	GA	G	NHEK, HMEC	NHEK,HEEpiC,SAEC	
rs28512356	3	189617276	1	1	rs28688563	G	T	NHEK, HMEC		
rs28512356	3	189623672	1	1	rs13081982	A	T			
rs28512356	3	189627940	0.98	0.99	rs36006475	TA	T	NHEK, HMEC, Huvec		
rs28512356	3	189637419	0.98	0.99	rs12485364	A	G			
rs28512356	3	189638198	0.83	0.99	rs13065424	G	A			
rs28512356	3	189638887	0.94	0.98	rs35822139	C	A			
rs34394770	13	40299842	0.95	0.99	rs35623045	ATAG	A			
rs34394770	13	40300328	0.89	0.99	rs9603603	T	G			
rs34394770	13	40308307	0.97	0.99	rs9603605	T	C			
rs34394770	13	40318819	0.99	1	rs9603608	A	C			
rs34394770	13	40319225	0.86	-0.99	rs144565908	T	A			
rs34394770	13	40319954	0.99	1	rs28635831	A	G			
rs34394770	13	40326282	0.98	1	rs9943	A	G			
rs34394770	13	40333369	1	1	rs34394770	T	C			
rs34394770	13	40334852	0.99	1	rs9603612	C	G		GM18507,GM19238	EBF1
rs34394770	13	40336770	0.98	1	rs60120504	AC	A			
rs34394770	13	40342557	0.99	1	rs12875311	G	A	H1	H1-hESC,H7-hESC	
rs34394770	13	40345356	0.99	1	rs7319041	G	A			
rs34394770	13	40345466	0.98	0.99	rs7320598	C	T			
rs34394770	13	40350912	0.99	-0.99	rs7993214	T	C	5 cell types	56 cell types	NFKB,CFOS,P300
rs34394770	13	40351064	0.91	-0.99	rs7986796	G	T	5 cell types	HRGEC	
rs34394770	13	40351094	0.99	0.99	rs12876235	T	G	5 cell types	HRGEC	
rs34394770	13	40351341	0.99	-0.99	rs9532430	A	G	6 cell types	HAEpiC	MAFK
rs34394770	13	40352671	0.99	-0.99	rs9532433	C	A	Huvec		
rs34394770	13	40353230	0.99	-0.99	rs9548932	G	T	Huvec, GM12878		

rs34394770	13	40354200	0.99	0.99	rs12872801	C	T	HSMM, Huvec	15 cell types	
rs34394770	13	40355913	0.99	-0.99	rs9532434	T	C			
rs34394770	13	40362229	0.97	0.99	rs8002731	A	C	HSMM, Huvec, H1		MAX
rs34394770	13	40368069	0.89	0.96	rs9603616	C	T			
rs34394770	13	40368444	0.87	0.95	rs11618582	A	G			
rs34394770	13	40368601	0.89	0.96	rs9594366	C	T			
rs9533962	13	45321621	0.92	0.97	rs7982911	G	A	HMEC, NHEK	AG09309	
rs9533962	13	45334194	1	1	rs9533962	C	T	6 cell types	15 cell types	FOXA1
rs9533962	13	45342453	0.94	0.98	rs1415641	C	T	4 cell types		NFKB
rs9533962	13	45342796	0.95	0.98	rs1415640	G	A	4 cell types		
rs9533962	13	45343590	0.95	0.98	rs9526002	A	G	HMEC, NHEK, GM12878	26 cell types	5 bound proteins
rs9533962	13	45355541	0.85	0.98	rs9533966	A	G		Fibrobl	
rs2853694	21	36470865	1	1	rs8128234	C	T	K562		
rs2853694	2	163128824	1	1	rs3747517	T	C			
rs2853694	5	158730368	1	1	rs4921464	A	G			SETDB1
rs2853694	5	158731548	1	1	rs1549922	G	A			
rs2853694	5	158733619	0.94	1	rs4921213	G	A			
rs2853694	5	158734517	1	1	rs11135058	T	C		HeLa-S3	
rs2853694	5	158749088	1	1	rs2853694	G	T			
rs4921493	5	158833535	0.93	0.98	rs12374547	G	A	GM12878		
rs4921493	5	158834367	0.93	0.98	rs60689680	G	T			
rs4921493	5	158835178	0.87	0.99	rs200786876	TTTTTC	T			
rs4921493	5	158835179	0.92	0.99	rs10556150	TTTTC	T			
rs4921493	5	158836107	1	1	rs4921493	T	C			
rs4921493	5	158836126	0.94	0.99	rs4921494	G	A			
rs4921493	5	158836581	0.94	1	rs12655757	C	T	GM12878		
rs4921493	5	158839217	0.98	1	rs1422878	C	T	GM12878		
rs4921493	5	158839512	0.99	1	rs1422877	A	G	H1, GM12878	21 cell types	NFKB,CEBPB,STAT3
rs4921493	5	158839745	0.99	1	rs12651787	T	C	GM12878, H1		EBF1
rs4921493	5	158841470	0.96	0.99	rs10476296	C	T	GM12878	GM06990	
rs4921493	5	158843235	0.89	0.96	rs6863015	C	A			
rs4921493	5	158843253	0.86	0.96	rs6888061	T	C			
rs4921493	5	158844232	0.92	0.97	rs58368531	G	A	K562		
rs4921493	5	158846606	0.87	0.96	rs72802188	C	T			CTCF
rs4921493	5	158848253	0.87	0.96	rs4921497	C	G			
rs4921493	5	158848433	0.86	0.95	rs4921498	G	A			
rs4921493	5	158849200	0.86	0.95	rs11743870	G	A			

rs4921493	5	158849636	0.86	0.95	rs10155584	G	A		5 cell types	
rs4921493	5	158849837	0.86	0.95	rs4921227	A	G			
rs4921493	5	158852944	0.85	0.95	rs7714401	T	A	GM12878	FibroP	EBF1
rs4921493	5	158853941	0.85	0.95	rs2012830	A	T	GM12878	GM12878,GM12864,GM12865	BATF
rs4921493	5	158856506	0.83	0.93	rs4921501	C	A			
rs4921493	5	158856513	0.83	0.94	rs4921228	C	A			

SNP: the reported novel SNP in our study; chr.: chromosome; Pos: physical position in HG19. r2 and D' indicate the linkage status between SNP and proxy SNPs.

Supplementary Table 3: The known eQTL effect at the four novel loci

GWAS SNP	Linked SNP	r^2	D'	Tissue	P-value	Gene
rs34394770	rs4514547	0.7	0.88	Monocytes	9.50E-07	COG6
rs34394770	s9943	0.934	1	Monocytes	3.61E-05	COG6
rs34394770	rs12875311	0.934	1	Monocytes	3.36E-05	COG6
rs34394770	rs7993214	0.975	1	peripheral blood	7.11E-06	KIF18A

GWAS SNP: the novel reported SNPs in our study; Linked SNP: the SNPs with high linkage disequilibrium with reported SNPs. r^2 and D' indicate the linkage pair-wise status.
P-value indicates the association between the SNP and the respective gene transcript.

Supplementary Table 4a: Conditional association results of *IL12B* gene region

Analysis	rsid	Chr	BP.B37	RA	RAF		Caucasian GWAS		Chinese GWAS		Combined All		
					EUR	CHN	P	OR (95% CI)	P	OR (95% CI)	P	OR (95% CI)	p_Q
**Unconditional**	rs7709212	5	158764177	T	0.657	0.519	2.83E-20	1.37(1.31-1.44)	1.03E-11	1.4(1.31-1.5)	1.20E-30	1.38(1.33-1.44)	7.03E-01
	rs4921493	5	158836107	T	0.641	0.642	3.89E-13	1.28(1.21-1.34)	6.89E-05	1.24(1.13-1.34)	2.22E-16	1.27(1.21-1.32)	6.27E-01
	rs2853694	5	158749088	G	0.495	0.323	2.98E-22	1.36(1.3-1.42)	2.12E-09	1.37(1.27-1.48)	8.61E-30	1.36(1.31-1.42)	8.87E-01
**Conditioning on rs7709212**	rs4921493	5	158836107	T	0.641	0.642	2.70E-11	1.26(1.19-1.32)	4.66E-03	1.17(1.06-1.28)	6.80E-13	1.23(1.17-1.29)	2.78E-01
	rs2853694	5	158749088	G	0.495	0.323	7.74E-08	1.23(1.16-1.31)	4.48E-03	1.19(1.07-1.31)	1.22E-09	1.22(1.16-1.28)	5.87E-01
**Conditioning on rs7709212 & rs4921493**	rs2853694	5	158749088	G	0.495	0.323	1.91E-08	1.25(1.17-1.32)	5.24E-03	1.18(1.07-1.3)	3.79E-10	1.23(1.16-1.29)	4.76E-01
**Multivariate analysis**	rs7709212	5	158764177	T	0.657	0.519	5.92E-05	1.19(1.09-1.29)	8.04E-05	1.26(1.12-1.42)	3.35E-08	1.21(1.13-1.3)	5.38E-01
	rs4921493	5	158836107	T	0.641	0.642	8.14E-12	1.26(1.18-1.35)	6.19E-03	1.16(1.04-1.3)	3.52E-13	1.23(1.17-1.31)	3.92E-01
	rs2853694	5	158749088	G	0.495	0.323	1.93E-08	1.25(1.15-1.34)	3.82E-03	1.19(1.06-1.34)	4.24E-10	1.23(1.15-1.31)	2.12E-01

Chr: chromosome; BP.B37: position based on NCBI build 37; RA: risk/non-risk alleles; RAF: risk allele's frequencies; EUR: European samples; CHN: Chinese samples; *P*: association *P* values in the meta, trans ethnic meta analysis or the conditional study (alleles dosages in GWAS studies); OR: odds ratio; 95% CI: 95% confidence interval; p_Q: heterogeneity P values.

There were three independent loci identified: rs7709212, rs4921493, rs2853694.

Supplementary Table 4b: The pairwise linkage disequilibrium among the three SNPs in *IL12B* locus

	rs4921493		rs2853694	
**SNP**	**CEU**	**JPT+CHB**	**CEU**	**JPT+CHB**
rs7709212	r^2=0.019,D'=0.206	r^2=0.033,D'=0.209	r^2=0.499,D'=0.854	r^2=0.269,D'=0.892
rs2853694	r^2=0.024,D'=0.19	r^2=0.008,D'=0.103	/	/

* The Linkage disequilibrium was calculated based on the samples from CEU population and JPT, CHB populations in HapMap.

Supplementary Table 4c: Haplotypic association for markers in *IL12B* locus with the risk of psoriasis

				European GWAS				Chinese GWAS				Combined		
**Haplotype**	**rs2853694**	**rs7709212**	**rs4921493**	**Freq_contrl**	**Freq_case**	**OR (95% CI)**	**P**	**Freq_contrl**	**Freq_case**	**OR (95% CI)**	**P**	**OR (95% CI)**	**P**	**p_Q**
1	T	T	T	15.44%	15.37%	-	-	16.51%	20.16%	-	-	-	-	-
2	G	C	C	1.69%	1.33%	0.83(0.63-1.08)	1.72E-01	0.58%	0.81%	1.56(0.88-2.76)	1.28E-01	0.93(0.73-1.2)	5.54E-01	5.02E-02
3	G	C	T	0.92%	1.10%	1.15(0.84-1.57)	3.91E-01	1.79%	1.58%	0.82(0.55-1.22)	3.22E-01	1.01(0.79-1.3)	9.47E-01	1.91E-01
4	G	T	C	14.36%	14.76%	1.05(0.94-1.17)	4.08E-01	4.58%	4.72%	0.88(0.68-1.14)	3.30E-01	1.02(0.92-1.1)	7.14E-01	2.21E-01
5	G	T	T	32.66%	39.80%	1.23(1.12-1.35)	2.04E-05	25.21%	32.49%	1.17(1.01-1.36)	3.13E-02	1.21(1.12-1.3)	2.07E-06	6.08E-01
6	T	C	C	16.10%	11.71%	0.73(0.65-0.81)	5.64E-08	24.00%	18.35%	0.7(0.6-0.82)	1.19E-05	0.72(0.65-0.8)	3.21E-12	7.39E-01
7	T	C	T	16.01%	14.00%	0.88(0.78-0.98)	2.28E-02	21.76%	18.18%	0.78(0.67-0.92)	2.85E-03	0.85(0.77-0.9)	3.40E-04	2.64E-01
8	T	T	C	2.83%	1.92%	0.68(0.55-0.85)	7.08E-04	5.57%	6.30%	0.92(0.73-1.17)	5.03E-01	0.79(0.67-0.9)	3.56E-03	6.43E-02

Haplotype 1 was set as reference. Freq_contrl/Freq_cases : the haplotype frequency in controls/cases; P: the association P values for each haplotype in the specified population ; OR: odds ratio; 95% CI: 95% confidence interval; P_Q: heterogeneity P values.

Supplementary Table 5a: Conditional association results of *IFIH1* gene region

Analysis	rsid	Chr	BP.B37	RA	ARF		Combined European		Combined Chinese		Combined All		
					EUR	CHN	P	OR (95% CI)	P	OR (95% CI)	P	OR (95% CI)	p_Q
**Unconditional**	rs1990760	2	163124051	T	0.614	0.188	3.21E-13	1.2(1.14-1.26)	6.33E-01	1.02(0.95-1.08)	1.92E-09	1.12(1.08-1.17)	3.29E-05
	rs3747517	2	163128824	C	0.726	0.311	1.53E-01	1.04(0.99-1.1)	2.96E-04	0.91(0.86-0.96)	1.12E-01	0.97(0.94-1.01)	3.81E-04
	rs2111485	2	163110536	G	0.611	0.174	1.02E-07	1.19(1.12-1.27)	1.59E-01	1.1(0.96-1.25)	7.20E-08	1.17(1.11-1.24)	2.52E-01
	rs17716942	2	163260691	T	0.868	NA	7.80E-04	1.18(1.07-1.3)	NA	NA	7.80E-04	1.18(1.07-1.3)	1.00E+00
**Conditioning on rs1990760**	rs3747517	2	163128824	C	0.726	0.311	2.87E-11	0.75(0.69-0.81)	5.16E-09	0.8(0.74-0.86)	1.52E-18	0.77(0.73-0.82)	3.26E-01
	rs2111485	2	163110536	G	0.611	0.174	5.09E-01	1.09(0.84-1.42)	2.12E-01	0.81(0.57-1.13)	8.08E-01	0.97(0.79-1.2)	1.65E-01
	rs17716942	2	163260691	T	0.868	NA	1.62E-01	1.08(0.97-1.2)	NA	NA	1.62E-01	1.08(0.97-1.2)	1.00E+00
**Multivariate analysis**	rs1990760	2	163124051	T	0.614	0.188	9.45E-23	1.48(1.37-1.6)	2.85E-06	1.24(1.13-1.35)	1.39E-25	1.37(1.29-1.45)	1.99E-02
	rs3747517	2	163128824	C	0.726	0.311	2.69E-11	0.75(0.69-0.81)	4.23E-09	0.80(0.74-0.86)	1.17E-18	0.77(0.73-0.82)	6.52E-01

Chr: chromosome; BP.B37: position based on NCBI build 37; RA: risk/non-risk alleles; RAF: risk allele's frequencies; EUR: European samples; CHN: Chinese samples; P: association P values in the meta, trans-ethnic meta analysis or the conditional study (alleles dosages in GWAS studies); OR: odds ratio; 95% CI: 95% confidence interval; p_Q: heterogeneity P values.

There were two independent loci identified in our study (Caucasian): rs1990760, rs3747517. The two reported independent loci: rs17716942 and rs2111485, published by Lam et al (Nature Genetics, Nov 2012) are tagged by rs1990760.

Supplementary Table 5b: Haplotypic association for markers in *IFIH1* locus with the risk of psoriasis

			Combined European				Combined Chinese				Combined All		
**Haplotype**	**rs1990760**	**rs3747517**	**Freq_contrl**	**Freq_case**	**OR (95% CI)**	**P**	**Freq_contrl**	**Freq_case**	**OR (95% CI)**	**P**	**OR (95% CI)**	**P**	**p_Q**
1	C	T	27.69%	27.13%	-	-	68.22%	69.94%	-	-	-	-	-
2	C	C	12.79%	9.27%	0.7(0.64-0.78)	4.66E-12	13.05%	10.91%	0.81(0.75-0.87)	2.85E-08	0.77(0.72-0.8)	7.90E-18	3.26E-02
3	T	C	59.50%	63.58%	1.04(0.97-1.1)	2.85E-01	18.73%	19.12%	0.98(0.92-1.04)	4.76E-01	1.01(0.96-1.1)	8.18E-01	2.06E-01
4	T	T	0.02%	0.01%	0.59(0.04-8)	6.92E-01	0.00%	0.03%	NA	8.60E-01	0.59(0.04-8)	6.95E-01	8.53E-01

Haplotype 1 was set as reference. Freq_contrl/Freq_cases: the haplotype frequency in controls/cases; *P*: association *P* values for each haplotype in specified population; OR: odds ratio; 95% CI: 95% confidence interval; p_Q: heterogeneity *P* values.

Supplementary Table 5c: The pairwise linkage disequilibrium among the three SNPs in *IFIH1* locus

SNP	rs2111485		rs17716942		rs3747517	
	CEU	JPT+CHB	CEU	JPT+CHB	CEU	JPT+CHB
rs1990760	r^2 = 0.826, D’ = 0.925	r^2 = 0.78, D’ = 1.00	r^2 = 0.20, D’ = 0.85	r^2 = 0.001, D’ = 1.00	r^2=0.409, D'=1.00	r^2=0.453,D'=1.00

* The Linkage disequilibrium was calculated based on the samples from CEU population and JPT, CHB populations in HapMap.

Supplementary Table 6: Conditional association results of *ERAP1/ERAP2* gene region

					RAF		Combined European		Combined Chinese		Combined All		
**Analysis**	**rsid**	**Chr**	**BP.B37**	**RA**	**EUR**	**CHN**	**P**	**OR (95% CI)**	**P**	**OR (95% CI)**	**P**	**OR (95% CI)**	**p_Q**
**Unconditional**	rs30376	5	96120259	C	0.286	0.503	5.31E-05	1.12(1.06-1.18)	1.84E-09	1.16(1.11-1.22)	7.23E-13	1.14(1.1-1.18)	3.21E-01
	rs2910686	5	96252589	C	0.424	0.398	7.24E-03	1.07(1.02-1.13)	6.65E-05	0.9(0.86-0.95)	3.15E-01	0.98(0.95-1.02)	2.57E-06
**Conditioning on rs30376**	rs2910686	5	96252589	C	0.424	0.397	1.21E-06	1.15(1.09-1.22)	6.74E-01	0.99(0.93-1.05)	1.37E-03	1.07(1.03-1.12)	3.43E-04

Chr: chromosome; BP.B37: position based on NCBI build 37; RA: risk/non-risk alleles; RAF: risk allele's frequencies; EUR: European samples; CHN: Chinese samples; *P*: association P values in the meta, trans ethnic meta analysis or the conditional study (alleles dosages in GWAS studies); OR: odds ratio; 95% CI: 95% confidence interval; p_Q: heterogeneity P values.

There were two independent signal identified: rs30376 (*ERAP1*, tagged rs27432), rs2910686 (*ERAP2*, reported by Lam et al (Nature Genetics, Nov 2012)). The secondary locus is showing association evidence in Caucasian only.

Supplementary Table 7: The association results of *HLA* region in Caucasian and Chinese samples

Condition on	Variant	Caucasians			Chinese		
		**MAF all**	**OR (95% CI)**	**P**	**MAF all**	**OR (95% CI)**	**P**
None	HLA-C*0602	14.80%	4.00 (3.62-4.41)	3.05×10^{-167}	13.80%	11.3(9.05-14.11)	2.09×10^{-101}
HLA-C*0602	HLA-A*0207	0	Absent	Absent	10.70%	2.99 (2.54-3.51)	5.30×10^{-40}
HLA-C*0602 + HLA-A*0207	AA_B_67	8 to 42%	omnibus	4.31×10^{-25}	8 to 54%	omnibus	8.96×10^{-25}
HLA-C*0602 + HLA-A*0207 + AA_B_67	HLA-A*0201	31.40%	1.26 (1.17-1.36)	3.62×10^{-10}	13.10%	1.23 (1.03-1.49)	0.026
	AA_A_114	2 to 43%	omnibus	2.46×10^{-5}	8 to 54%	omnibus	6.04×10^{-13}
HLA-C*0602 + HLA-A*0207 + AA_B_67 + 1 variant	rs9265656	14.30%	1.64 (1.39-1.93)	5.27×10^{-9}	-	not imputed	not imputed
	HLA-B*07	11.70%	0.60 (0.47-0.75)	1.13×10^{-5}	1.20%	0.70 (0.40-1.21)	0.2
	rs3131857	39.40%	1.02 (0.95-1.10)	0.56	22.60%	1.63 (1.36 -1.95)	9.76×10^{-8}
	AA_A_144	20%	0.95 (0.86-1.04)	0.23	22.60%	0.65 (0.55-0.78)	1.55×10^{-6}

MAF: minor allele frequency; OR: odds ratio; 95% CI: 95% confidence interval. *P*: association P values in the meta, trans ethnic meta analysis or the conditional study.

Supplementary Table 8: Haplotypic association for markers in *IL23R* locus with the risk of psoriasis

			Combined European				Combined Chinese				Combined All		
**Haplotype**	**rs2295359**	**rs12564022**	**Freq_contrl**	**Freq_case**	**OR (95% CI)**	**P**	**Freq_contrl**	**Freq_case**	**OR (95% CI)**	**P**	**OR (95% CI)**	**P**	**p_Q**
1	A	C	33.06%	32.93%	-	-	31.75%	34.98%	-	-	-	-	-
2	A	T	1.26%	2.16%	1.53(1.28-1.83)	4.51E-06	2.58%	2.53%	0.87(0.74-1.01)	7.27E-02	1.1(0.98-1.24)	1.03E-01	1.09E-08
3	G	C	38.88%	35.22%	0.88(0.84-0.93)	6.31E-06	10.33%	8.78%	0.74(0.68-0.81)	7.04E-11	0.84(0.81-0.88)	4.70E-13	7.64E-03
4	G	T	26.80%	29.69%	1.06(1-1.13)	3.83E-02	55.33%	53.71%	0.88(0.84-0.93)	5.86E-06	0.96(0.93-1)	5.44E-02	2.35E-05
4	G	T	26.80%	29.69%	-	-	55.33%	53.71%	-	-	-	-	-
2	A	T	1.26%	2.16%	0.88(0.73-1.05)	1.64E-01	2.58%	2.53%	0.97(0.83-1.14)	7.35E-01	0.93(0.83-1.05)	2.48E-01	2.29E-06
3	G	C	38.88%	35.22%	0.83(0.77-0.9)	1.94E-06	10.33%	8.78%	0.84(0.77-0.91)	5.01E-05	0.83(0.79-0.88)	4.11E-10	6.84E-05
1	A	C	33.06%	32.93%	0.91(0.84-0.99)	2.46E-02	31.75%	34.98%	1.13(1.07-1.19)	5.86E-06	1.06(1.01-1.11)	1.24E-02	8.08E-08

Haplotype 1 was set as reference. Freq_contrl/Freq_cases : the haplotype frequency in controls/cases; *P:* association P values for each haplotype in the specified population ; OR: odds ratio; 95% CI: 95% confidence interval; p_Q: heterogeneity P values.

Supplementary Table 9: Results of gene function prediction analysis in 80000 gene expression profiles of identified genes

Gene	Catalog	Term	P-value	Annotated
TP63	1	epidermis development	1.79E-31	YES
TP63	1	skin development	5.63E-20	YES
RUNX1	1	negative regulation of alpha-beta T cell differentiation	2.35E-07	NO
RUNX1	1	negative regulation of immune effector process	2.00E-06	NO
RUNX1	1	regulation of epidermis development	2.61E-06	NO

Phenotypic annotations are obtained from the Mouse Genetics Initiative database (www.informatics.jax.org). Table lists only genes and phenotypic annotations directly related to skin or epidermis development or immunological regulation. P-values refer to the correlation between the Gene principal component profile and the reconstituted phenotypic annotation principal component profile, uncorrected for multiple testing; all reported terms meet False Discovery Rate < 0.05. The Annotated column indicates if the gene has previously been linked to a specific mouse phenotype (Y) or not (N). Results are sorted alphabetically by gene name. The catalog means category of pathway. Pathway terms originate from 6 databases: 1. Gene Ontology Biological Processes; 2. Gene Ontology Cellular Component; 3. Gene Ontology Molecular Function; 4. KEGG; 5. BioCarta; and 6. Reactome.

Supplementary Table 10: The correlation between *TP63* gene and tissue expression

Tissue	No of samples	AUC	P-value
Skin	545	0.89	5×10^{-216}
Keratinocytes	48	0.85	2×10^{-17}
Epithelium	183	0.78	9×10^{-39}

Results of the tissue, organ and tissue type specific expression analysis in 80,000 gene expression profiles. The expression profiles were annotation into tissues, organs, or cell types using the MeSH database (http://www.nlm.nih.gov/mesh/). Table lists only genes in which show high expression in skin or specific skin relevant cells. Sample count specifies the number of expression profiles annotated with given annotation. AUC (area under the curve) gives the estimate how much of the variation on given gene expression profile is explained by a given tissue, organ or tissue type. *P*-values refer to enriched expression for a given gene in specific tissue, organ or tissue type compared to all other annotation terms.

Supplementary Table 11: The GO Enrichment Analysis of Implicated genes.

Term	Genes	P
GO:0002682:regulation of immune system process	DDX58, ERAP1, IRF4,IL12B, IL13, IL28RA, NOS2,NFKBIA,**RUNX1**, ETS1	4.14E-06
GO:0050776:regulation of immune response	DDX58, ERAP1, IRF4, IL12B, IL13, IL28RA, NOS2, NFKBIA	8.96E-06
GO:0048518:positive regulation of biological process	DDX58, KLF4, TRAF3IP2, CARD14, ERAP1,IRF4,IL12B,IL13, ILF3, NOS2, NFKBIA,PSMA6, **RUNX1**, RUNX3, STAT3, TNFRSF9, **TP63**, ETS1,REL, ZMIZ1	1.14E-05
GO:0002376:immune system process	DDX58, TRAF3IP2, ERAP1, ERAP2, IFIH1, IRF4,IL12B, IL13, IL23R, IL23A, NOS2, NFKBIA, **RUNX1,** ETS1	1.49E-05
GO:0010604:positive regulation of macromolecule metabolic process	KLF4, CARD14,IRF4,IL12B, ILF3, NFKBIA, PSMA6, **RUNX1**, STAT3, **TP63**, ETS1, REL, ZMIZ1	1.68E-05
GO:0031325:positive regulation of cellular metabolic process	KLF4, CARD14,IRF4,IL12B, ILF3, NFKBIA, PSMA6, **RUNX1,** STAT3, **TP63**, ETS1, REL, ZMIZ1	2.19E-05
GO:0045893:positive regulation of transcription, DNA-dependent	KLF4, IRF4, ILF3, NFKBIA, **RUNX1,** STAT3, **TP63,** ETS1,REL, ZMIZ1	2.31E-05
GO:0051254:positive regulation of RNA metabolic process	KLF4, IRF4, ILF3, NFKBIA, **RUNX1,** STAT3, **TP63,** ETS1,REL, ZMIZ1	2.47E-05
GO:0009893:positive regulation of metabolic process	KLF4, CARD14, IRF4, IL12B, ILF3, NFKBIA, PSMA6, **RUNX1,** STAT3, **TP63,** ETS1, REL, ZMIZ1	3.44E-05
GO:0009607:response to biotic stimulus	DDX58, ERAP1, IFIH1, IL23A, NOS2, NFKBIA, STAT2, SOCS1, **TP63**	3.46E-05
GO:0051707:response to other organism	DDX58, ERAP1, IFIH1, IL23A, NOS2, NFKBIA, STAT2, SOCS1	4.47E-05
GO:0010557:positive regulation of macromolecule biosynthetic process	KLF4, IRF4,IL12B, ILF3, NFKBIA, **RUNX1,** STAT3, **TP63,** ETS1, REL, ZMIZ1	4.65E-05
GO:0031328:positive regulation of cellular biosynthetic process	KLF4, IRF4,IL12B, ILF3, NFKBIA, **RUNX1**, STAT3, **TP63**, ETS1, REL, ZMIZ1	6.86E-05
GO:0006955:immune response	DDX58, TRAF3IP2, ERAP1, ERAP2, IFIH1, IL12B, IL13, IL23R, IL23A, NOS2, ETS1	7.29E-05
GO:0009891:positive regulation of biosynthetic process	KLF4, IRF4, IL12B, ILF3, NFKBIA, **RUNX1,** STAT3, **TP63,** ETS1, REL, ZMIZ1	7.75E-05
GO:0045941:positive regulation of transcription	KLF4, IRF4, ILF3, NFKBIA, RUNX1, STAT3, **TP63,** ETS1,REL, ZMIZ1	8.52E-05

The column of Genes lists the genes in each GO term from the input genes. The novel genes in our study were underlined and bonded. The *P* indicates the Fisher’s exact test result.
